# A Computational Hypothesis for Allostasis: Delineation of Substance Dependence, Conventional Therapies, and Alternative Treatments

**DOI:** 10.3389/fpsyt.2013.00167

**Published:** 2013-12-19

**Authors:** Yariv Z. Levy, Dino J. Levy, Andrew G. Barto, Jerrold S. Meyer

**Affiliations:** ^1^School of Computer Science, University of Massachusetts Amherst, Amherst, MA, USA; ^2^Recanati Faculty of Management, Tel-Aviv University, Tel-Aviv, Israel; ^3^Sagol School of Neuroscience, Tel-Aviv University, Tel-Aviv, Israel; ^4^Department of Psychology, University of Massachusetts Amherst, Amherst, MA, USA; ^5^Neuroscience and Behavior Program, University of Massachusetts Amherst, Amherst, MA, USA

**Keywords:** drug addition, allostasis, reward system adaptations, antireward system adaptations, mood, drug intake prediction, multiscale computational model, knowledge repository model

## Abstract

The allostatic theory of drug abuse describes the brain’s reward system alterations as substance misuse progresses. Neural adaptations arising from the reward system itself and from the antireward system provide the subject with functional stability, while affecting the person’s mood. We propose a computational hypothesis describing how a virtual subject’s drug consumption, cognitive substrate, and mood interface with reward and antireward systems. Reward system adaptations are assumed interrelated with the ongoing neural activity defining behavior toward drug intake, including activity in the nucleus accumbens, ventral tegmental area, and prefrontal cortex (PFC). Antireward system adaptations are assumed to mutually connect with higher-order cognitive processes occurring within PFC, orbitofrontal cortex, and anterior cingulate cortex. The subject’s mood estimation is a provisional function of reward components. The presented knowledge repository model incorporates pharmacokinetic, pharmacodynamic, neuropsychological, cognitive, and behavioral components. Patterns of tobacco smoking exemplify the framework’s predictive properties: escalation of cigarette consumption, conventional treatments similar to nicotine patches, and alternative medical practices comparable to meditation. The primary outcomes include an estimate of the virtual subject’s mood and the daily account of drug intakes. The main limitation of this study resides in the 21 time-dependent processes which partially describe the complex phenomena of drug addiction and involve a large number of parameters which may underconstrain the framework. Our model predicts that reward system adaptations account for mood stabilization, whereas antireward system adaptations delineate mood improvement and reduction in drug consumption. This investigation provides formal arguments encouraging current rehabilitation therapies to include meditation-like practices along with pharmaceutical drugs and behavioral counseling.

## Introduction

The principle of allostasis was established to enhance the homeostatic model whereby the well-balanced functional state of a living being is sustained by the constant conservation of the organism’s inner environment. Each divergence from the normal state of the organism is counterbalanced by negative feedback mechanisms which support the reinstatement of original setpoints. Instead, the allostatic model advances that the internal state of the organism continuously adapts to the surrounding natural world, attaining functional stability through the adaptation of physiological thresholds (1). As defined by Sterling and Eyer, “*allostasis provides for continuous re-evaluation of need and for continuous readjustment of all parameters toward new setpoints*” (1). In humans, this continuous adaptation to the environment is reached by means of neural and endocrine processes that are able to take priority over homeostatic regulations (1). The present paper is concerned with understanding and modeling the role of allostasis in drug addiction.

According to the hypothesis put forward by Koob and colleagues, a drug addict attains the allostatic state through the chronic deviation of their hedonic baseline. The addict’s physiological state is maintained operative by means of this affective adaptation, rather than by reinstatement of the original homeostatic balance. Symptoms of allostasis are manifested as changes in the addict’s mood or state of mind (2). The concept of allostasis accounts for the transitions in the gradual development of a dependency whereby drugs are first experienced to “*feel high*” but subsequently to “*feel normal*.” The allostatic framework of addiction relies on changes observed in the subject’s nervous and endocrinal systems which occur as addiction perpetuates, causing continuous and progressive distortions of the subject’s affective state (3). The *raison d’être* of these distortions is to guarantee the functional stability of the organism while its hedonic homeostatic state is corrupted.

The hedonic effect of an addictive substance on the brain’s reward system is orchestrated by within-system neuroadaptations and between-system neuroadaptations (4). Experimental observations show that rats undergo a continuous degradation of hedonic valence during extended periods of cocaine consumption (5). Similarly, the negative hedonic valence of the individual is increased by within-system and between-system neuroadaptations, and chronically impacts the person’s mood (2). Initial drug consumption disrupts the normal synaptic physiology of the reward system (6) which aims to reinstate its equilibrium by means of within-system neuroadaptations (4). Within-system adaptations act at the molecular or cellular levels that define the brain reward circuitry (7) and increase the magnitude of the ideal threshold of the reward (8). For instance, if the effect of the consumed drug of abuse relies on the availability of a particular neurotransmitter, within-system adaptations will diminish its amount within the reward system (9). With repeated drug administration the reward system becomes accustomed to extended activations of the within-system component, which eventually causes withdrawal symptoms during periods of abstinence (4). Dopamine in the nucleus accumbens (NAc) and extended amygdala plays an important role in within-system adaptations (10). As consumption further advances, the brain’s expectation for future rewards increases and within-system neuroadaptations become progressively inadequate and eventually fail to provide the individual with a well-adjusted functional state.

Due to deficiencies of within-system adaptations, brain structures different than the ones defining the reward system are recruited through the deployment of between-system neuroadaptations to further counterbalance the effect of the drug (4). These brain structures, delineated by Koob and Le Moal, embody the “*antireward systems*” (11). Between-system adaptations increase the baseline reward threshold (8) and originate in the brain stress system (4). For example, if a drug has a particular effect on the reward circuit, between-system activations could promote a hormonal response leading to the opposite effect (9). The corticotropin-releasing factor operating in the amygdala, stria terminalis, and ventral tegmental area (VTA) plays an important role in between-system adaptations (10).

The current investigation was undertaken to further explore the allostatic theory of addiction and combines two existing computational models: (i) a pharmacological model which describes how decreased reward function induces compulsive cocaine self-administration in rats (8) and (ii) a dynamical system model that represents hypotheses in terms of behavior, cognition, and neuropsychology, occurrences of spontaneous remissions from drug use in humans (12). More specifically, these models are connected by two computational hypotheses and one provisional assumption. The first hypothesis outlines within-system neuroadaptations, the second hypothesis delineates between-system neuroadaptations, and the provisional assumption relates to the mood of a virtual subject. In particular, within-system adaptations reflect changes in the reward system, whereas between-system adaptations embody modification in the antireward system. Mood estimation relies on the direct effect of the drug and its influence on these two neuroadaptations.

## Methods: Computational Framework for Allostasis

**Figure 1:**
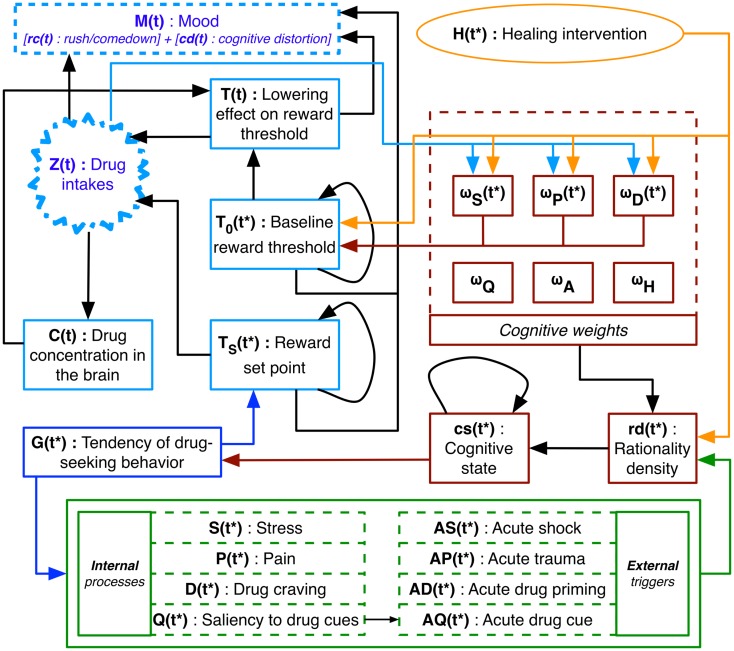
**Diagram of the computational model**. Time units differ: *t* is in minutes and t* in hours. Output *M*(*t*) is the mood estimation within the allostatic framework which combines the rush/comedown effect of the drug, *rc*(*t*), with the virtual subject’s cognitive distortion, *cd*(*t*). Levels of observations include the neuropsychological scale in green, the cognitive scale in red, and the healing scale in orange (12), which are connected to the expanded PK/PD model (8) in light blue. The cognitive weights, which modulate the ongoing neural activity on the neuropsychological scale, define the tendency of drug-seeking behavior, *G*(t*). This predisposition influences the reward set point, *T*_S_(t*), which together with the lowering effect on reward threshold, *T*(*t*), defines decisions about drug intake, *Z*(*t*). The cognitive weights influence the baseline reward threshold, *T*_0_(t*), indirectly influence *T*(*t*), and are affected by *Z*(*t*). A healing intervention has a direct impact on both cognitive learning and *T*_0_(t*), and an indirect effect on *T*_S_(t*) associated with changes in the virtual subject’s rationality density, *rd*(t*). The mood *M*(*t*) is a combination of *Z*(*t*), *T*(*t*), *T*_0_(t*), and *T*_S_(t*).

Figure 1 represents the model at the center of this investigation. The color code denotes different scales of observation: behavior (blue), neuropsychology (green), cognition (red), healing (orange), and pharmacology (light blue). The outputs of this discrete-time model reside in the pharmacological scale which includes computational predictions of drug intakes, *Z*(*t*), and mood, *M*(*t*). The processes composing the model are estimated over time-scales of minutes (*t*) and hours (t*). With the exception of *Z*, which is a binary variable, all other variables have continuous values with units of measurements that are left unspecified since corresponding human measurements are not available at this time. The current section describes the two hypotheses, the provisional assumption, and the dynamical system model at the center of the present investigation. Formal definitions of the processes in Figure 1 are included in the Supplementary Material.

The first hypothesis identifies reward system adaptations with ongoing neural activities within NAc, VTA, and prefrontal cortex (PFC); the second hypothesis associates antireward system adaptations with higher-order cognitive processes within PFC, orbitofrontal cortex (OFC), and anterior cingulate cortex (ACC); and the provisional assumption considers the virtual subject’s mood as a combination of reward components. Hypotheses and the provisional assumption reside in the pharmacological scale of the framework which extends the pharmacokinetic/pharmacodynamic (PK/PD) model of allostasis for laboratory rats of Ahmed and Koob (8). The PK component includes the drug concentration in the brain, *C*(*t*), and represents how the rat’s bloodstream and brain absorb the substance. The PD component includes the lowering effect on reward threshold, *T*, and the reward set point, *T*_S_, accounting for the threshold-lowering effect of the drug. *T* depends on the baseline reward threshold, *T*_0_, which is a constant value in Ref. (8). The thresholds *T, T*_S_, and *T*_0_, are the reward components associated respectively with the inverse variation of the brain’s reward sensitivity, the drug’s evolving acute effect which is reminiscent of the intracranial self-stimulation paradigm (13), and the minimal drug effect providing the individual with a reliable outcome (“*feel high*”). The decision-making process defining the animal’s future drug self-administration is controlled by the negative hedonic valence induced by the substance, arithmetically *T*-*T*_S_. In the present model these thresholds change over time and are respectively denoted *T*(*t*), *T*_S_(t*), and *T*_0_(t*).

Within-system alterations are represented by changes in the drug potency index, *T*_*50*_, and between-system adjustments are described by variations in the baseline reward threshold, *T*_0_. Ahmed and Koob (8) successfully replicate patterns of intravenous cocaine self-administration observed in laboratory rats, while relying on values of constant magnitudes to represent the within-system and the between-system adaptations. We translate the model of Ahmed and Koob toward human application by mathematically describing within- and the between-system components as explicit time-dependent functions, and by providing an estimation of the virtual subject’s mood. The pharmacological (light blue) scale of the framework pictured in Figure 1 is an extended version of the PK/PD model discussed in Ref. (8).

### Hypothesis 1: Within-system neuroadaptations (pharmacological scale)

Ahmed and Koob (8) emulate within-system neuroadaptations by means of the drug potency index, *T*_*50*_, one of the constant parameters used to define *T*. The present study complements this interpretation by considering a subsequent report of the same authors (14) about cocaine intake escalation in rats. Ahmed and Koob observed rats evolving in environments with different regimes of cocaine availability, compared the animals’ drug intake patterns, and suggested that the rat’s reward system homeostatic set point deteriorates gradually rather than abruptly (14).

Along those lines, our model is based on the following hypothesis: within-system adaptations are expressed by the reward set point, *T*_S_, which deteriorates for maladaptive behaviors and improves for healthy behaviors. More specifically, within-system neuroadaptations depend upon the virtual subject’s current attitude toward drug use, *G*(t*), which emulates the translation of ongoing neural activities within the NAc, VTA, and PFC into behavior. Computationally, after the first drug intake, *T*_S_ is assumed to monotonically increase for healthy behavior and exponentially decrease for maladaptive behavior:

(1) $$T_{S}\left(t^{*}+1\right)=\left\{\begin{array}{cc} \begin{array}{c} \lambda \cdot \left(1-e^{-\beta \cdot d}\right)\\ \;+T_{S}\left(t_{c}\right) \end{array}\; & \mathrm{if}\;G\left(t^{*}\right)\geq 0\text{ and }\sum Z\geq 1\\ T_{S}\left(t_{c}\right)\cdot e^{-\gamma \cdot d}\; & \mathrm{if}\;G\left(t^{*}\right)<0\text{ and }\sum Z\geq 1\\ T_{S}\left(t^{*}\right)\; & \mathrm{otherwise,} \end{array}\right.$$

where λ, β, and γ, are constants; *t*_c_ corresponds to the last time t* when *G* changed its sign; *d* is a temporal unit-step counter reset to 0 when *G* changes its sign, ensuring mathematical continuity to *T*_S_; and Σ*Z* ≥ 1 denotes that at least one drug intake occurred up to time t*.

### Hypothesis 2: Between-system neuroadaptations (pharmacological scale)

Goldstein and Volkow (15) proposed the “*impaired response inhibition and salience attribution*” (I-RISA) theory of addiction while assessing the PFC significant impact on cognitive alterations in terms of maladaptive behavior toward drug abuse. This theory is based on observations in which addicts display significant differences from healthy individuals within the mesolimbic and mesocortical dopaminic pathways, as well as within the orbitofrontal and anterior cingulate cortices (15). These changes suggest that an addict is more susceptible to experience cognitive distortions. The Encyclopedia of Cognitive Behavior Therapy defines cognitive distortions as “*identifiable errors in thinking*” (16) which sustain pathologies related to alcohol and drug use (17) gambling (18, 19), eating (20), and Internet use (21).

Along those lines, our model considers the following hypothesis: between-system adaptations are expressed by the baseline reward threshold, *T*_0_, which increases when maladaptive behavior occurs and decreases during healthy behavior. After some drug intakes, cognitive weights which adapt to maladaptive behavior will cause *T*_0_ to increase. In case the virtual subject regains a healthy behavior, these cognitive weights cause *T*_0_ to decrease. More specifically, between-system alterations are defined as a function of cognitive time-dependent weights, ω_S_(t*), ω_P_(t*), and ω_D_(t*), and healing intervention, *H*(t*). Cognitive weights mimic associative learning between the drug and its pleasurable effect, whereas *H* emulates positive cognitive changes toward remission from drug intake. Computationally, after a number of drug intakes, the cognitive substrate of the virtual subject starts to stochastically influence *T*_0_ by means of ω_*S*_, ω_*P*_, ω_*D*_ and the healing intervention *H*. The modulating influence of ω_*S*_, ω_*P*_, and ω_*D*_ on *T*_0_ has opposite valence when a healing intervention occurs (*H* = 1) than when it does not (*H* = 0):

(2) $$T_{0}\left(t^{*}+1\right)=\left\{\begin{array}{cc} \begin{array}{c} T_{0}\left(t^{*}\right)+\delta_{T0}\cdot \left(-2\cdot H\left(t^{*}\right)+1\right)\\ \;\cdot \left(\omega_{S}\left(t^{*}\right)-\omega_{P}\left(t^{*}\right)+\omega_{D}\left(t^{*}\right)\right) \end{array}\; & \mathrm{if}\sum Z\geq \alpha \\ T_{0}\left(t^{*}\right)\; & \mathrm{otherwise,} \end{array}\right.$$

where δ_*T0*_ and α are constants, and Σ ≥ α denotes the period subsequent to at least α drug intakes.

### Provisional assumption: Mood (behavioral/pharmacological scale)

A classical validation schema, where simulations are compared to laboratory data is not suitable for the proposed model, as human measures of the considered processes are not currently available. Instead, for qualitative evaluation purposes, an additional provisional assumption is formulated to predict the subject’s mood, *M*, as an aggregate of neural and psychological components. The former component relies on the direct rush/comedown effect of the drug, *rc*(*t*), and the latter upon cognitive distortions, *cd*(*t*), emulated as a function of current and previous hedonic adaptations.

The evolution of *rc* is defined as the summation of piece-wise sinusoidal functions each of one period with slightly exponentially decaying tails that initiate when a drug intake occurs. Cognitive distortions related to addiction are assumed to depend on the overall current reward state of the virtual subject which includes *T, T*_S_, and *T*_0_. Healthy individuals should not suffer from cognitive distortions: no current drug’s effect on reward threshold (*T*) should arise, nor should any negative hedonic valences from within- and between-system neuroadaptations (*T*_S_ and *T*_0_). The speculative *cd* combines the current reward state of the individual, the current activation of within- and between-system neuroadaptations, alongside of previously experienced hedonic adaptations:

(3) $$\begin{array}{ccc} M\left(t\right) & =rc\left(t\right)+cd\left(t\right) & \\ \text{with}\;cd\left(t\right) & =-T\left(t\right)+\gamma_{M}\cdot \Delta TSO\left(t^{*}\right)\\ & \;-\Delta TSO\left(t^{*}-1\right)\;\mathrm{when}\;\sum Z\geq 1, \end{array}$$

where γ_*M*_ is constant; Σ*Z* ≥ 1 denotes that at least one drug intake occurred up to *t*; and Δ*TSO* stands for the arithmetic difference between *T*_S_ and *T*_0_. The formulation of *cd* is suggested by the temporal difference component employed in the first model of learning mechanism associated with dopaminergic neurons in the basal ganglia (22, 23).

### Behavioral, cognitive, and neuropsychological scales of the model

The tendency for drug-seeking behavior, *G*(t*), defines the behavioral scale of the model (in blue on Figure 1). Healthy behavior, i.e., avoidance of drug use, corresponds to positive values of *G*, and maladaptive behavior, i.e., a tendency toward drug-seeking behavior, corresponds to negative values. *G* assesses how neural activity of brain regions sensitive to addictive drugs affects the ratio of compulsion and inhibition (24). Compulsion is assessed according to the Robinson and Berridge “*incentive-sensitization*” theory of addiction (25), whereas inhibition is estimated through developmental and biosocial factors. Even though previously presented as time-dependent processes (24), in the present study the behavioral scale is simplified and includes compulsion and inhibition as constant parameters.

The cognitive apparatus of the virtual subject (in red on Figure 1) transforms the ongoing neuropsychological activities into information accessible at the behavioral scale. The ratio of compulsion and inhibition is driven by the cognitive state, *cs*(t*), which is a mathematical transformation into the real interval [0, 1] of the virtual subject’s rationality density, *rd*(t*). A more compulsive behavior is expressed when *cs* is equal to 0, whereas a stronger inhibitory behavior is expressed when *cs* is equal to 1. In terms of economic theories of addiction, *cs* and *rd* delineate whether the virtual subject is irrational, imperfectly rational, or rational (26). The process *rd* brings together the neuropsychological processes and adjusts them through their correspondent cognitive weights, which include a set of time-varying processes ω_*S*_(t*), ω_*P*_(t*), ω_*D*_(t*), and constant parameters ω_*Q*_, ω_*A*_, and ω_*H*_. Time-dependent cognitive weights ω_*S*_, ω_*P*_, and ω_*D*_ stochastically adjust and predispose the virtual subject toward maladaptive behavior. The time-dependent cognitive weights mimic alterations in the PFC, OFC, and ACC that lead addicted persons and healthy individuals to manifest contrasting saliencies during affective events related to drug consumption (27, 28).

The neuropsychological activities (in green on Figure 1) encompass internal processes (*S, P, D*, and *Q*) and external triggers (*AS, AP, AD*, and *AQ*). The negative affective state of nervousness, anxiety, or stress, *S*(t*), of an addict expands during withdrawal phases (29) due to changes occurring in the brain reward and stress systems (2) including the VTA, the NAc, the amygdala, and the lateral hypothalamus (30). The level of burden or worry, *P*(t*), related to a person’s health state increases as a consequence of drug consumption (31). The intensity of drug craving, *D*(t*), strongly correlates with the level of extracellular dopamine in key brain areas. Animal experiments show how the concentration of dopamine in the NAc increases during acute drug consumption (32) and decreases during withdrawal (33). Human studies suggest that dopamine-related neural activity in the OFC and ACC intensifies under the influence of drugs (34), and diminishes during long-term withdrawal (15).

Severe stressors, *AS*(t*), such as electric foot-shocks for laboratory rats (35) and verbal scolding for humans (36), can lead to the reinstatement of maladaptive behavior. Acute distress events, *AP*(t*), such as non-fatal overdoses for injection drug users (37) or coronary heart disease for smokers (38) may cause the individual to rapidly cease using the substance. After a period of abstention, rats (39) and humans (40) exposed to drug priming, *AD*(t*), are more likely to stumble into relapse. Drug-associated cues linked to a particular environment, *AQ*(t*), can reactivate drug-seeking behavior (41). The magnitude, *Q*(t*), of these cues depends upon the drug-contingent neural mechanisms of learning and memory (42) that may facilitate the sensitization of incentive salience of drug cues leading to compulsive consumption (43).

Healing interventions (in orange on Figure 1), *H*(t*), can cause immediate abstinence by direct alteration of the virtual subject’s rationality *rd* and by adapting cognitive weights of internal and external processes. These modifications can endure over time and reflect real-life occurrences of “*maturing out*” of addiction (12). When active, healing interventions influence ω_*S*_, ω_*P*_, ω_*D*_, and *rd*, inclining the virtual subject toward healthy behavior. Once *H* becomes idle, residual cognitive effects on these weights stochastically become permanent. Different occurrences of *H* delineate replacement therapies (e.g., nicotine replacement therapy) or complementary treatments (e.g., mindfulness meditation). Both these techniques favorably support cigarette-smoking cessation (44, 45). The first is simulated with an active *H* lasting several days, whereas the second by a sequence of active *H*’s of much shorter durations.

As discussed above, the pharmacological scale (in light blue on Figure 1) includes the binary decision toward *Z* which depends on the arithmetic difference between the lowering effect on reward threshold, *T*(*t*), and the reward set point, *T*_S_(t*), similarly to the model in Ref. (8).

## Illustrative Results

The experimental set up includes mathematical definitions of the processes described above and detailed in the Supplementary Material, as well as their correspondent implementation in MATLAB^®^. The initial conditions of the presented simulations are defined by 71 parameters whose values, reported in Table S1 in Supplementary Material, are chosen according to Ref. (8) and (12) with few exceptions: where possible, parameters defining the simulations were chosen according to human studies. The rat brain apparent volume of distribution for cocaine used in Ref. (8) was replaced with an estimate for (S)-[^11^C]nicotine in humans (46). The number of drug intakes defining the initial associative learning reflected in ω_*S*_, ω_*P*_, ω_*D*_, as well as the constant α in Eq. 2, were chosen according to a clinical study by DiFranza at al. (47) which classifies the progression of physical addiction into four stages: none (stage 1), wanting (stage 2), craving (stage 3), and needing (stage 4). Associative learning is active until the virtual subject reaches the needing phase, and the constant α relates to the craving phase. For nicotine, the four stages correspond to consumption rates of 2.2 ± 3.4, 4.4 ± 5.0, 8.6 ± 7.1, and 13.2 ± 7.7 cigarettes per smoking day (47), respectively. The minimum amount of time separating consecutive drug intake of 4 s in Ref. (8) was changed to 30 min.

Three Case Studies based on the same experimental set up, and narrating tobacco smoking, are presented herein to exemplify the methods described in the previous section: (i) escalation from early to heavy smoking, (ii) conventional therapeutic process (e.g., nicotine patches), and (iii) alternative medical treatment (e.g., meditation). Case Study 1 stands for the evaluation baseline; Case Study 2 is identical but includes two long-lasting healing interventions, *H*; whereas Case Study 3 encompasses eight short-lasting healing interventions. The total amount of time for which *H* is active in these Case Studies is respectively 0, 10, and 5 days.

Each Case Study includes three Evaluations: in the first both *T*_S_ and *T*_0_ are time-dependent processes according to Eqs 1 and 2; in the second *T*_S_ is constant and *T*_0_ is time-dependent; and in the third *T*_S_ is time-dependent and *T*_0_ is constant. The simulation results include means for 100 runs and 95% simulation envelopes corresponding to a period of 160 days with the drug becoming available on the fifth day. Changes in the allostatic state of the virtual subject are estimated through variations of the mood *M*. The daily abstinence index is the ratio (as a percentage) between the runs in which the virtual subject don’t use the addictive drug and the total number of simulation runs. This index assesses the virtual subject’s stage according to the classification by DiFranza et al. (47).

### Case study 1: Allostatic state trajectory during escalation of drug consumption

**Figure 2:**
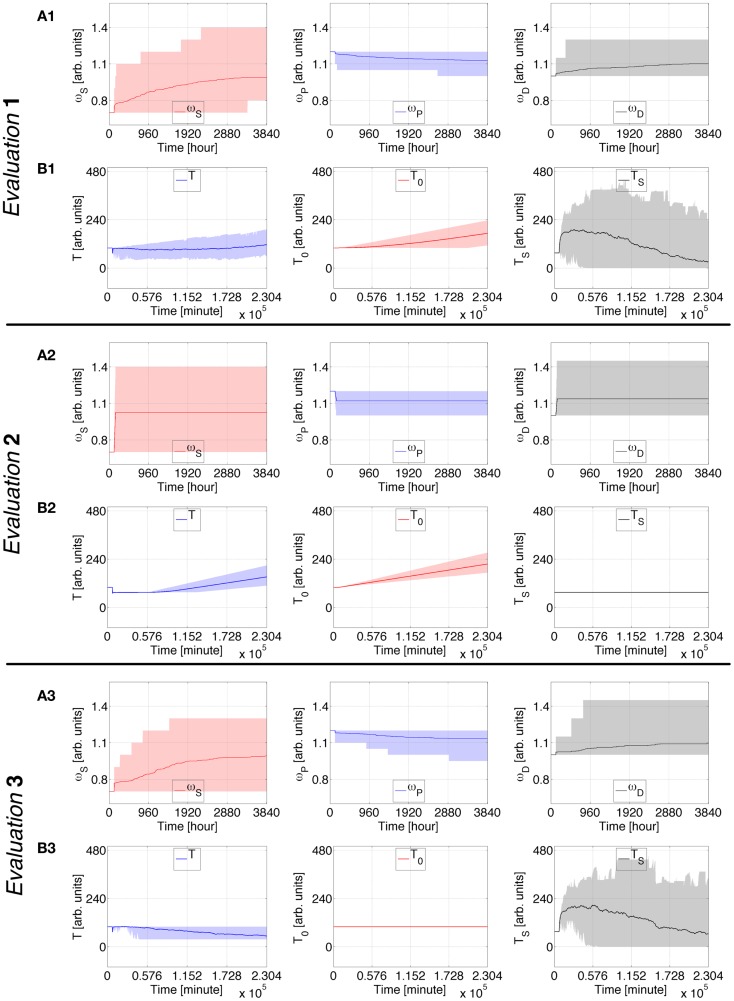
**Case Study 1 (cognitive weights and reward components)**. Simulations of virtual behavior for cigarette consumption over a period of 160 days. Cigarettes are available on the fifth day. Results are the average of 100 runs. Evaluation 1 simulates both *T*_S_ and *T*_0_ as time-dependent processes. *T*_S_ is constant and *T*_0_ time-dependent in Evaluation 2, *T*_S_ is time-dependent and *T*_0_ constant in Evaluation 3. **(A1–A3**) show the evolution of cognitive weights ω_*S*_, ω_*P*_, and ω_*D*_; and **(B1–B3)** the progression of *T, T*_0_, and *T*_S_. The shades correspond to 95% simulation envelopes. The time-scales are hours for **(A1–A3)**, and minutes for **(B1–B3)**. Further details of these simulations are reported in Figures S1 and S2 in Supplementary Material.

**Figure 3:**
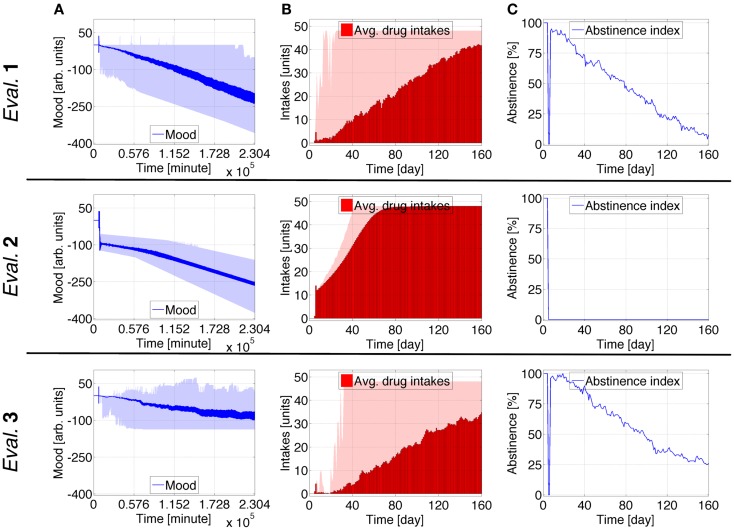
**Case Study 1 (mood and health state assessments)**. Simulations of virtual behavior for cigarette consumption over a period of 160 days. Cigarettes are available on the fifth day. Results are the average of 100 runs. Evaluation 1 simulates both *T*_S_ and *T*_0_ as time-dependent processes. *T*_S_ is constant and *T*_0_ time-dependent in Evaluation 2, *T*_S_ is time-dependent and *T*_0_ constant in Evaluation 3. **(A)** Shows the evolution of the subject’s mood *M*; **(B)** the average number of drug intakes; and **(C)** the abstinence index. The shades correspond to 95% simulation envelopes. The time-scales are minutes for **(A)**, and days for **(B,C)**. Further details of these simulations are reported in Figures S1 and S2 in Supplementary Material.

Figures 2 and 3 depict the behavior of a virtual subject who engages in cigarette-smoking 5 days after the simulation begins. In Evaluation 1, changes in ω_*S*_, ω_*P*_, and ω_*D*_ are gradual; *T* is at first weaker than *T*_S_ but eventually surpasses it, and *T*_0_ continually increases; *M* increasingly oscillates around its downslope; the average drug consumption increases and the abstinence index diminishes. Upon completion of the simulation, this virtual subject is characterized by an average consumption of ~42 intakes/day (~2 packs) and an abstinence index of ~11%, comparable to a severe stage 4 (47).

In Evaluation 2, the cognitive adaptations occur significantly faster than in Evaluation 1. *T* and *T*_S_ are approximately equal at first, but eventually *T* becomes larger than *T*_S_ as *T*_0_ constantly increases. *M* abruptly decreases during the loading phase and subsequently manifests a negative trend enclosed by minor oscillations. The average consumption quickly increases and the virtual subject reaches a satiety state of ~48 cigarettes/day. Note that the number of intakes defining satiety is not an explicit constraint defined in the model. The abstinence index is consistently at zero.

In Evaluation 3, the evolution of the virtual subject’s processes is very similar to the predictions for Evaluation 1, but *T* decreases and *M* has a less negative downslope. This evaluation ends with ~34 intakes/day and ~25% abstinence.

Additional details can be found in the Supplementary Material. Figures S1 and S2 in Supplementary Material include the fine details for Case Study 1, and Figures S3–6 in Supplementary Material show how different probabilities defining changes in ω_*S*_, ω_*P*_, and ω_*D*_ influence the predicted consumption rates and mood downslope. If the first smoked cigarette within the simulations is considered as the first ever in the life of the virtual subject, then Figures S3–6 in Supplementary Material can be considered to relate to different rates of progression from recreational smoking toward heavy smoking.

Case study 1 indicates that a virtual subject consuming drugs for the first time, or relapsing after a period of abstinence, undergoes a continuous negative shift in mood baseline which directly correlates with the strength of cognitive learning facilitating drug consumption. In addition, the virtual subject suffers growing mood swings during protracted consumption. When *T*_S_ is constant, the mood substantially decreases during the first number of drug intakes, and the virtual subject rapidly reaches the satiety consumption rate. With *T*_0_ constant, the simulated mood has a weaker negative tendency and oscillates less.

This Case Study shows that escalation in drug consumption occurs together with chronic depression of mood. When just between-system adaptations are operative, these simulations predict that the virtual subject’s mood strongly drops, whereas it moderately decreases when just within-system adaptations are operative.

### Case study 2: Allostatic state trajectory during conventional therapies

**Figure 4:**
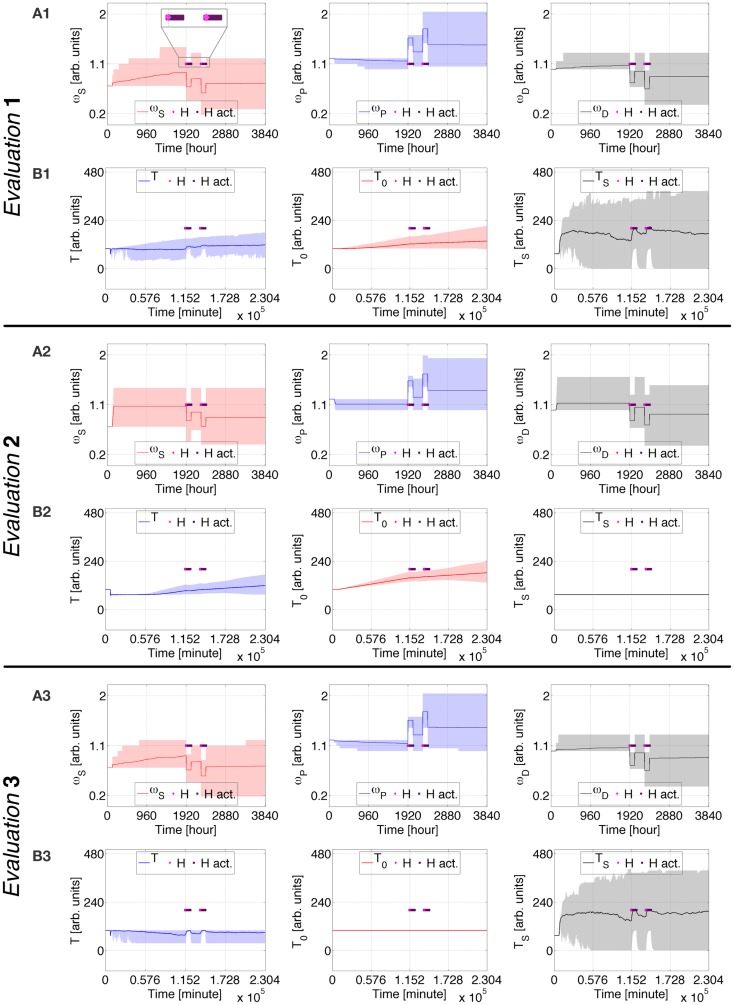
**Case Study 2 (cognitive weights and reward components)**. Simulations of virtual behavior for cigarette consumption over a period of 160 days. Cigarettes are available on the fifth day. Results are the average of 100 runs. Evaluation 1 simulates both *T*_S_ and *T*_0_ as time-dependent processes. *T*_S_ is constant and *T*_0_ time-dependent in Evaluation 2, *T*_S_ is time-dependent and *T*_0_ constant in Evaluation 3. In all evaluations, the recovery process *H* is activated at *t* = 1920 and *t* = 2280 [hours] (in light pink) and stays active for 120 h (dark pink). **(A1–A3)** show the evolution of cognitive weights ω_*S*_, ω_*P*_, and ω_*D*_; and **(B1–B3)** the progression of *T, T*_0_, and *T*_S_. The shades correspond to 95% simulation envelopes. The time-scales are hours for **(A1–A3)**, minutes for **(B1–B3)**. Further details of these simulations are reported in Figures S7 and S8 in Supplementary Material.

**Figure 5:**
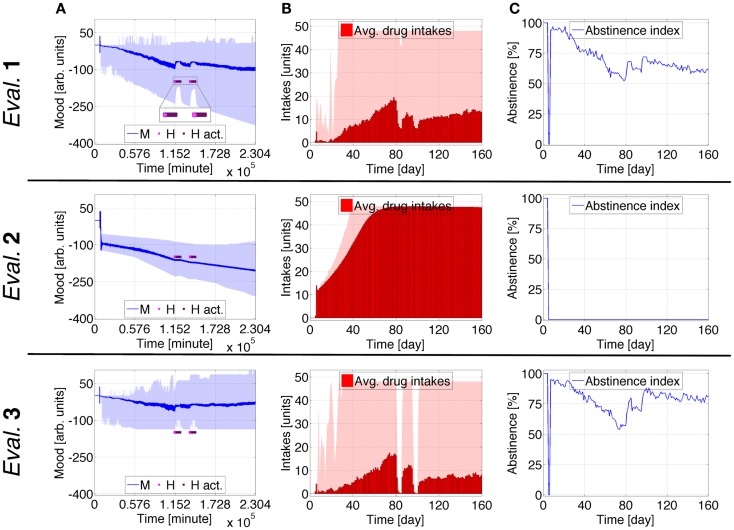
**Case Study 2 (mood and health state assessments)**. Simulations of virtual behavior for cigarette consumption over a period of 160 days. Cigarettes are available on the fifth day. Results are the average of 100 runs. Evaluation 1 simulates both *T*_S_ and *T*_0_ as time-dependent processes. *T*_S_ is constant and *T*_0_ time-dependent in Evaluation 2, *T*_S_ is time-dependent and *T*_0_ constant in Evaluation 3. In all evaluations, the recovery process *H* is activated at *t* = 1920 and *t* = 2280 [hours] (in light pink) and stays active for 120 h (dark pink). **(A)** shows the evolution of the subject’s mood *M*; **(B)** the average number of drug intakes; and **(C)** the abstinence index. The shades correspond to 95% simulation envelopes. The time-scales are minutes for **(A)**, and days for **(B,C)**. Further details of these simulations are reported in Figures S7 and S8 in Supplementary Material.

The profile presented in Figures 4 and 5 is similar to Case Study 1 but also includes healing interventions (*H*). Five-day long *H* events are activated at *t* = 1920 and *t* = 2280 [hours]. This is intended to emulate 25 days of a replacement therapy using a nicotine transdermal system for two 5 days periods separated from each other by 15 days. All evaluations for Case Studies 1 and 2 are similar until *H* is activated.

During the first therapeutic period in Figures 4 and 5, ω_*S*_, ω_*P*_, and ω_*D*_ are influenced by *H* to promote healthier behavior. This positive effect partially persists after the first period of therapy and is further strengthened by the second. In Evaluation 1, the activation of *H* causes a small upswing in *T*, a strong upswing in *T*_S_, and a decrease in the upslope of *T*_0_. The degradation of *M* becomes less accentuated after the treatment. The average drug consumption drops and the abstinence index rises while *H* is active. This experiment’s endpoint is comparable to an advanced stage 3 or an intermediate stage 4 (47), with a consumption rate of ~14 intakes/day and an abstinence index of ~62%. In Evaluation 2, both *T* and *T*_0_ reduce their upslope during the therapy, while the effect on *M* is negligible. The average consumption steadily increases ending at ~47 intakes/day, and the abstinence index drops to zero. In Evaluation 3, the progression of the virtual subject’s processes is similar to Evaluation 1 but somewhat slower. *M* stabilizes and becomes nearly constant after the therapy. This endpoint is ~8 intakes/day and ~81% abstinence.

Additional fine detail for Case Study 2 can be found in Figures S7 and S8 in Supplementary Material. Figures S9–12 in Supplementary Material show how different probabilities defining the influence of *H* affect the permanent predicted consumption rates. Higher probabilities lead the virtual subject to stage 1 or intermediate stage 2, whereas lower probabilities to advanced stage 4. The same sets of probabilities are tested when *T*_S_ is constant (Figures S13–16 in Supplementary Material) and when *T*_0_ is constant (Figures S17–20 in Supplementary Material). For *T*_S_ constant, the virtual subject always gets to a satiety consumption rate and reveals a slight shy positive trend in *M* for the highest probabilities along with a decrease in average consumption. For *T*_0_ constant, *M* becomes constant after the therapy and its variations become smaller as the tested probabilities become higher.

Case study 2 shows how a few, though long, healing interventions diminish the negative trend in the virtual addict’s mood. Early indications of mood increase appear when healing signals are highly effective. When *T*_S_ is constant, curative effects on the mood are negligible, unless cognitive learning is exceptionally successful. Even though positive, the influences on mood for this extreme case are quite limited. With *T*_0_ constant, the mood stabilizes after healing interventions and remains roughly constant.

This Case Study exemplifies the effect of prolonged healing interventions. When just between-system adaptations are operative, these simulations predict that the virtual subject’s mood continues to worsen in spite of therapeutic events. When just within-system adaptations are operative, the predicted mood stabilizes as a consequence of healing periods.

### Case study 3: Allostatic state trajectory during alternative medical treatments

**Figure 6:**
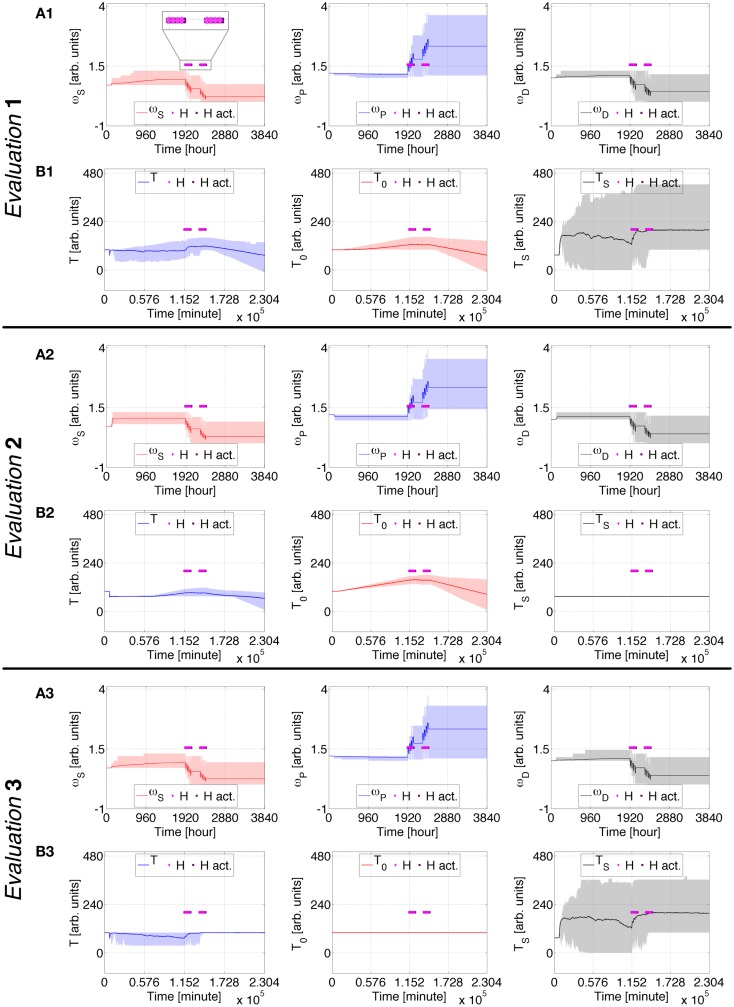
**Case Study 3 (cognitive weights and reward components)**. Simulations of virtual behavior for cigarette consumption over a period of 160 days. Cigarettes are available on the fifth day. Results are the average of 100 runs. Evaluation 1 simulates both *T*_S_ and *T*_0_ as time-dependent processes. *T*_S_ is constant and *T*_0_ time-dependent in Evaluation 2, *T*_S_ is time-dependent and *T*_0_ constant in Evaluation 3. In all evaluations, the recovery process *H* is activated at *t* ∈ {1920, 1960, 2000, 2040, 2280, 2320, 2360, 2400} [hours] (in light pink) and stays active for 15 h (dark pink). **(A1–A3)** show the evolution of cognitive weights ω_*S*_, ω_*P*_, and ω_*D*_; and **(B1–B3)** the progression of *T, T*_0_, and *T*_S_. The shades correspond to 95% simulation envelopes. The time-scales are hours for **(A1–A3**), and minutes for **(B1–B3)**. Further details of these simulations are reported in Figures S21 and S22 in Supplementary Material.

**Figure 7:**
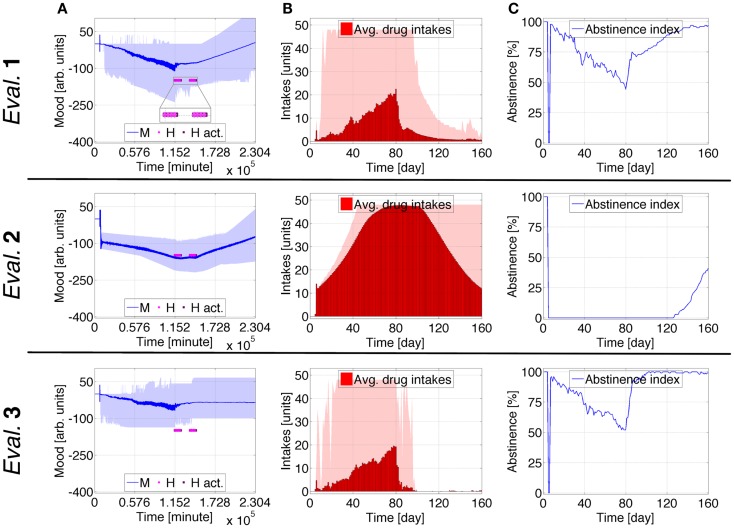
**Case Study 3 (mood and health state assessments)**. Simulations of virtual behavior for cigarette consumption over a period of 160 days. Cigarettes are available on the fifth day. Results are the average of 100 runs. Evaluation 1 simulates both *T*_S_ and *T*_0_ as time-dependent processes. *T*_S_ is constant and *T*_0_ time-dependent in Evaluation 2, *T*_S_ is time-dependent and *T*_0_ constant in Evaluation 3. In all evaluations, the recovery process *H* is activated at *t* ∈ {1920, 1960, 2000, 2040, 2280, 2320, 2360, 2400} [hours] (in light pink) and stays active for 15 h (dark pink). **(A)** shows the evolution of the subject’s mood *M*; **(B)** the average number of drug intakes; and **(C)** the abstinence index. The gray shades correspond to 95% simulation envelopes. The time-scales are minutes for **(A)**, and days for **(B,C)**. Further details of these simulations are reported in Figures S21 and S22 in Supplementary Material.

The profile in Figures 6 and 7 present a different type of healing intervention than in Case Study 2. At each of the simulated times *t* ∈ {1920, 1960, 2000, 2040, 2280, 2320, 2360, 2400} [hours], a 15 h *H* event is activated. This is intended to emulate two 5 day healing periods during which the virtual subject undergoes four meditation practices. The benefit of each practice lasts for 15 h, and the two healing periods are 10 days apart. Other than *H* event durations and activation times, all the parameters defining this case study are the same as in Case Studies 1 and 2.

During the first period of meditation, ω_*S*_, ω_*P*_, and ω_*D*_ strongly adjust. The enduring positive changes are additionally expanded after the second period of meditation. In Evaluation 1, *T* decreases before meditation, increases during meditation, and finally decreases again afterward. There is an upswing of *T*_S_ when *H* is active. *T*_0_ initially increases, becomes constant after the first healing session, and decreases after the second. After the first period of meditation, *M* stops declining, and after it increases with reduced oscillation. The average drug consumption significantly decreases after the first healing period and further decreases after the second. The abstinence index robustly increases during the treatment. The endpoint of this evaluation corresponds to ~1 intake/day and ~97% abstinence, placing the virtual subject in stage 1 or early stage 2 (47). In Evaluation 2, *T* variations are minor, and *T*_0_ is a wide bell-shaped curve whose maximal height corresponds to the healing periods. Activations of *H* first lead to stabilization of *M*, and then to its increase. The bell-shaped average drug consumption reaches its maximum and starts to decrease when *H* is active, finishing at ~12 intakes/day. The abstinence index starts to increase shortly after the end of the treatment, reaching ~40% at the end of the simulation. Evaluation 3 is comparable to Evaluation 1 but *M* stabilizes after the therapy rather than increasing, and its final state is characterized by <1 intake/day and ~99% abstinence.

Additional details for Case Study 3 can be found in the Supplementary Material. Figures S21 and S22 in Supplementary Material show the fine details. Figures S23–26 in Supplementary Material illustrate how different probabilities defining the influence of *H* permanently impact the predicted consumption rates. Higher probabilities lead the virtual subject to cease using the drug, whereas lower probabilities lead the subject to advanced stage 1 or intermediate stage 2. The same sets of probabilities are tested when *T*_S_ is constant (Figures S27–30 in Supplementary Material) and when *T*_0_ is constant (Figures S31–34 in Supplementary Material). For *T*_S_ constant, the virtual subject always gets to its satiety consumption rate, and after the treatment *M* increases. For *T*_0_ constant, *M* tends to become constant after the therapy and its variations become smaller as the probability becomes higher.

Case study 3 displays how phasic healing interventions increase the mood of the virtual addict. This increase becomes very conspicuous while healing signals have high effectiveness. Also, when *T*_S_ is constant, mood increases as a direct correlation of healing success. With *T*_0_ constant, after healing interventions the mood stabilizes and becomes approximately constant.

This case study exemplifies the effect of brief healing interventions that follow one another at short intervals. When just between-system adaptations are operative, these simulations predict that the virtual subject’s mood increases as a result of curative events. When just within-system adaptations are operative, the predicted mood stabilizes but does not improve after healing periods.

## Discussion

The computational framework for allostasis presented above unites and elaborates two earlier formal models (8, 12). The first relies on a closed-loop representation of the pharmacokinetics, the pharmacodynamics, and a decision-making process delineating future cocaine consumption in rats (8). The second is a dynamical system model that encompasses neuropsychological and cognitive elements to mimic human occurrences of natural recoveries (12). The allostatic theory of addiction comprises within-system and between-system neuroadaptations that influence the brain’s reward system: the former by a direct impact, and the latter by means of antireward system activations. The function of these adaptations is to balance the hedonic state of the addict and to provide the organism with a reasonable operational existence. Manifestations of the allostatic state come through mood alterations (2, 7).

The present article is an exploratory instance of knowledge repository (KR) modeling for addiction (48) which investigates cognitive correlates of the allostatic theory. A KR model comprises a collection of empirical observations that are mathematically translated and unified to predict the natural course of an entity (48). This class of models promotes the identification of plausible hypotheses which, if experimentally tested, could provide pertinent knowledge to further improve the computational framework. The repetition of this investigative process initiates a hypothesis-driven sequence of experiments supporting translational research (49). The present model assembles building blocks of neuropsychology, cognition, and behavior into a multiscale computational framework aiming to facilitate rational entailments of the allostatic theory.

The computational description of a complex phenomenon such as drug use and abuse requires finding a compromise between two desirable but incompatible objectives. On the one hand, the biological components defining the model embrace a simplified ontology of addiction, and on the other hand, the mathematical features of the framework include a sizable number of elements and parameters making the model underconstrained. Moreover, a useful formal system should suggest testable hypotheses to further advance the investigated field. These perspectives are considered herein.

### Biological conjectures and limitations

The simulations discussed in this investigation represent archetypal patterns of drug-seeking including transitions from recreational to heavy use, and rehabilitation. They express the comorbidity between addiction and mood disorders for an addict vulnerable to both reward and antireward system neuroadaptations. As drug intake proceeds, the model estimates a steady decrease in the addict’s mood that increasingly oscillates until the virtual subject reaches the satiety rate of drug consumption. This computational model also suggests a possible remission of the individual’s healthy state as a consequence of cognitive adjustments induced by conventional or alternative treatments. The model predicts a contraction in the addict’s negative mood tendency and fluctuations while solely reward system neuroadaptations influence the hedonic valence of the individual. This rigidity endures during healing interventions as the model predicts stabilization of the subject’s mood, rather than its increase. When the unique source of neuroadaptations affecting the brain’s reward system relies on the antireward system, the model predicts a noteworthy negative deflection of the subject’s mood during the first number of drug intakes. The model also predicts that neuroadaptations occurring during healing periods, and that are uniquely induced by the antireward system, empower the individual with the possibility to regain a healthy mood state. These simulations also suggest that the satiety rate of drug consumption is reached more rapidly when the individual expresses only antireward system adaptations.

An important biological limitation of this model resides in the omission of mechanisms responsible for the increase of pharmacodynamic tolerance, which arises when receptors or second messengers are blunted by drugs such as alcohol or opiates (50). One approach to overcome this shortcoming can be found in the expansion and incorporation of a cellular and molecular scale within the model. Such elaboration could also enhance the computational framework with a greater descriptive ability for diverse classes of drugs. For nicotine dependence, a suitable candidate lies in a previously presented KR model which describes how dopaminergic signaling in the VTA increases through nicotine intake and influences synaptic plasticity in the dorsal striatum, making cigarette-smoking compulsive (51). A complementary candidate resides in a model describing how variations of extracellular levels of dopamine and glutamate within the brain’s reward system impact the virtual subject’s likelihood of drug consumption (52).

The discussed model could be further elaborated to consider alcohol dependence and treatment (53); to incorporate elements related to medical conditions that occur frequently together with drug abuse, such as posttraumatic stress disorder, attention deficit hyperactivity disorder, and schizophrenia (54); and to include components of genetic regulatory networks pertinent to addiction (55).

### The model’s high-dimensionality

A KR model is inclined toward a high-dimensionality due to a large number of descriptive variables since its objective is to describe the studied phenomenon as comprehensively as possible. The predictions presented in this investigation rely on the high-dimensional dynamical model shown in Figure 1 that comprises 21 time-dependent biological processes translated into the same number of mathematical expressions. In addition, there are 71 parameters, the setting of which can dramatically affect the model’s behavior. Such high-dimensionality could simulate an assortment of dynamics larger than the ones expressed by living creatures, consequently limiting the model’s predictive power. The natural processes included in the presented computational framework are sizable, yet their descriptions are determined conservatively. For instance, the processes comprising the neuropsychological scale of the model have limited domains of definition which facilitate their tractability. These restricted domains reflect biological plausibility and ease the sensitivity analysis (56), a necessary step toward the qualitative validation of the model.

Another attempt at mathematical moderation resides in the definition of healing interventions. The same mathematical definition used with different calibrations to mimic conventional and alternative cures was intentionally deployed as a lower-bound estimation of real-life cleansing episodes. In fact, the minimal duration of nicotine replacement therapies ranges from 3 weeks to 3 months (44), and mindfulness meditation requires 4 weeks of training to effectively influence regions surrounding the ACC of humans (57). Even though broadly delineated and similarly defined, these healing emulations provisionally advocate that for some nicotine addicts short interventions closely spaced in time (e.g., meditation episodes) have a more beneficial health impact on the brain’s cognitive substrate than longer interventions (e.g., nicotine patches). This conjecture is supported by a recent translational study where memories related to drug consumptions are triggered at different times to facilitate their extinction and decrease heroin craving in recovering humans (58, 59). The simulated healing processes also corroborate a recent cohort study debating how nicotine replacement therapies may not be the universal solution to attain long-term smoking abstinence (60).

### Implications for treatments

This article provides formal arguments, conditional upon the validity of the hypotheses defining the computational framework, in favor of a stronger consideration of the addict’s cognitive state evolution throughout the treatment process. In particular, this investigation suggests that higher rates of rehabilitation from drug addiction in humans can be reached by combining medical therapies that employ pharmaceutical drugs and counseling along with non-conventional treatments.

Several pharmacotherapies are available for smoking cessation, as for example nicotine in various forms (gums, patches, inhalers, tablets) and antidepressant drugs (44). Clinical studies show that these replacement therapies enhance the likelihood of rehabilitation by restraining drug craving during abstinence. The escalation of nicotine craving during smoking cessation is lower for therapies involving the use of two medications rather than for monotherapies, where the former results in a higher cessation rate, respectively of 54 and 45% (61). Non-pharmacological interventions included in the therapy, such as behavioral counseling and personal support, aim to further increase smoking cessation rates and are recognized as primary components for the therapy’s success (62). Behavioral counseling positively impacts cessation rates (63, 64) by providing patients with coping skills effective in the reduction of withdrawal symptoms (65), but is less significant in preventing relapse (66). With respect to rehabilitation, the combination of pharmaceutical drugs is not effective on all occasions. For smokers with low dependence on nicotine and living in antagonistic social environments (e.g., with a smoking partner) there is no significant difference in success rates of therapies involving one or the combination of two medications (65).

Behavioral counseling and personal support are instances of behavioral-cognitive therapies: non-pharmacological interventions which have flourished since the 1960s for the treatment of depression and anxiety (67–69). A complementary category of non-pharmacological interventions resides in mind-body practices, which include mindfulness meditation, guided imagery, and relaxation (70). Both the definitions of behavioral-cognitive (69) and mind-body (70) practices rely on the beneficial impact that healthy cognitive states exert on the overall person’s well being. A patient undergoing behavioral-cognitive therapy learns how to recognize and manage real-life situations that are negatively evaluated because of cognitive distortions, whereas mind-body practices provide the patient with a more realistic awareness that decreases irrational thoughts. In both cases the objective is to lead the patient to healthier physical and psychological states.

Behavioral-cognitive practices have demonstrated their positive impact on smoking cessation (66, 71), and mind-body techniques related to the treatment of nicotine addiction are restrained by a shortfall of related investigations (72), even though recent studies demonstrate their great potential. Preliminary experimental support in favor of mindfulness meditation as a practice decreasing relapse rates for post-rehabilitation patients was provided in a study including 168 participants who ceased the use of substances including alcohol, cocaine, and methamphetamines (73).

The rational speculation that arises while considering pharmacological and non-pharmacological healing practices suggests that current therapies deploying one or multiple pharmacological means along with counseling will raise their success rates by uniting with alternative medical practices. The computational framework presented in this investigation provides formal arguments to endorse this conjecture as the allostatic state of an addict, assessed through mood variations, is shown to improve because of cognitive interventions provided by practices comparable to those of conventional and alternative medicine.

If the predictions delivered by the computational model discussed in this investigation constitute a fair approximation to describe how cigarette-smoking influences the allostatic state of a human addicted to nicotine, then it is expected that an integrative medicine approach to drug rehabilitation will provide higher cessation rates and lower relapse rates than current therapies. Given that the allostatic theory of addiction is not limited to the description of a particular substance of abuse, this prediction should apply by extension also to addictive substances including heroin, alcohol, and others.

### Concluding remarks

The computational model presented in this article considers drug addiction as a disease (74). Such viewpoint may lead to “*increasing alienation, stigmatization, and social distance*” (75) of human beings abusing drugs. Multi-leveled overlooks of addiction that include biological/psychological/social (76), and even spiritual (77) elements are suggested as potential candidates to restrain such an undesirable possibility (78). Along these lines, the multiscale standpoint of the framework shown in Figure 1 aims to promote a comprehensive understanding of addiction that provides prospect for recovery, which seems to occur more often than commonly believed (79). The present investigation develops a multiscale computational model to further explore the allostatic theory of addiction (2) in terms of a KR model (48) in alignment with the exploratory review in Ref. (80), and aims to engage hypothesis-driven research (49) for addiction and allostasis. Such an approach can facilitate the detection of ambiguous knowledge that requires future biological and computational exploration in order to better understand this disease. The framework presented in this article supports the view that integrative medicine can be an effective approach to improve treatment of drug addiction.

## Conflict of Interest Statement

The authors declare that the research was conducted in the absence of any commercial or financial relationships that could be construed as a potential conflict of interest.

## Supplementary Material

The Supplementary Material for this article can be found online at http://www.frontiersin.org/Journal/10.3389/fpsyt.2013.00167/abstract
