# Supplementary material for: A Computational Hypothesis for Allostasis: Delineation of Substance Dependence, Conventional Therapies, and Alternative Treatments
Source: Front Psychiatry. 2013 Dec 19;4:167. doi: 10.3389/fpsyt.2013.00167 (PMC3868344; doi:10.3389/fpsyt.2013.00167)
Supplement: Supplementary file 1 [file 58424_Levy_Presentation1.PDF]

Supplementary Materials:

1. Materials and Methods
2. Table S1
3. Figures S1-S34

Title: A computational hypothesis for allostasis: delineation of substance dependence, conventional therapies, and alternative treatments

Authors: Yariv Z. Levy[1*], Dino J. Levy[2,3], Andrew G. Barto[1,5], Jerrold S. Meyer[4,5]

Affiliations:

[1]School of Computer Science, University of Massachusetts Amherst, Amherst, MA 01003.

[2]Recanati Faculty of Management, Tel-Aviv University, Tel-Aviv, Israel.

[3]Sagol School of Neuroscience, Tel-Aviv University, Tel-Aviv, Israel.

[4]Department of Psychology, University of Massachusetts Amherst, Amherst, MA 01003.

[5]Neuroscience and Behavior Program, University of Massachusetts Amherst, Amherst, MA 01003.

*Correspondence to: ylevy@cs.umass.edu

1. Materials and Methods

Herein are reported the definitions of the computational framework in Figure 1.

1.1 Notations

Different time scales are used: t represents time in minutes, and t^* represents time in hours.

When used as a conditional term, the sum $\sum_{s=0}^{t} Z(s)$ is abbreviated with $\sum Z$ and denotes the total number of drug intakes since the beginning of the simulation.

The inequality $t \geq t_{GO}$ indicates that the current time t is equal or grater than the time of the first drug intake t_{GO}. The presented simulations use t_{GO} = 5 [day].

The bounding function σ is defined as $\sigma(x) = \begin{cases} 0 & \text{if } x < 0 \\ x & \text{if } x \in [0,1] \\ 1 & \text{if } x > 1 \end{cases}$.

The parameter v, with $v \in$ [-0.05, 0.05], denotes the uniform noise that is different for every process and changes at each time step.

1.2 Expanded PK/PD

(Equation S1) Mood - M

$$M(t) = rc(t) + cd(t),$$

where

$rc(t)$ is the rush/comedown effect of the drug, defined below, and
$cd(t)$ is the cognitive dissonance, defined below.

(Equation S2) Rush/comedown effect of the drug - rc

$$rc(t) = \sum_{s=0}^{t} Z(s) \cdot \left(\alpha - \beta \cdot \left(\frac{t-s}{\Delta} \right)^2 \cdot e^{-\frac{1}{2}\left(\frac{t-s}{\Delta}\right)^2} \right),$$

where

$Z(t)$ is the occurrence of drug intakes, defined below, and
α, β, and Δ are constants $\in \mathbb{R}$ (e.g., $\alpha = 40$, $\beta = 60$, and $\Delta = 10$).

(Equation S3) Cognitive distortion - cd

$$cd(t+1) = \begin{cases} -T(t) + \gamma_M \cdot \Delta TSO(t^*) - \Delta TSO(t^* - 1) & \text{if } \sum Z \geq 1 \\ 0 & \text{otherwise,} \end{cases}$$

where

γ_M is a constant $\in \mathbb{R}$ (e.g., $\gamma_M = 0.3$),
$T(t)$ is the lowering effect on reward threshold, defined below,
$\Delta TSO(t^*) = T_S(t^*) - T_0(t^*)$, where $T_S(t^*)$ is the reward set point, and $T_0(t^*)$ is the baseline reward threshold, both defined below, and
$\sum Z$ is defined above.

(Equation S4) Drug intakes - Z

$$Z(t) = \begin{cases} 1 & \text{if } T(t) - T_S(t^*) > 0 \text{ and } \Delta_Z \geq a \text{ and } t \geq t_{GO} \\ 0 & \text{otherwise,} \end{cases}$$

where

$T(t)$ is the lowering effect on reward threshold, defined below,
$T_S(t^*)$ is the reward set point, defined below,
Δ_Z represents the number of minutes elapsed since the last drug intake, and
α is a constant $\in \mathbb{N}$ (e.g., $\alpha = 30$ [minute]).

(Equation S5) Lowering effect on reward threshold - T

$$T(t) = T_0(t^*) - \frac{T_{\max} \cdot C(t)}{T_{50} + C(t)},$$

where

$T_0(t^*)$ is the baseline reward threshold, defined below,
T_{max} is the maximum effect of the drug (e.g., $T_{max} = 120$),
T_{50} is the index of drug potency (e.g., $T_{50} = 588.6$ [nM]), and
$C(t)$ is the drug concentration in the brain, defined below.

(Equation S6) Drug concentration in the brain - C

$$C(t) = D \cdot \frac{k_{12}}{V_b(\alpha - \beta)} \cdot \sum_{s=0}^{t} Z(s) \cdot \left(e^{-\beta\cdot(t-s)} - e^{-\alpha\cdot(t-s)}\right),$$

where

D is the drug unit dose (e.g., $D = 250$ [μg]),
k_{12} is the compartment rate constant (e.g., $k_{12} = 0.0054$),
V_b is the apparent volume of distribution in the brain (e.g, $V_b = 1.67$ [45]),
α and β are the aggregate rate constants as discussed in [8], and
$Z(t)$ is the occurrence of drug intakes, defined above.

(Equation S7) Reward set point - T_S

$$T_S(t^*+1) = \begin{cases} \lambda \cdot \left(1 - e^{-\beta\cdot d}\right) + T_S(t_c) & \text{if } G(t^*) \geq 0 \text{ and } \sum Z \geq 1 \\ T_S(t_c) \cdot e^{-\gamma\cdot d} & \text{if } G(t^*) < 0 \text{ and } \sum Z \geq 1 \\ T_S(t^*) & \text{otherwise,} \end{cases}$$

where

$T_S(0)$ is a constant (e.g. $T_S(0) = 75$),
β, γ, and λ are constants $\in \mathbb{R}^+$ (e.g., $\beta = 0.05$, $\gamma = 0.05$, $\lambda = 100$),
d is a time-steps counter, reset to 0 when the sign of $G(t^*)$ changes,
t_c is the time t^* of last change of sign of $G(t^*)$,
$G(t^*)$ is the tendency of drug-seeking behavior, defined below, and
$\sum Z$ is defined above.

(Equation S8) Baseline reward threshold - T_0

$$T_0(t^*+1) = \begin{cases} T_0(t^*) + \delta_{T0} \cdot \left(-2 \cdot H(t^*) + 1\right) \cdot \left(\omega_S(t^*) - \omega_P(t^*) + \omega_D(t^*)\right) & \text{if } \sum Z \geq \alpha \\ T_0(t^*) & \text{otherwise,} \end{cases}$$

where

$T_0(0)$ is a constant (e.g. $T_0(0) = 100$),
δ_{T0} is a constant $\in \mathbb{R}^+$ (e.g., $\delta_{T0} = 0.03$),
$H(t^*)$ is the healing intervention process, defined below,
$\omega_S(t^*)$, $\omega_P(t^*)$, and $\omega_D(t^*)$ are the cognitive time-dependent weights, defined below,
α is a constant $\in \mathbb{N}^+$ (e.g., $\alpha = 20$ [intakes]); similar to stage 4 in [47], and
$\sum Z$ is defined above.

1.3 Cognitive scale

(Equation S9) Rationality density - rd

$$rd(t^*) = -\omega_S(t^*)\cdot S(t^*) + \omega_P(t^*)\cdot P(t^*) - \omega_D(t^*)\cdot D(t^*) - \\ -\omega_Q \cdot AQ(t^*) + \omega_A \cdot \left[AS(t^*) + AP(t^*) + AD(t^*)\right] + \omega_H \cdot H(t^*),$$

where

$\omega_S(t^*)$, $\omega_P(t^*)$, and $\omega_D(t^*)$ are the cognitive time-dependent weights, defined below,
$S(t^*)$, $P(t^*)$, and $D(t^*)$ are the internal processes, defined below,
$A_S(t^*)$, $A_P(t^*)$, $A_D(t^*)$, and $A_Q(t^*)$ are the external processes, defined below, and
$H(t^*)$ is the healing intervention process, defined below.

(Equation S10) Cognitive weights - ω_S, ω_P, ω_D, ω_Q, ω_A, ω_H

• For $i \in$ {Q, A, H}:

$$\omega = c$$

where

c is a constant $\in \mathbb{R}^+$ (e.g., $\omega_Q = 0.28$, $\omega_A = 0.35$, $\omega_H = 0.8$).

• For $i \in$ {S, P, D}:

$$\omega_i(t^*) = \max\left(\alpha_i(t^*-1) + \vartheta_i \cdot H(t^*), 0\right),$$

with

$$\alpha_i(t^*+1) = \begin{cases} \alpha_i(t^*) + \vartheta_i & \text{if } A \\ \alpha_i(t^*) - \eta_i & \text{if } B \\ \alpha_i(t^*) + \vartheta_i - \eta_i & \text{if } A \text{ and } B \\ \alpha_i(t^*) & \text{otherwise,} \end{cases}$$

where

$\alpha_i(0)$ is a constant (e.g., $\alpha_S(0) = 0.7$, $\alpha_P(0) = 1.2$, $\alpha_D(0) = 1$),
Conditions A and B are conditional terms defined as:

A : if $H(\Theta(t^*)) = 1$ and $p_A(t^*) < P(H\eta i)$,
[for some probability, and if H is active, and at last time-step of activation]
where $\Theta(t^*)$ is defined below

B : if $\sum_{s=t^*-1}^{t^*} Z(s) > 0$ and $p_B(t^*) < P(Z\eta i)$ and $\sum Z \le \beta$,
[for the first number of drug intakes, and for a certain probability, and if in the past hour there was at least one drug intake]
where β is a constant $\in \mathbb{N}^+$ (e.g., $\beta = 15$ [intakes]), similar to stage 3 in [47]

$p_A(t^*)$ and $p_B(t^*)$ are values sampled from a standard uniform distribution at each time-step t^*,
$P(H\eta i)$ and $P(Z\eta i)$ are constants $\in [0,1]$ which denote, respectively, the probabilities of permanent changes in cognitive weights $\omega_S(t^*)$, $\omega_P(t^*)$, and $\omega_D(t^*)$ after a healing intervention or after a drug intake, with

(e.g.)	S	P	D
$P(H\eta i)$	$P(H\eta S) = 0.7$	$P(H\eta P) = 0.9$	$P(H\eta D) = 0.6$
$P(Z\eta i)$	$P(Z\eta S) = 0.2$	$P(Z\eta P) = 0.1$	$P(Z\eta D) = 0.05$

$H(t^*)$ is the healing intervention process, defined below, and
ϑ_i and η_i are constants in the following domains:

	S	P	D
ϑ_i	$\in \mathbb{R}^-$ (e.g., $\vartheta_S = -0.26$)	$\in \mathbb{R}^+$ (e.g., $\vartheta_P = -0.42$)	$\in \mathbb{R}^-$ (e.g., $\vartheta_S = -0.32$)
η_i	$\in \mathbb{R}^-$ (e.g., $\eta_S = -0.1$)	$\in \mathbb{R}^+$ (e.g., $\eta_S = 0.05$)	$\in \mathbb{R}^-$ (e.g., $\eta_S = -0.15$)

(Equation S11) Cognitive state - cs

$$cs(t^*) = \frac{1}{2}\tanh\left(\alpha \cdot cs(t^*-1) + \beta \cdot rd(t^*) + \gamma\right) + \frac{1}{2}$$

where

α and β are constants $\in \mathbb{R}^+$ (e.g., $\alpha = 0.25$, $\beta = 0.25$)
γ is a constant $\in \mathbb{R}$ (e.g., $\gamma = -0.4052$); this constant can also be computed using the following equation, as described in [56]:

$$\gamma = \frac{1}{2}\left[\alpha - \beta \cdot \left(\omega_S(0) - \omega_P(0) + \omega_D(0) + \omega_Q + \omega_A\right)\right], \text{ where}$$

$\omega_S(0)$, $\omega_P(0)$, and $\omega_D(0)$ a are the values of the cognitive time-dependent weights at time $t^* = 0$,
α and β are the same constants used for $cs(t^*)$.

<u>1.4 Healing scale</u>

(Equation S12) Healing intervention - H

$$H(t^*) = \begin{cases} 1 & \text{if } p(t^*) < P(nr) \text{ or if } d \in \left[1, \Theta(t^*)\right] \\ 0 & \text{otherwise,} \end{cases}$$

where

$P(nr)$ is the probability of an healing intervention, $P(nr) \in [0,1]$; this probability can be based on data as in [12],
$p(t^*)$ is a value sampled from a standard uniform distribution at each time-step t^*; in the simulations presented in this article, H processes are triggered at specific times,
d is a time step counter reset at every instance of H, and
$\Theta(t^*)$ is the activation time of H, which increases for consecutive instances:

$$\Theta(t^*) = \begin{cases} \Theta(t^*) + \delta_i & \text{if } p(t^*) < P(nr) \\ \Theta(t^*-1) & \text{if } d \in \left[1, \Theta(t^*-1)\right] \\ \max\left(0,\ \Theta(t^*) - \delta_d\right) & \text{otherwise,} \end{cases}$$

where

δ_i and δ_d are constants $\in \mathbb{N}^+$, (e.g., $\delta_i = 15$, $\delta_d = 1$ [hour]).

1.5 Neuropsychological scale

The internal processes are *S*, *P*, *D*, and *Q*. The external triggers are *AS*, *AP*, *AD*, and *AQ*.

(Equation S13) Stress - S

$$S(t^*) = \begin{cases} \sigma\left(1-\left(1-S_0\right)\cdot e^{-\beta\cdot d}+\nu\right) & \text{if } G(t^*)>0 \\ \sigma\left(S(t^*-1)+\nu\right) & \text{if } G(t^*)=0 \\ \sigma\left(S_0\cdot e^{-\gamma\cdot d}+\nu\right) & \text{if } G(t^*)<0, \end{cases}$$

where

S_0 is the value $S(t_c)$, where t_c is the time t^* of last change of sign of $G(t^*)$,
β and γ are constants $\in \mathbb{R}^+$ (e.g., $\beta = 0.002$, $\gamma = 0.002$),
d is a time-steps counter, reset to 0 when the sign of $G(t^*)$ changes,
$G(t^*)$ is the tendency of drug-seeking behavior, defined below, and
ν and $\sigma(x)$ are defined above.

(Equation S14) Pain - P

$$P(t^*) = \begin{cases} \sigma\left(P_0\cdot e^{-\beta\cdot d}+\nu\right) & \text{if } G(t^*)>0 \\ \sigma\left(P(t^*-1)+\nu\right) & \text{if } G(t^*)=0 \\ \sigma\left(1-(1-P_0)\cdot e^{-\gamma\cdot d}+\nu\right) & \text{if } G(t^*)<0, \end{cases}$$

where

P_0 is the value $P(t_c)$, where t_c is the time t^* of last change of sign of $G(t^*)$,
β and γ are constants $\in \mathbb{R}^+$ (e.g., $\beta = 0.0002$, $\gamma = 0.01$),
d is a time-steps counter, reset to 0 when the sign of $G(t^*)$ changes,
$G(t^*)$ is the tendency of drug-seeking behavior, defined below, and
ν and $\sigma(x)$ are defined above.

(Equation S15) Drug craving - D

$$D(t^*) = \begin{cases} \sigma\left(1-\left(1-D_0\right)\cdot e^{-\beta\cdot d}+\nu\right) & \text{if } G(t^*)>0 \text{ and } d\in[1,\tau] \\ \sigma\left(D'_0\cdot e^{-\beta\cdot d}+\nu\right) & \text{if } G(t^*)>0 \text{ and } d>\tau \\ \sigma\left(D(t^*-1)+\nu\right) & \text{if } G(t^*)=0 \\ \sigma\left(1-\left(1-D_0\right)\cdot e^{-\gamma\cdot d}+\nu\right) & \text{if } G(t^*)<0, \end{cases}$$

where

D_0 is the value $D(t_c)$, where t_c is the time t^* of last change of sign of $G(t^*)$,
D'_0 is the value $D(t_c+\tau)$, where t_c is the time t^* of last change of sign of $G(t^*)$, and τ is a constant $\in \mathbb{N}^+$ (e.g., $\tau = 20$ [hour]),
β and γ are constants $\in \mathbb{R}^+$ (e.g., $\beta = 0.00002$, $\gamma = 0.002$),
d is a time-steps counter, reset to 0 when the sign of $G(t^*)$ changes,
$G(t^*)$ is the tendency of drug-seeking behavior, defined below, and
ν and $\sigma(x)$ are defined above.

(Equation S16) Saliency to drug cues - Q

$$Q(t^*) = \begin{cases} \sigma\left(Q(t^*-1)+\nu\right) & \text{if } G(t^*)>0 \text{ and } d\in[1,\tau] \text{ or if } G(t^*)=0 \\ \sigma\left(Q'_0\cdot e^{-\beta\cdot d}+\nu\right) & \text{if } G(t^*)>0 \text{ and } d>\tau \\ \sigma\left(1-\left(1-Q_0\right)\cdot e^{-\gamma\cdot d}+\nu\right) & \text{if } G(t^*)<0, \end{cases}$$

where

Q_0 is the value $Q(t_c)$, where t_c is the time t^* of last change of sign of $G(t^*)$,
Q'_0 is the value $Q(t_c+\tau)$, where t_c is the time t^* of last change of sign of $G(t^*)$, and τ is a constant $\in \mathbb{N}^+$ (e.g., $\tau = 10$ [hour]),
β and γ are constants $\in \mathbb{R}^+$ (e.g., $\beta = 0.002$, $\gamma = 0.0005$),
d is a time-steps counter, reset to 0 when the sign of $G(t^*)$ changes,
$G(t^*)$ is the tendency of drug-seeking behavior, defined below, and
ν and $\sigma(x)$ are defined above.

(Equation S17) Acute shock - AS

$$AS(t^*) = \begin{cases} S_0 & \text{if } G(t^*)>0 \text{ and } p(t^*)<P(AS) \text{ or if } d\in[1,\tau_1] \\ \rho\cdot AS(t^*-1) & \text{if } d\in[\tau_1,\tau_2] \\ 0 & \text{otherwise,} \end{cases}$$

where

S_0 and ρ are constants $\in \mathbb{R}^+$ (e.g., $S_0 = 0.75$, $\rho = 0.9$),
$G(t^*)$ is the tendency of drug-seeking behavior, defined below,
$p(t^*)$ is a value sampled from a standard uniform distribution at each time-step t^*,
$P(AS)$ is the probability of an acute shock (e.g., $P(AS) = 0.01$),
d is a time-steps counter, reset to 0 when a new $AS(t^*)$ arises, and
τ_1 and τ_2 are constants $\in \mathbb{N}^+$ with $\tau_2 > \tau_1$ (e.g., $\tau_1 = 20$, $\tau_2 = 60$ [hour]).

(Equation S18) Acute trauma - AP

$$AP(t^*) = \begin{cases} P_0 & \text{if } G(t^*)<0 \text{ and } p(t^*)<P(AP) \text{ or if } d\in[1,\tau_1] \\ \rho\cdot AP(t^*-1) & \text{if } d\in[\tau_1,\tau_2] \\ 0 & \text{otherwise,} \end{cases}$$

where

P_0 and r are constants $\in \mathbb{R}^+$ (e.g., $P_0 = 0.45$, $r = 0.4$),
$G(t^*)$ is the tendency of drug-seeking behavior, defined below,
$p(t^*)$ is a value sampled from a standard uniform distribution at each time-step t^*,
$P(AP)$ is the probability of an acute shock (e.g., $P(AP) = 0.03$),
d is a time-steps counter, reset to 0 when a new $AP(t^*)$ arises, and
τ_1 and τ_2 are constants $\in \mathbb{N}^+$ with $\tau_2 > \tau_1$ (e.g., $\tau_1 = 15$, $\tau_2 = 50$ [hour]).

(Equation S19) Acute drug priming - AD

$$AD(t^*) = \begin{cases} D_0 & \text{if } G(t^*) > 0 \text{ and } p(t^*) < P(AD) \text{ or if } d \in [1, \tau_1] \\ \rho \cdot AD(t^* - 1) & \text{if } d \in [\tau_1, \tau_2] \\ 0 & \text{otherwise,} \end{cases}$$

where

D_0 and r are constants $\in \mathbb{R}^+$ (e.g., $D_0 = 0.75$, $r = 0.9$),
$G(t^*)$ is the tendency of drug-seeking behavior, defined below,
$p(t^*)$ is a value sampled from a standard uniform distribution at each time-step t^*,
$P(AD)$ is the probability of an acute shock (e.g., $P(AD) = 0.03$),
d is a time-steps counter, reset to 0 when a new $AD(t^*)$ arises, and
τ_1 and τ_2 are constants $\in \mathbb{N}^+$ with $\tau_2 > \tau_1$ (e.g., $\tau_1 = 5$, $\tau_2 = 30$ [hour]).

(Equation S20) Acute drug cue - AQ

$$AQ(t^*) = \begin{cases} Q(t^*) & \text{if } p(t^*) < P(AQ) \\ AQ(t^* - 1) & \text{if } d \in [1, \tau_1] \\ \rho \cdot AQ(t^* - 1) & \text{if } d \in [\tau_1, \tau_2] \\ 0 & \text{otherwise,} \end{cases}$$

where

$Q(t^*)$ is the saliency to drug cues, defined above,
ρ is a constants $\in \mathbb{R}^+$ (e.g., $\rho = 0.9$),
$p(t^*)$ is a value sampled from a standard uniform distribution at each time-step t^*,
$P(AQ)$ is the probability of an acute shock (e.g., $P(AQ) = 0.02$),
d is a time-steps counter, reset to 0 when a new $AQ(t^*)$ arises, and
τ_1 and τ_2 are constants $\in \mathbb{N}^+$ with $\tau_2 > \tau_1$ (e.g., $\tau_1 = 20$, $\tau_2 = 40$ [hour]).

1.6 Behavioral scale

(Equation S21) Tendency of drug-seeking behavior - G

$$G(t^*) = I \cdot cs(t^*) - C \cdot (1 - cs(t^*)),$$

where

I and C are a constant (e.g., $I = 1$, $C = 1$),
$cs(t^*)$ is the cognitive state, defined above.

2. SUPPLEMENTARY TABLE

Table S1: Values of the parameters as used in Figures 2-7 and Figures S1-S34.

Eq.	Parameter	Value
S1,S9	*N/A*	N/A
S2	α	40
	β	60
	Δ	10
S3	γ	0.3
S4	α	30
S5	T_{max}	120
	T_{50}	588.6
S6	Δ	250
	k_{12}	0.0054
	V_b	1.67
S7	$T_S(0)$	75
	β	0.05
	γ	0.05
	λ	100
S8	$T_0(0)$	100
	δ_{T0}	0.03
	α	20
S10	ω_O	0.28
	ω_A	0.35
	ω_H	0.8
	$\alpha_S(0)$	0.7
	$\alpha_P(0)$	1.2
	$\alpha_D(0)$	1
	$P(H\eta S)$	0.2, 0.4, 0.8
	$P(H\eta P)$	0.175, 0.35, 0.7
	$P(H\eta D)$	0.8, 0.7, 0.6
	$P(Z\eta S)$	0.05, 0.1, 0.2, 0.4, 0.8
	$P(Z\eta P)$	0.025, 0.05, 0.1, 0.2, 0.4
	$P(Z\eta D)$	0.0125, 0.025, 0.05, 0.1, 0.2
	υ_S	–0.26
	υ_P	0.42
	υ_D	–0.32
	η_S	–0.1
	η_P	0.05
	η_D	–0.15
S11	α	0.25
	β	0.85
	γ	–0.4052
S12	δ_i	15
	δ_d	1
S13	β	0.002
	γ	0.002

S14	β	0.0002
	γ	0.01
S15	τ	20
	β	0.00002
	γ	0.002
S16	τ	10
	β	0.002
	γ	0.0005
S17	S_0	0.75
	ρ	0.9
	$P(AS)$	0.01
	τ_1	20
	τ_2	60
S18	P_0	0.45
	ρ	0.4
	$P(AP)$	0.03
	τ_1	15
	τ_2	50
S19	D_0	0.65
	ρ	0.55
	$P(AD)$	0.03
	τ_1	5
	τ_2	30
S20	ρ	0.9
	$P(AQ)$	0.02
	τ_1	20
	τ_2	40
S21	I	1
	C	1

3. SUPPLEMENTARY FIGURES S1-S34

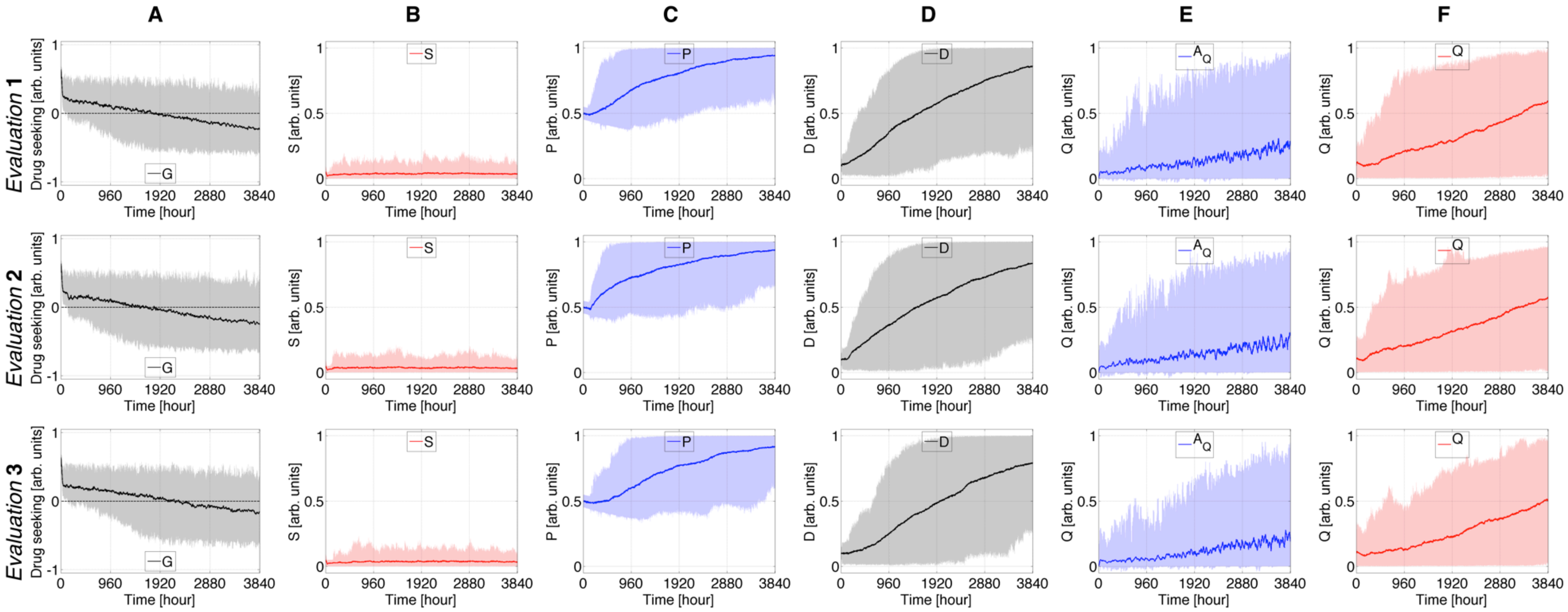

Figure S1: Details of Case Study 1 presented in Figures 2 and 3. Simulations of virtual behavior for cigarette consumption over a period of 160 days. Cigarettes are available on the fifth day. Results are the average of 100 runs. Evaluation 1 simulates both T_S and T_0 as time-dependent processes. T_S is constant and T_0 time-dependent in Evaluation 2, T_S is time-dependent and T_0 constant in Evaluation 3. Column A reports the evolution of drug-seeking behavior G; columns B-D the progression of internal processes stress S (red), pain P (blue), and drug craving D (black); columns E-F the external trigger acute drug cue AQ (blue) and the internal process of saliency to drug cues Q (red). The shades correspond to 95% simulation envelopes.

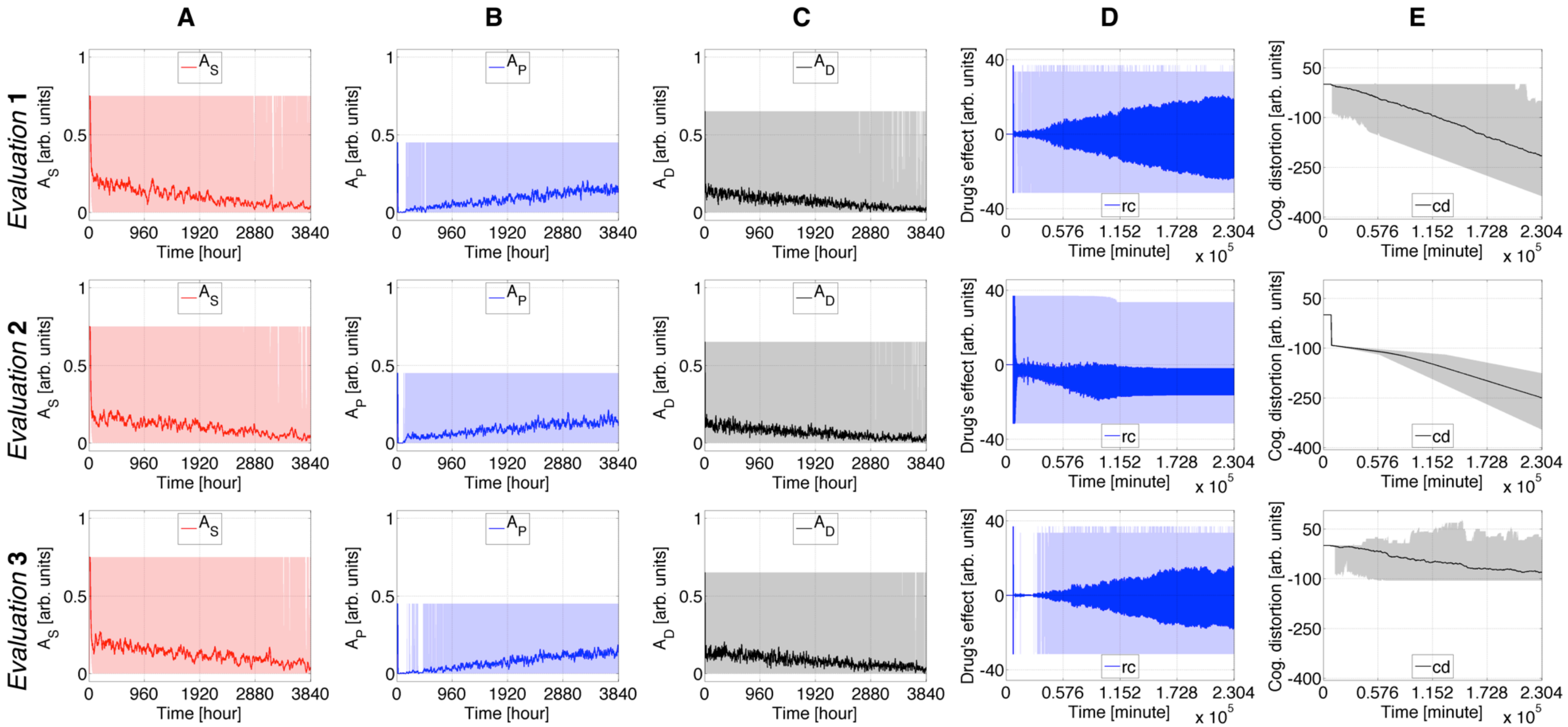

Figure S2: Details of Case Study 1 presented in Figures 2 and 3. Simulations of virtual behavior for cigarette consumption over a period of 160 days. Cigarettes are available on the fifth day. Results are the average of 100 runs. Evaluation 1 simulates both T_S and T_0 as time-dependent processes. T_S is constant and T_0 time-dependent in Evaluation 2, T_S is time-dependent and T_0 constant in Evaluation 3. Columns A-C report the external triggers of acute shock *AS* (red), acute trauma *AP* (blue), and acute drug priming *AD* (black); column D the rush/comedown effect *rc* of drug intakes; and column E the cognitive distortion *cd*. The shades correspond to 95% simulation envelopes. The abscissas correspond to the simulation's time-steps: hours in columns A-C, minutes in columns D and E.

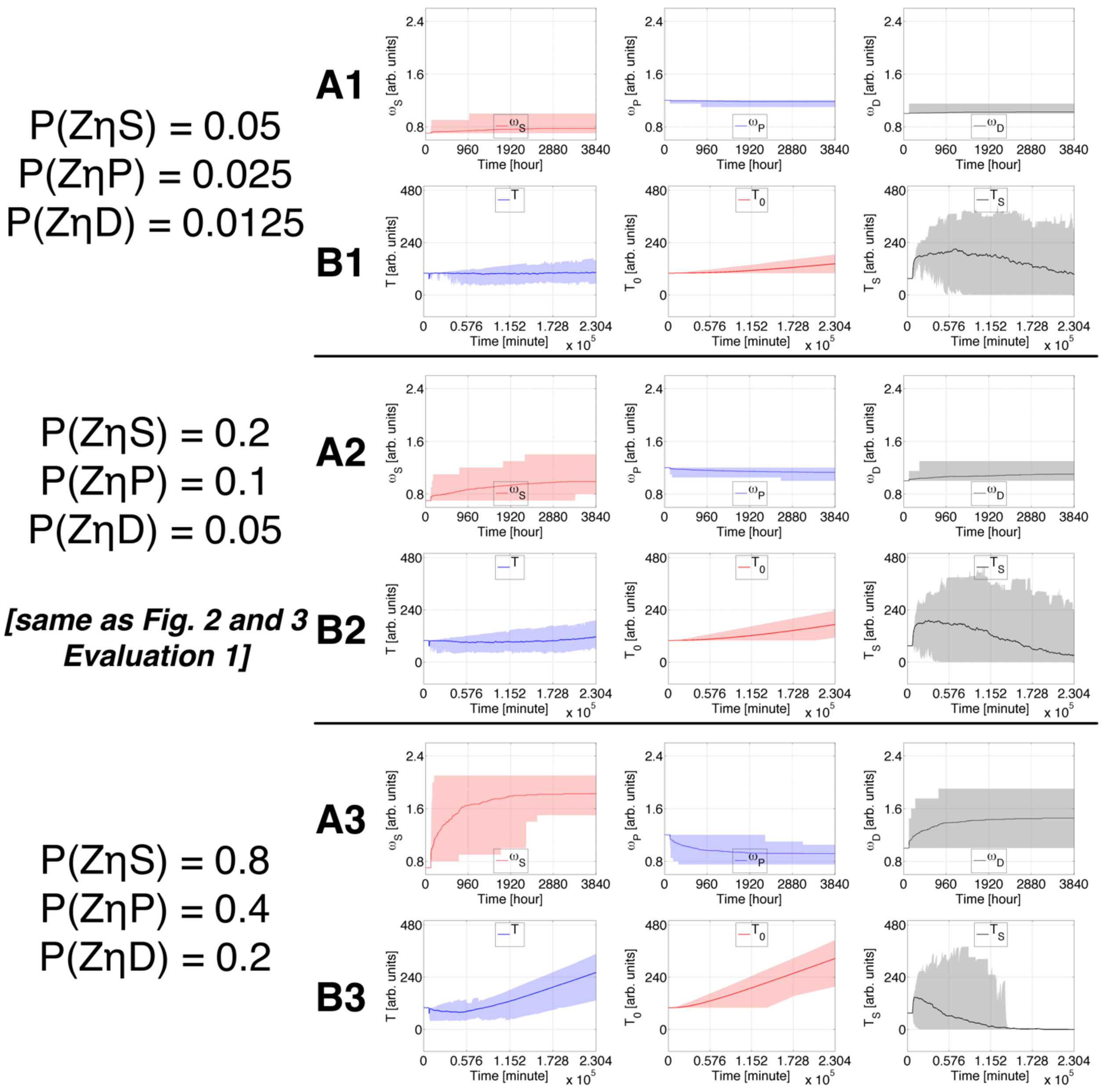

Figure S3: Comparison of different probabilities defining the associative learning between the drug and its pleasurable effect. Simulations of virtual behavior for cigarette consumption over a period of 160 days. Cigarettes are available on the fifth day. Results are the average of 100 runs. For the first number of consumed cigarettes, alterations of ω_S, ω_P, and ω_D facilitating maladaptive behavior occur with arbitrary probabilities. Three sets of probabilities $P(Z\eta i)$, with $i \in \{S, P, D\}$ are tested: [0.05, 0.025, 0.0125], [0.2, 0.1, 0.05], and [0.8, 0.4, 0.2]. Rows A1-A3 report the evolution of cognitive weights ω_S (red), ω_P (blue), and ω_D (black); rows B1-B3 the progression of T (blue), T_0 (red), and T_S (black); column C the virtual subject's mood; and column D the average number of drug intakes (red), and the abstinence index (blue) over time. The shades correspond to 95% simulation envelopes. The simulation time-scales are multiple: hours for rows A1-A3, and minutes for rows B1-B3.

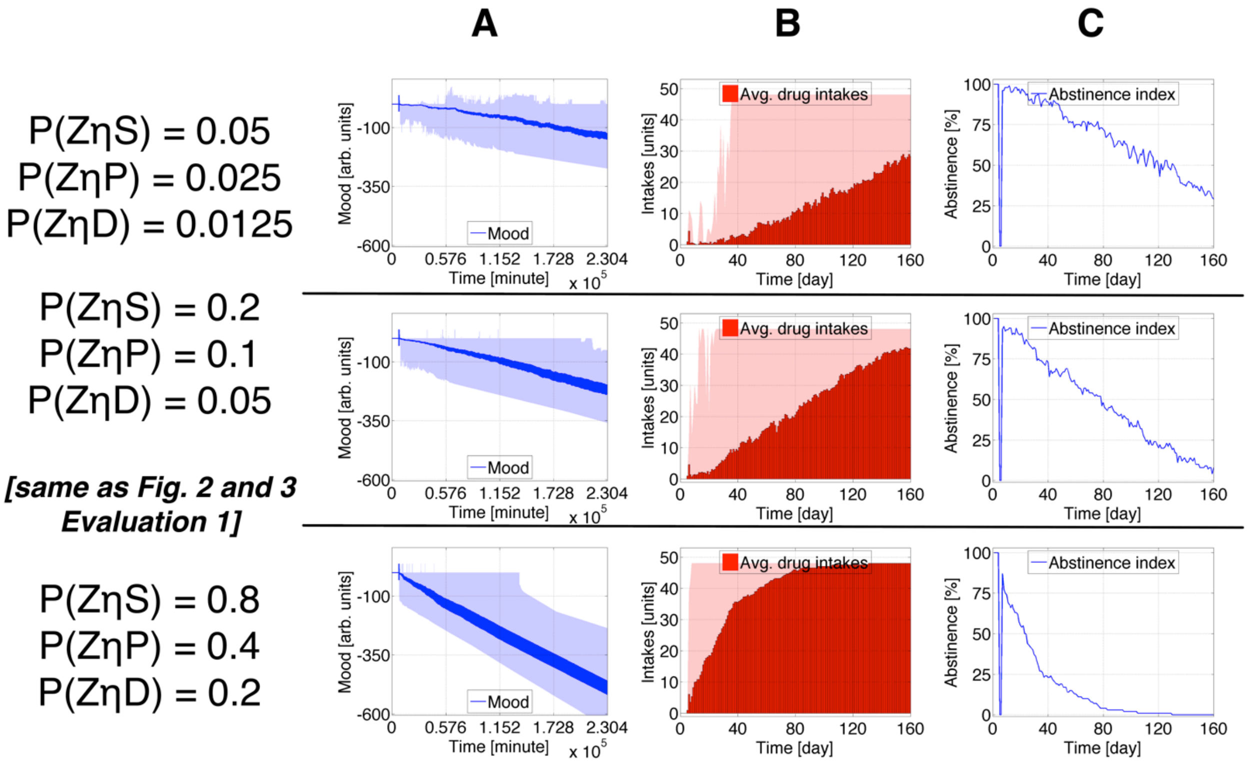

Figure S4: Comparison of different probabilities defining the associative learning between the drug and its pleasurable effect. Simulations of virtual behavior for cigarette consumption over a period of 160 days. Cigarettes are available on the fifth day. Results are the average of 100 runs. For the first number of consumed cigarettes, alterations of ω_S, ω_P, and ω_D facilitating maladaptive behavior occur with arbitrary probabilities. Three sets of probabilities $P(Z\eta i)$, with $i \in \{S, P, D\}$ are tested: [0.05, 0.025, 0.0125], [0.2, 0.1, 0.05], and [0.8, 0.4, 0.2]. Column A reports the virtual subject's mood; column B the average number of drug intakes, and column C the abstinence index over time. The shades correspond to 95% simulation envelopes. The simulation time-scales are multiple: minutes for column A, days columns B and C. Maladaptive behavior is facilitated for higher probabilities. Simulations using lower probabilities terminate with the virtual subject exhibiting an average consumption of ~30 intakes/day and a abstinence index of ~30%. Higher probabilities lead these predicted measures to ~45 intakes/day and ~2% abstinence. The downslope of M becomes stronger as the probabilities become larger.

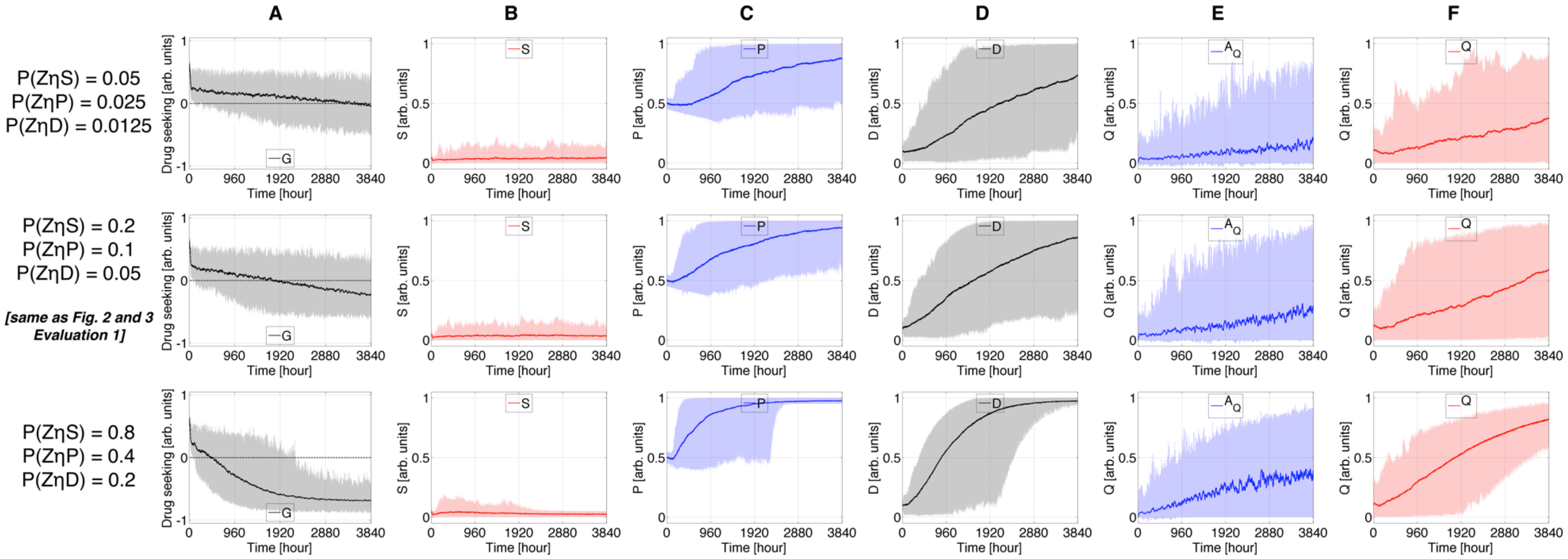

Figure S5: Details of simulations presented in Figures S3 and S4. Simulations of virtual behavior for cigarette consumption over a period of 160 days. Cigarettes are available on the fifth day. Results are the average of 100 runs. For the first number of consumed cigarettes, alterations of ω_S, ω_P, and ω_D facilitating maladaptive behavior occur with arbitrary probabilities. Three sets of probabilities $P(Z\eta i)$, with $i \in$ {S, P, D} are tested: [0.05, 0.025, 0.0125], [0.2, 0.1, 0.05], and [0.8, 0.4, 0.2]. Column A reports the evolution of drug-seeking behavior G; columns B-D the progression of internal processes stress S (red), pain P (blue), and drug craving D (black); columns E-F the external trigger acute drug cue AQ (blue) and the internal process of saliency to drug cues Q (red). The shades correspond to 95% simulation envelopes.

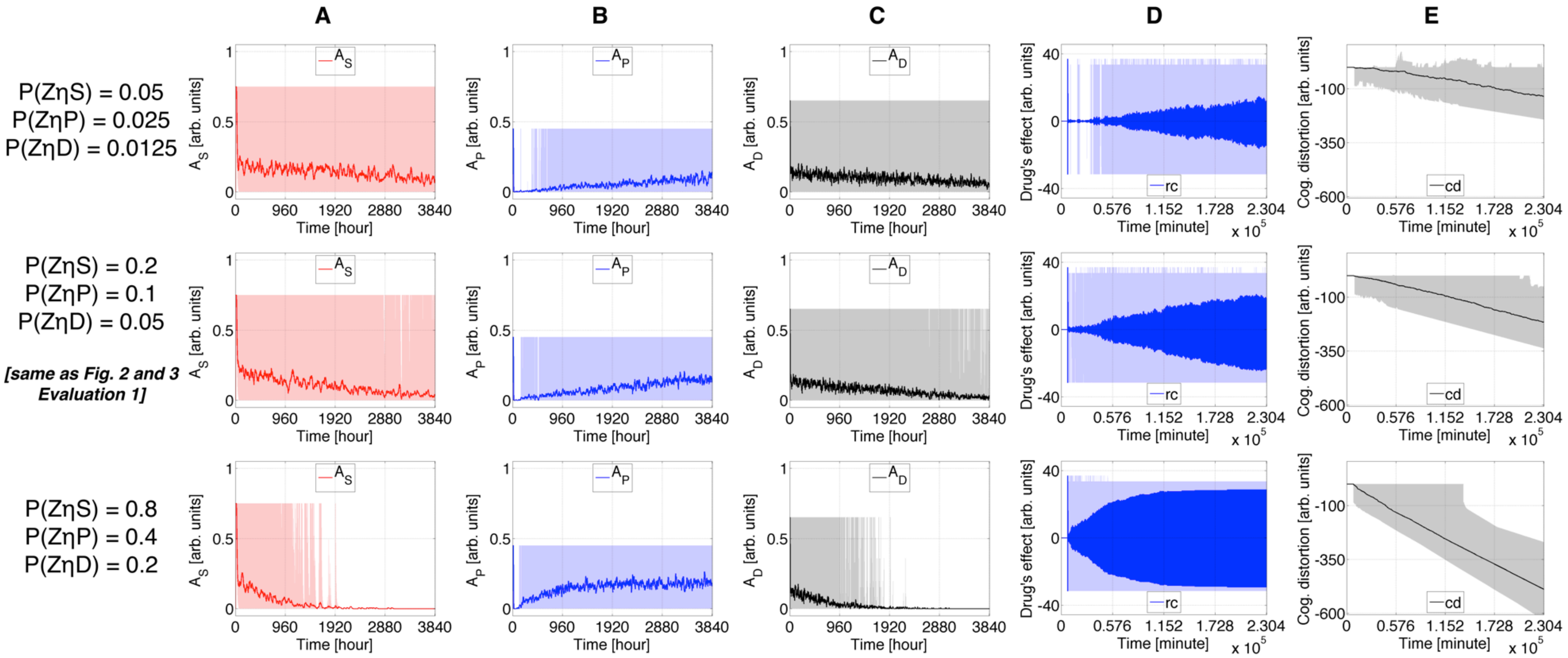

Figure S6: Details of simulations presented in Figures S3 and S4. Simulations of virtual behavior for cigarette consumption over a period of 160 days. Cigarettes are available on the fifth day. Results are the average of 100 runs. For the first number of consumed cigarettes, alterations of ω_S, ω_P, and ω_D facilitating maladaptive behavior occur with arbitrary probabilities. Three sets of probabilities $P(Z\eta i)$, with $i \in \{S, P, D\}$ are tested: [0.05, 0.025, 0.0125], [0.2, 0.1, 0.05], and [0.8, 0.4, 0.2]. Columns A-C report the external triggers of acute shock AS (red), acute trauma AP (blue), and acute drug priming AD (black); column D the rush/comedown effect rc of drug intakes; and column E the cognitive distortion cd. The shades correspond to 95% simulation envelopes. The abscissas correspond to the simulation's time-steps: hours in columns A-C, minutes in columns D and E.

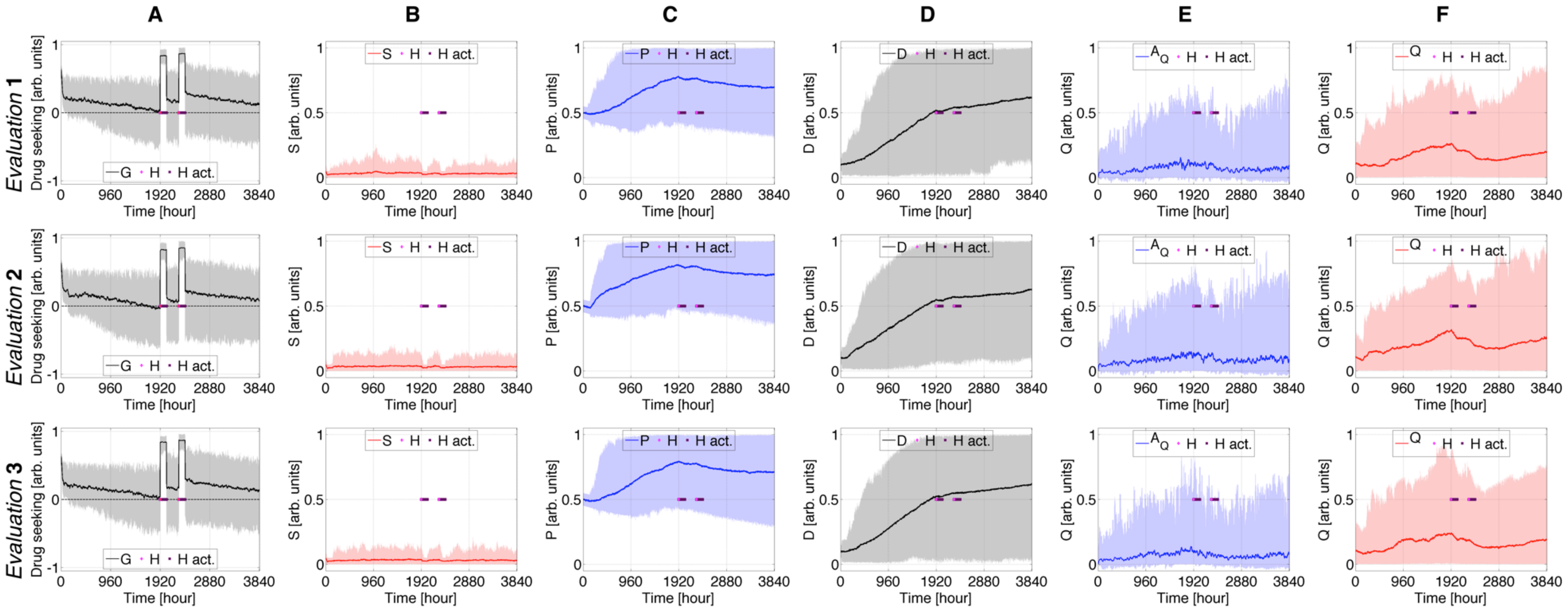

Figure S7: Details of Case Study 2 presented in Figures 4 and 5. Simulations of virtual behavior for cigarette consumption over a period of 160 days. Cigarettes are available on the fifth day. Results are the average of 100 runs. Evaluation 1 simulates both T_S and T_0 as time-dependent processes. T_S is constant and T_0 time-dependent in Evaluation 2, T_S is time-dependent and T_0 constant in Evaluation 3. In all Evaluations, the recovery process H is activated at $t = 1920$ and $t = 2280$ [hours] (in light pink) and stays active for 120 hours (dark pink). Column A reports the evolution of drug-seeking behavior G; columns B-D the progression of internal processes stress S (red), pain P (blue), and drug craving D (black); columns E-F the external trigger acute drug cue AQ (blue) and the internal process of saliency to drug cues Q (red). The shades correspond to 95% simulation envelopes.

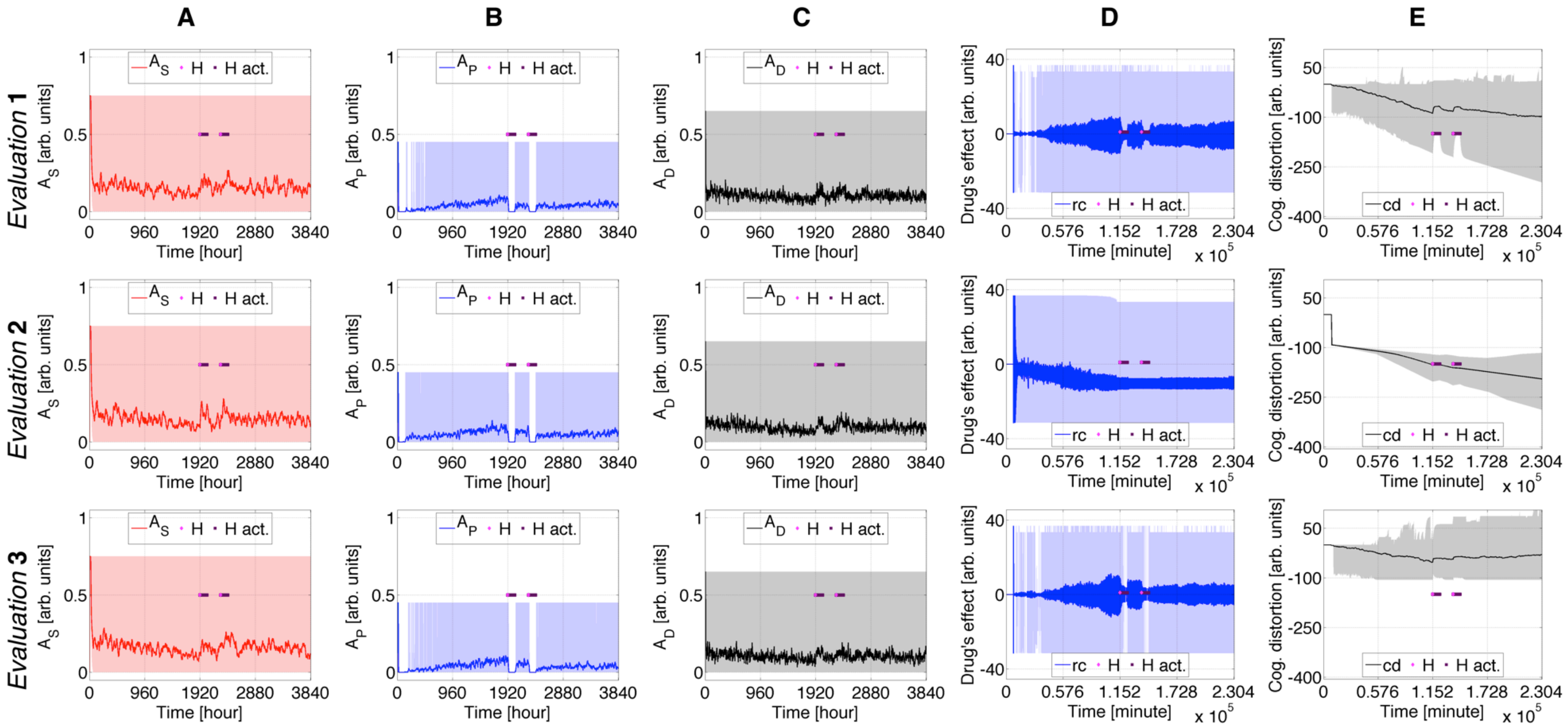

Figure S8: Details of Case Study 2 presented in Figures 4 and 5. Simulations of virtual behavior for cigarette consumption over a period of 160 days. Cigarettes are available on the fifth day. Results are the average of 100 runs. Evaluation 1 simulates both T_S and T_0 as time-dependent processes. T_S is constant and T_0 time-dependent in Evaluation 2, T_S is time-dependent and T_0 constant in Evaluation 3. In all Evaluations, the recovery process *H* is activated at $t = 1920$ and $t = 2280$ [hours] (in light pink) and stays active for 120 hours (dark pink). Columns A-C report the external triggers of acute shock *AS* (red), acute trauma *AP* (blue), and acute drug priming *AD* (black); column D the rush/comedown effect *rc* of drug intakes; and column E the cognitive distortion *cd*. The shades correspond to 95% simulation envelopes. The abscissas correspond to the simulation's time-steps: hours in columns A-C, minutes in columns D and E.

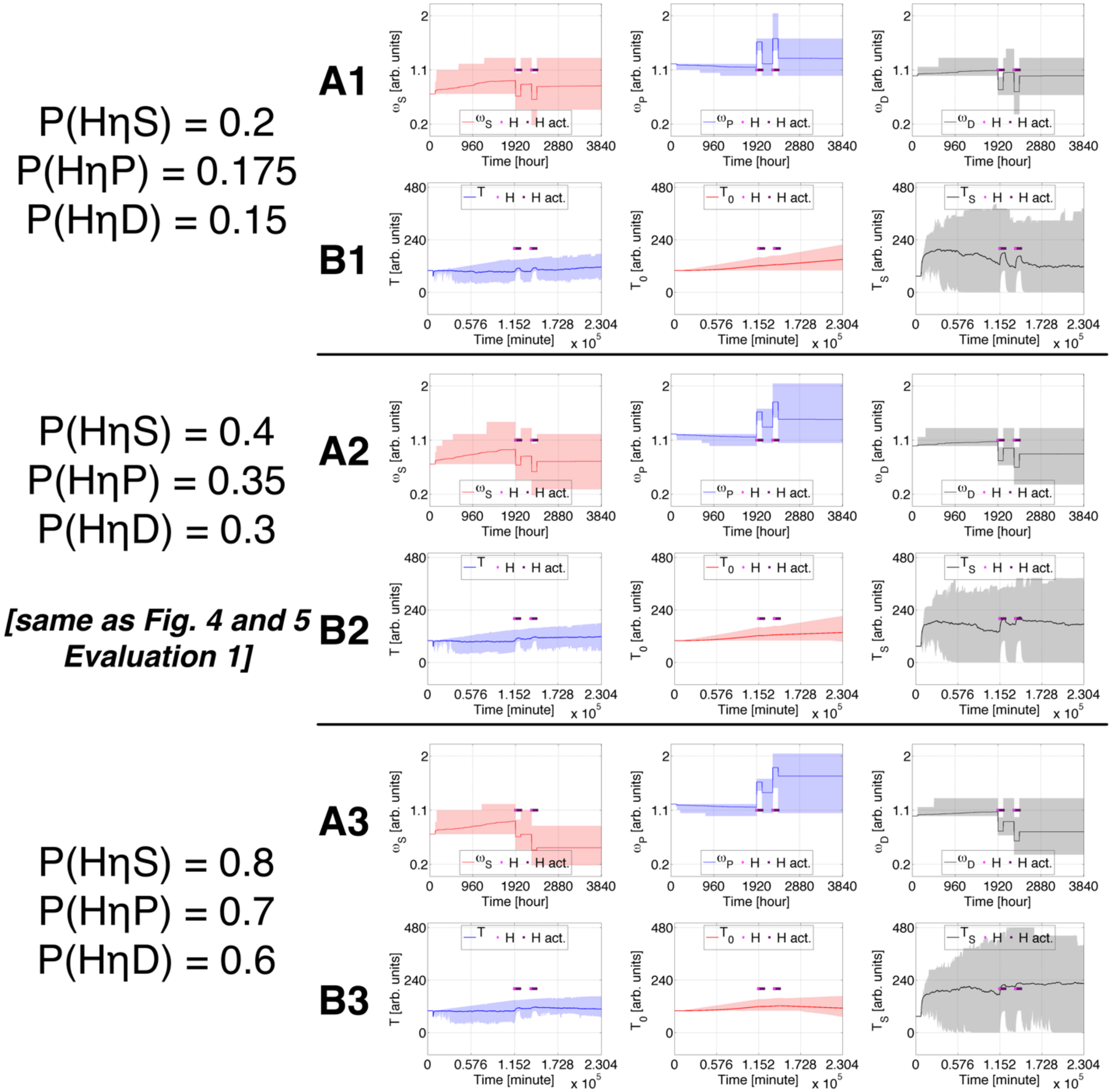

Figure S9: Comparison of different probabilities defining the durability of H for conventional therapies, with both T_S and T_0 time-dependent. Simulations of virtual behavior for cigarette consumption over a period of 160 days. Cigarettes are available on the fifth day. Results are the average of 100 runs. The recovery process H is activated at $t = 1920$ and $t = 2280$ [hours] (in light pink) and stays active for 120 hours (dark pink). At the end of each therapeutic session, the positive influence that H exerts on ω_S, ω_P, and ω_D become permanent or disappears, depending on arbitrary probabilities. Three sets of probabilities $P(H\eta i)$, with $i \in \{S, P, D\}$, are tested: [0.2, 0.175, 0.15], [0.4, 0.35, 0.3], and [0.8, 0.7, 0.6]. Rows A1-A3 report the evolution of cognitive weights ω_S (red), ω_P (blue), and ω_D (black); rows B1-B3 the progression of T (blue), T_0 (red), and T_S (black); column C the virtual subject's mood; and column D the average number of drug intakes (red), and the abstinence index (blue) over time. The shades correspond to 95% simulation envelopes. The simulation time-scales are multiple: hours for rows A1-A3, and minutes for rows B1-B3.

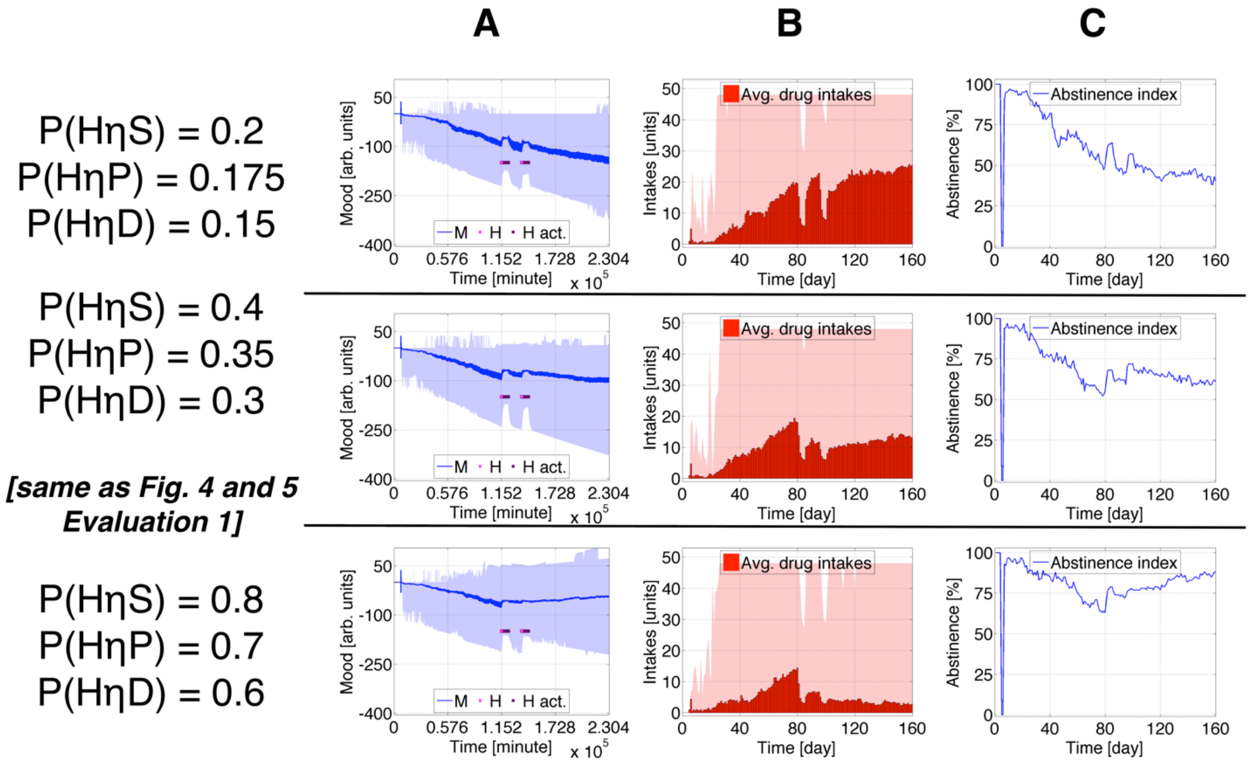

Figure S10: Comparison of different probabilities defining the durability of *H* for conventional therapies, with both T_S and T_0 time-dependent. Simulations of virtual behavior for cigarette consumption over a period of 160 days. Cigarettes are available on the fifth day. Results are the average of 100 runs. The recovery process *H* is activated at $t = 1920$ and $t = 2280$ [hours] (in light pink) and stays active for 120 hours (dark pink). At the end of each therapeutic session, the positive influence that *H* exerts on ω_S, ω_P, and ω_D become permanent or disappears, depending on arbitrary probabilities. Three sets of probabilities $P(H\eta i)$, with $i \in \{S, P, D\}$, are tested: [0.2, 0.175, 0.15], [0.4, 0.35, 0.3], and [0.8, 0.7, 0.6]. Column A reports the virtual subject's mood; column B the average number of drug intakes, and column C the abstinence index over time. The shades correspond to 95% simulation envelopes. The simulation time-scales are multiple: minutes for column A, days columns B and C. The trial employing the lowest probabilities terminates at advanced stage 4 (~25 intakes/day and ~41% abstinence), whereas the highest set of probabilities leads to stage 1 or intermediate stage 2 (~3.5 intakes/day and ~88% abstinence).

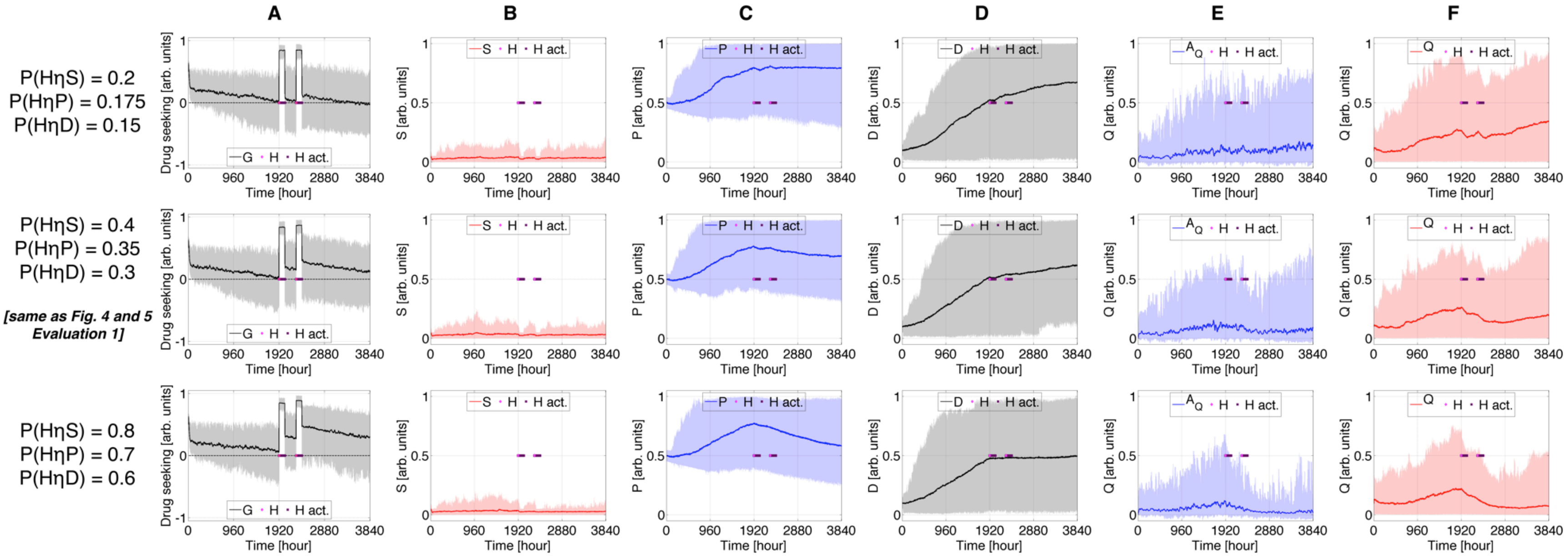

Figure S11: Details of simulations presented in Figures S9 and S10. Simulations of virtual behavior for cigarette consumption over a period of 160 days. Cigarettes are available on the fifth day. Results are the average of 100 runs. The recovery process H is activated at t = 1920 and t = 2280 [hours] (in light pink) and stays active for 120 hours (dark pink). At the end of each therapeutic session, the positive influence that H exerts on ω_S, ω_P, and ω_D become permanent or disappears, depending on arbitrary probabilities. Three sets of probabilities $P(H\eta i)$, with $i \in$ {S, P, D}, are tested: [0.2, 0.175, 0.15], [0.4, 0.35, 0.3], and [0.8, 0.7, 0.6]. Column A reports the evolution of drug-seeking behavior G; columns B-D the progression of internal processes stress S (red), pain P (blue), and drug craving D (black); columns E-F the external trigger acute drug cue AQ (blue) and the internal process of saliency to drug cues Q (red). The shades correspond to 95% simulation envelopes.

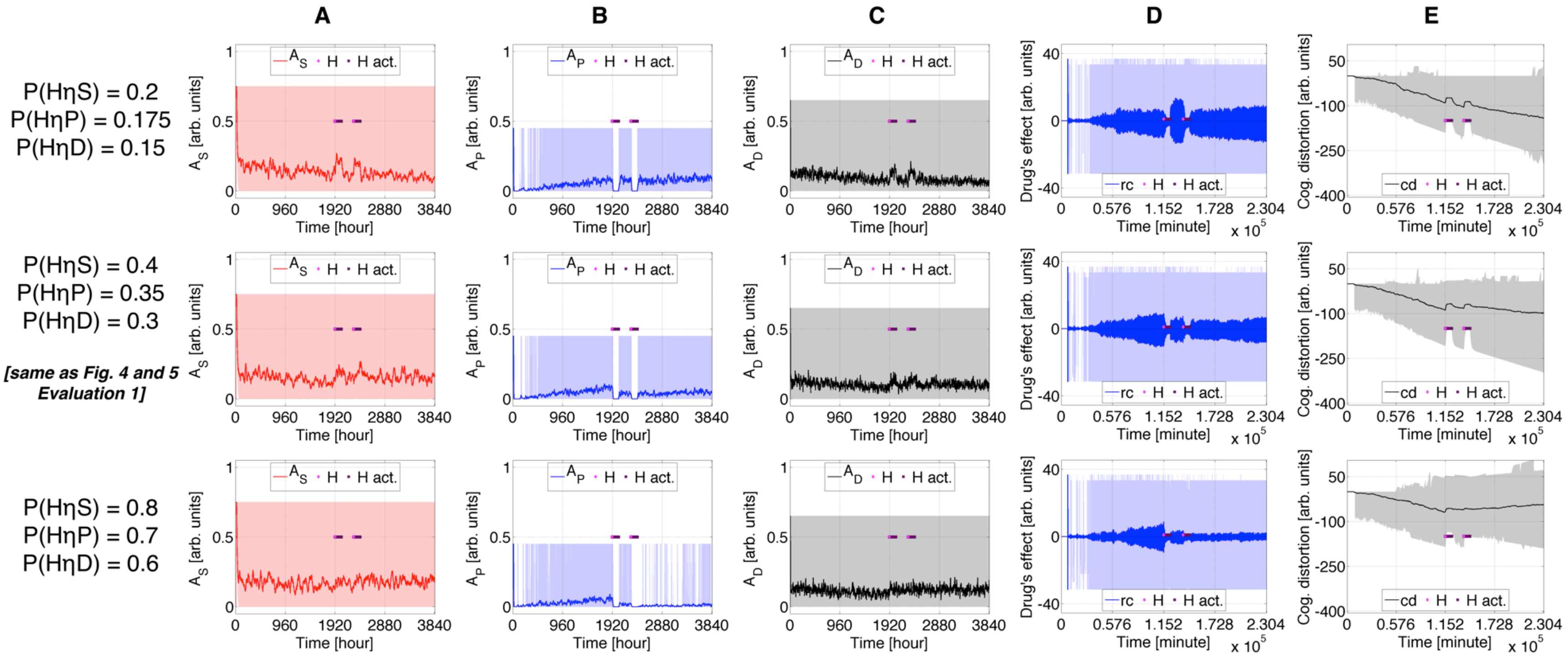

Figure S12: Details of simulations presented in Figures S9 and S10. Simulations of virtual behavior for cigarette consumption over a period of 160 days. Cigarettes are available on the fifth day. Results are the average of 100 runs. The recovery process H is activated at $t = 1920$ and $t = 2280$ [hours] (in light pink) and stays active for 120 hours (dark pink). At the end of each therapeutic session, the positive influence that H exerts on ω_S, ω_P, and ω_D become permanent or disappears, depending on arbitrary probabilities. Three sets of probabilities $P(H\eta i)$, with $i \in \{S, P, D\}$, are tested: [0.2, 0.175, 0.15], [0.4, 0.35, 0.3], and [0.8, 0.7, 0.6]. Columns A-C report the external triggers of acute shock AS (red), acute trauma AP (blue), and acute drug priming AD (black); column D the rush/comedown effect rc of drug intakes; and column E the cognitive distortion cd. The shades correspond to 95% simulation envelopes. The abscissas correspond to the simulation's time-steps: hours in columns A-C, minutes in columns D and E.

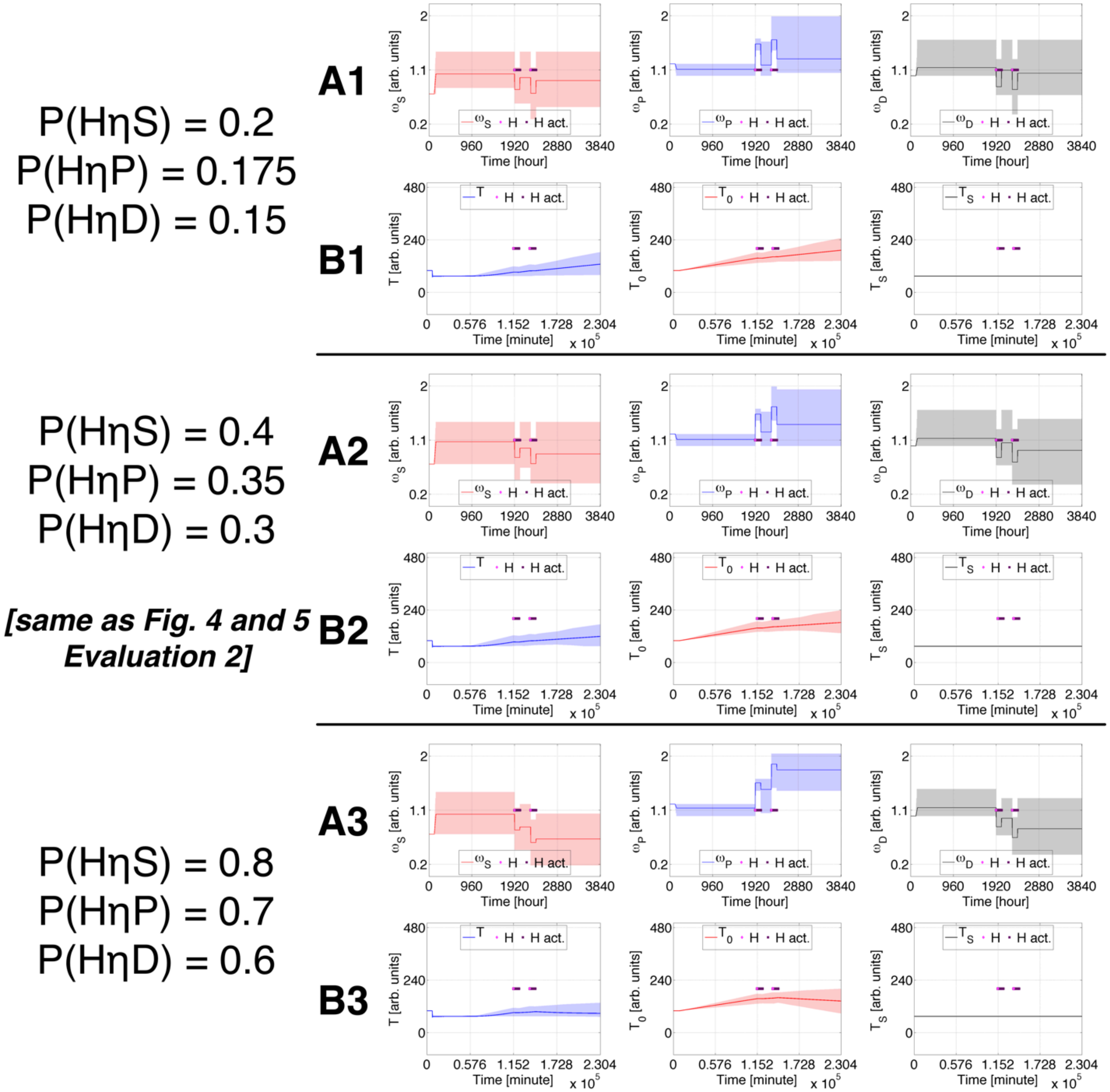

Figure S13: Comparison of different probabilities defining the durability of *H* for conventional therapies, with T_S constant. Simulations of virtual behavior for cigarette consumption over a period of 160 days. Cigarettes are available on the fifth day. Results are the average of 100 runs. The recovery process *H* is activated at $t = 1920$ and $t = 2280$ [hours] (in light pink) and stays active for 120 hours (dark pink). These simulations use the same experimental setup employed in Figures S5 and S6 but have constant T_S. At the end of each therapeutic session, the positive influence that *H* exerts on ω_S, ω_P, and ω_D become permanent or disappears, depending on arbitrary probabilities. Three sets of probabilities $P(H\eta i)$, with $i \in \{S, P, D\}$, are tested: [0.2, 0.175, 0.15], [0.4, 0.35, 0.3], and [0.8, 0.7, 0.6]. Rows A1-A3 report the evolution of cognitive weights ω_S (red), ω_P (blue), and ω_D (black); rows B1-B3 the progression of T (blue), T_0 (red), and T_S (black); column C the virtual subject's mood; and column D the average number of drug intakes (red), and the abstinence index (blue) over time. The shades correspond to 95% simulation envelopes. The simulation time-scales are multiple: hours for rows A1-A3, and minutes for rows B1-B3.

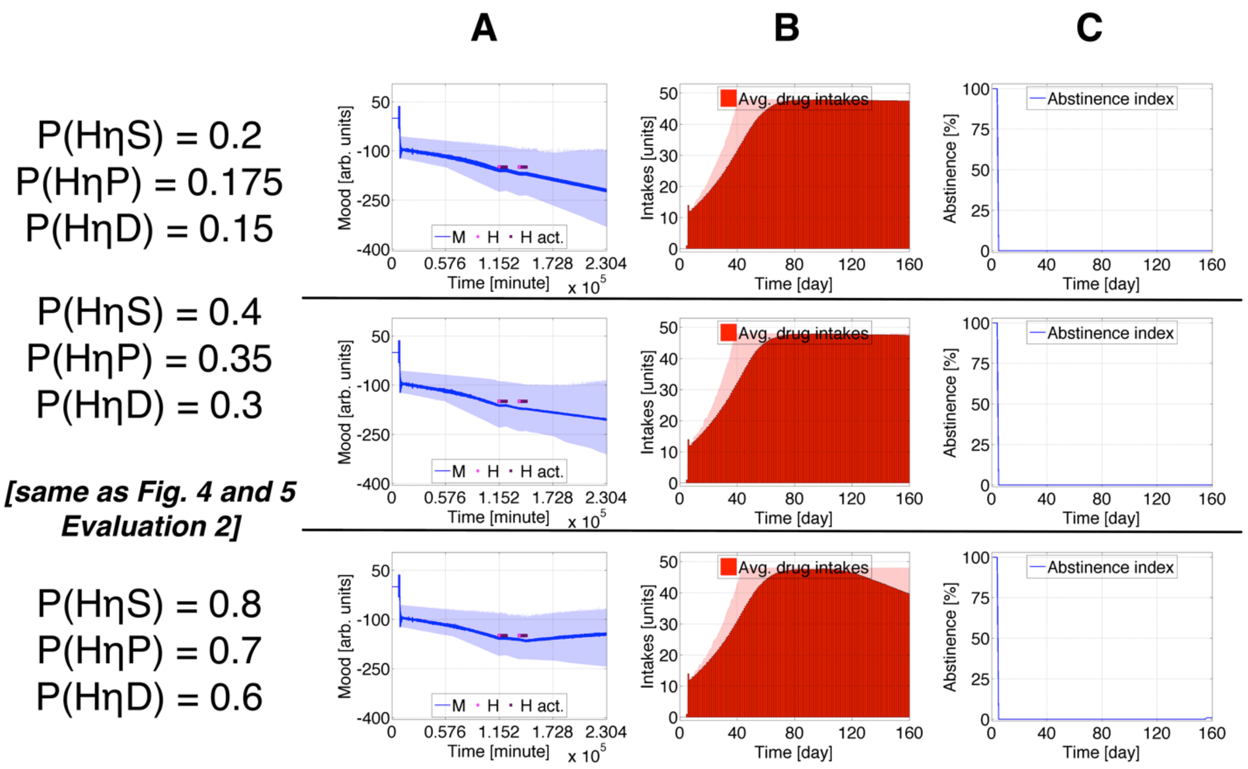

Figure S14: Comparison of different probabilities defining the durability of *H* for conventional therapies, with T_S constant. Simulations of virtual behavior for cigarette consumption over a period of 160 days. Cigarettes are available on the fifth day. Results are the average of 100 runs. The recovery process *H* is activated at $t = 1920$ and $t = 2280$ [hours] (in light pink) and stays active for 120 hours (dark pink). These simulations use the same experimental setup employed in Figures S5 and S6 but have constant T_S. At the end of each therapeutic session, the positive influence that *H* exerts on ω_S, ω_P, and ω_D become permanent or disappears, depending on arbitrary probabilities. Three sets of probabilities $P(H\eta i)$, with $i \in \{S, P, D\}$, are tested: [0.2, 0.175, 0.15], [0.4, 0.35, 0.3], and [0.8, 0.7, 0.6]. Column A reports the virtual subject's mood; column B the average number of drug intakes, and column C the abstinence index over time. The shades correspond to 95% simulation envelopes. The simulation time-scales are multiple: minutes for column A, days columns B and C.

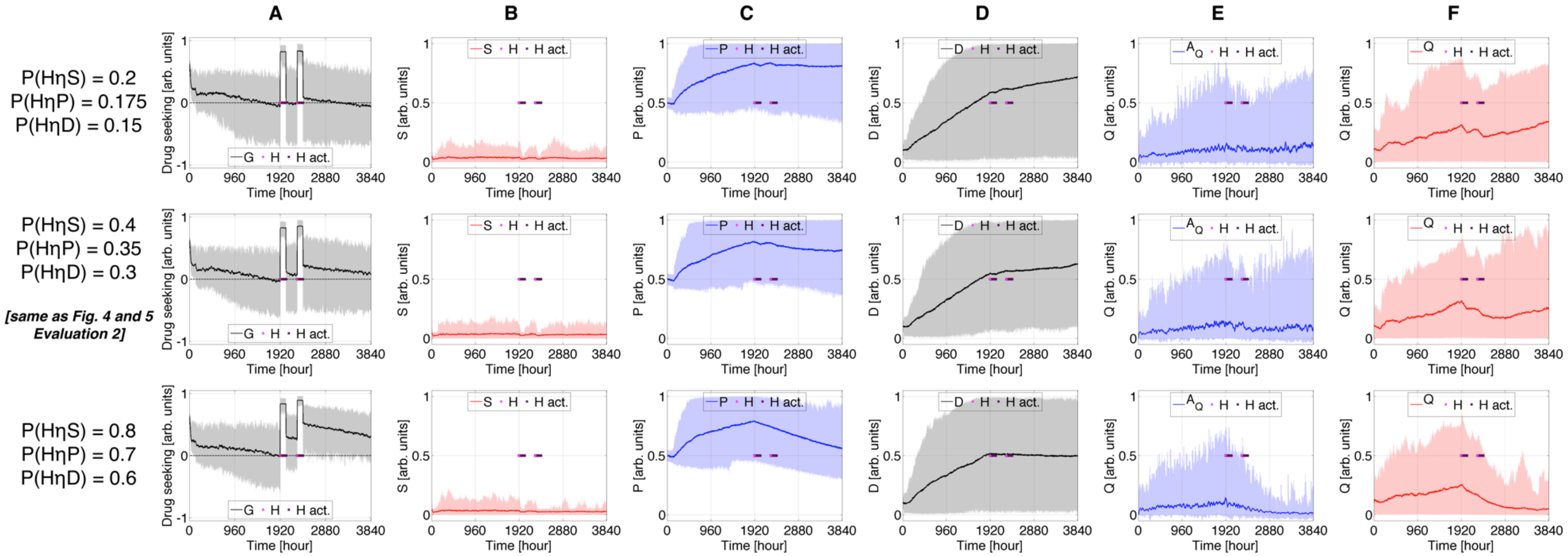

Figure S15: Details of simulations presented in Figures S13 and S14. Simulations of virtual behavior for cigarette consumption over a period of 160 days. Cigarettes are available on the fifth day. Results are the average of 100 runs. The recovery process H is activated at $t = 1920$ and $t = 2280$ [hours] (in light pink) and stays active for 120 hours (dark pink). These simulations use the same experimental setup employed in Figures S5 and S6 but have constant T_S. At the end of each therapeutic session, the positive influence that H exerts on ω_S, ω_P, and ω_D become permanent or disappears, depending on arbitrary probabilities. Three sets of probabilities $P(H\eta i)$, with $i \in$ {S, P, D}, are tested: [0.2, 0.175, 0.15], [0.4, 0.35, 0.3], and [0.8, 0.7, 0.6]. Column A reports the evolution of drug-seeking behavior G; columns B-D the progression of internal processes stress S (red), pain P (blue), and drug craving D (black); columns E-F the external trigger acute drug cue AQ (blue) and the internal process of saliency to drug cues Q (red). The shades correspond to 95% simulation envelopes.

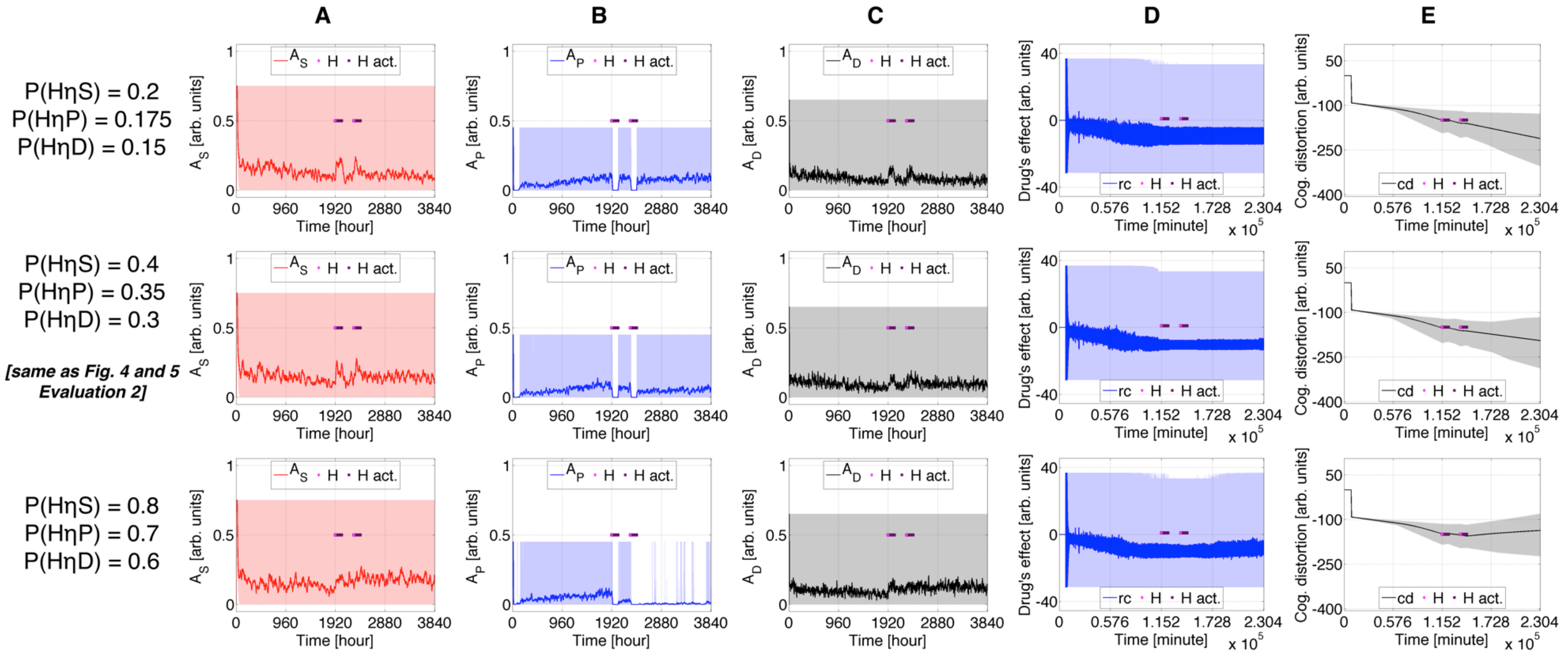

Figure S16: Details of simulations presented in Figures S13 and S14. Simulations of virtual behavior for cigarette consumption over a period of 160 days. Cigarettes are available on the fifth day. Results are the average of 100 runs. The recovery process H is activated at $t = 1920$ and $t = 2280$ [hours] (in light pink) and stays active for 120 hours (dark pink). These simulations use the same experimental setup employed in Figures S5 and S6 but have constant T_S. At the end of each therapeutic session, the positive influence that H exerts on ω_S, ω_P, and ω_D become permanent or disappears, depending on arbitrary probabilities. Three sets of probabilities $P(H\eta i)$, with $i \in \{S, P, D\}$, are tested: [0.2, 0.175, 0.15], [0.4, 0.35, 0.3], and [0.8, 0.7, 0.6]. Columns A-C report the external triggers of acute shock AS (red), acute trauma AP (blue), and acute drug priming AD (black); column D the rush/comedown effect rc of drug intakes; and column E the cognitive distortion cd. The shades correspond to 95% simulation envelopes. The abscissas correspond to the simulation's time-steps: hours in columns A-C, minutes in columns D and E.

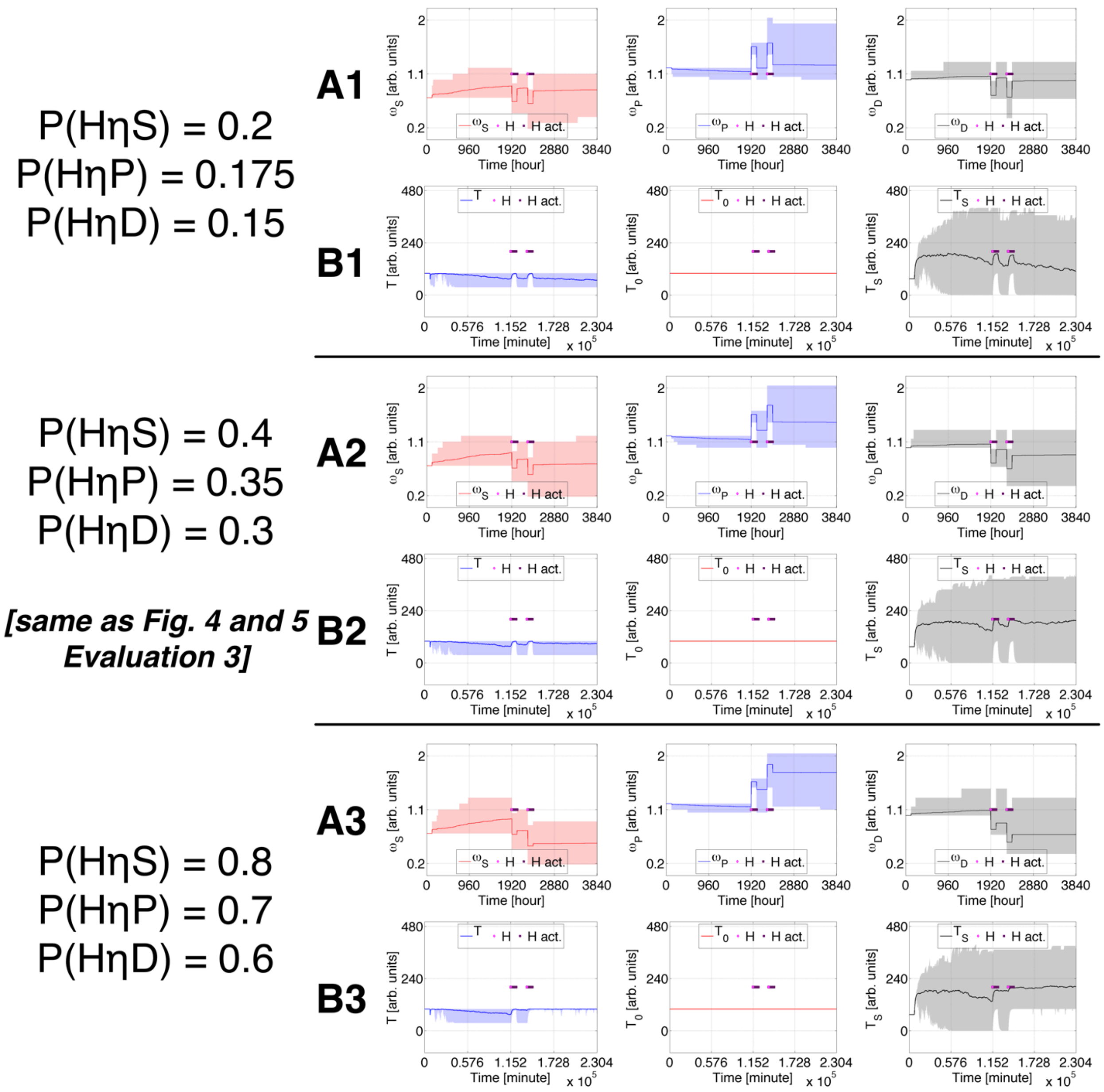

Figure S17: Comparison of different probabilities defining the durability of *H* for conventional therapies, with T_0 constant. Simulations of virtual behavior for cigarette consumption over a period of 160 days. Cigarettes are available on the fifth day. Results are the average of 100 runs. The recovery process *H* is activated at $t = 1920$ and $t = 2280$ [hours] (in light pink) and stays active for 120 hours (dark pink). These simulations use the same experimental setup employed in Figures S5 and S6 but have constant T_S. At the end of each therapeutic session, the positive influence that *H* exerts on ω_S, ω_P, and ω_D become permanent or disappears, depending on arbitrary probabilities. Three sets of probabilities $P(H\eta i)$, with $i \in \{S, P, D\}$, are tested: [0.2, 0.175, 0.15], [0.4, 0.35, 0.3], and [0.8, 0.7, 0.6]. Rows A1-A3 report the evolution of cognitive weights ω_S (red), ω_P (blue), and ω_D (black); rows B1-B3 the progression of T (blue), T_0 (red), and T_S (black); column C the virtual subject's mood; and column D the average number of drug intakes (red), and the abstinence index (blue) over time. The shades correspond to 95% simulation envelopes. The simulation time-scales are multiple: hours for rows A1-A3, and minutes for rows B1-B3.

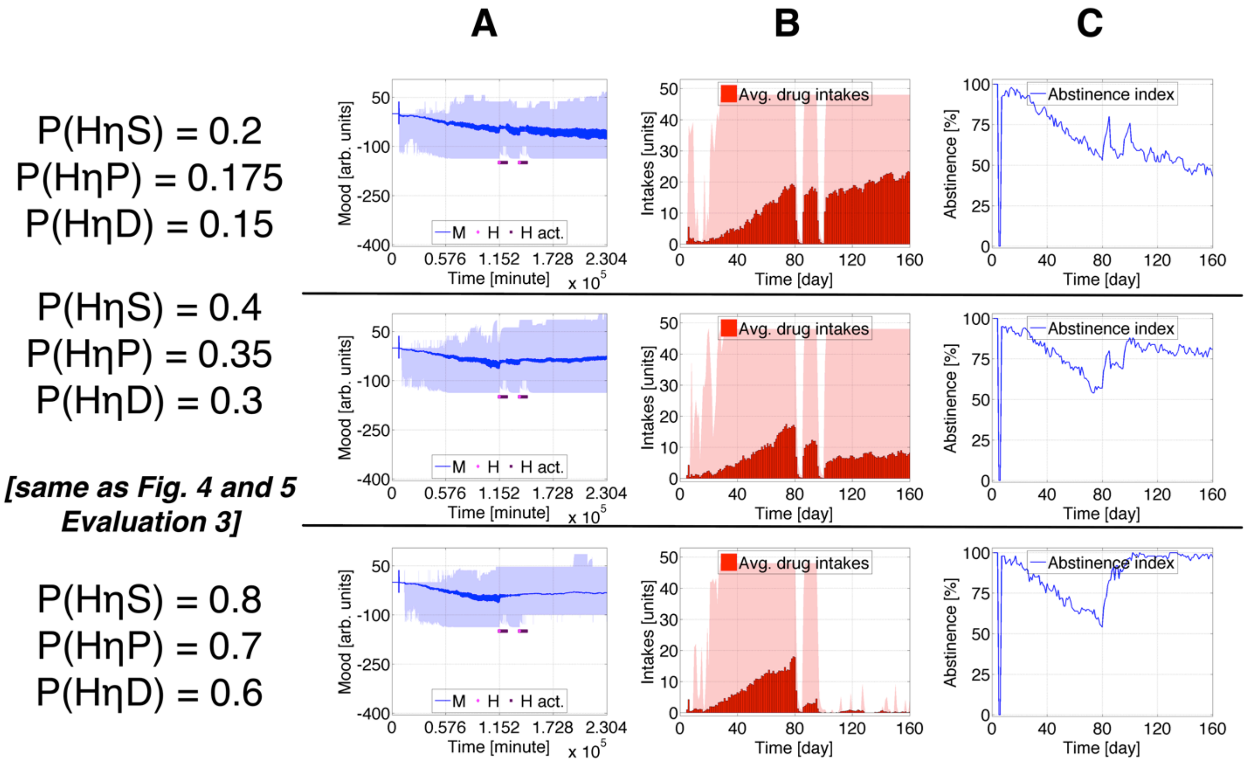

Figure S18: Comparison of different probabilities defining the durability of *H* for conventional therapies, with T_0 constant. Simulations of virtual behavior for cigarette consumption over a period of 160 days. Cigarettes are available on the fifth day. Results are the average of 100 runs. The recovery process *H* is activated at $t = 1920$ and $t = 2280$ [hours] (in light pink) and stays active for 120 hours (dark pink). These simulations use the same experimental setup employed in Figures S5 and S6 but have constant T_S. At the end of each therapeutic session, the positive influence that *H* exerts on ω_S, ω_P, and ω_D become permanent or disappears, depending on arbitrary probabilities. Three sets of probabilities $P(H\eta i)$, with $i \in \{S, P, D\}$, are tested: [0.2, 0.175, 0.15], [0.4, 0.35, 0.3], and [0.8, 0.7, 0.6]. Column A reports the virtual subject's mood; column B the average number of drug intakes, and column C the abstinence index over time. The shades correspond to 95% simulation envelopes. The simulation time-scales are multiple: minutes for column A, days columns B and C.

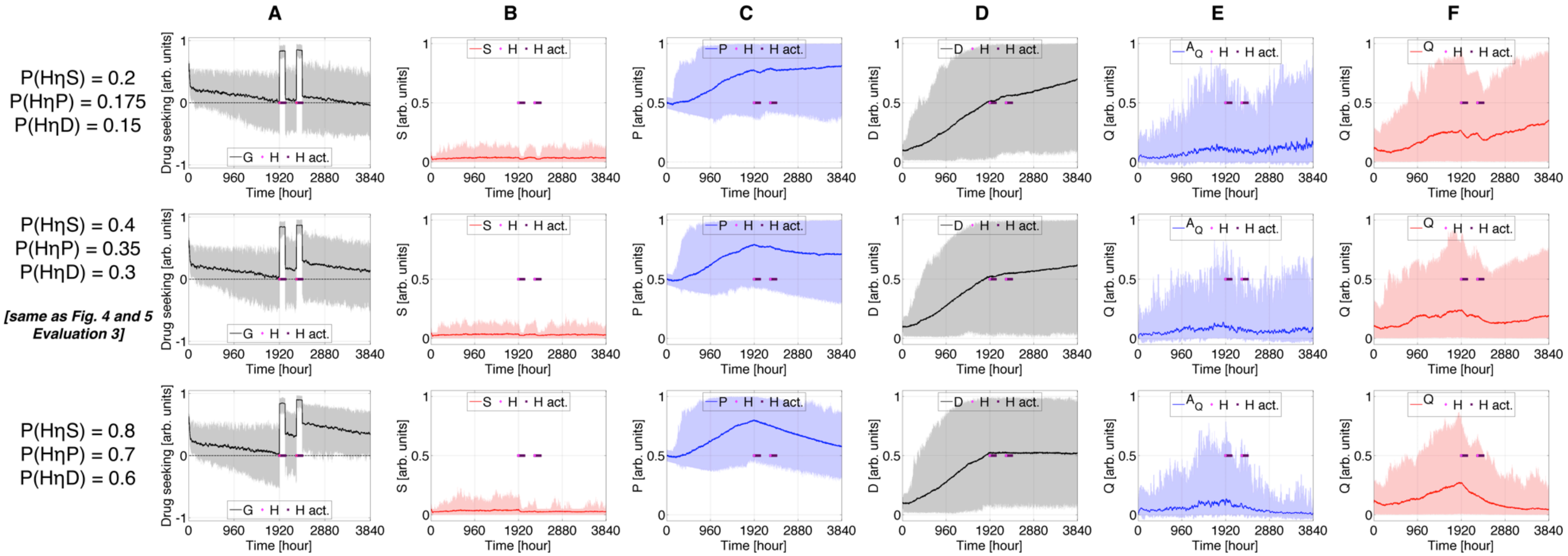

Figure S19: Details of simulations presented in Figures S17 and S18. Simulations of virtual behavior for cigarette consumption over a period of 160 days. Cigarettes are available on the fifth day. Results are the average of 100 runs. The recovery process H is activated at $t = 1920$ and $t = 2280$ [hours] (in light pink) and stays active for 120 hours (dark pink). These simulations use the same experimental setup employed in Figures S5 and S6 but have constant T_S. At the end of each therapeutic session, the positive influence that H exerts on ω_S, ω_P, and ω_D become permanent or disappears, depending on arbitrary probabilities. Three sets of probabilities $P(H\eta i)$, with $i \in \{S, P, D\}$, are tested: [0.2, 0.175, 0.15], [0.4, 0.35, 0.3], and [0.8, 0.7, 0.6]. Column A reports the evolution of drug-seeking behavior G; columns B-D the progression of internal processes stress S (red), pain P (blue), and drug craving D (black); columns E-F the external trigger acute drug cue AQ (blue) and the internal process of saliency to drug cues Q (red). The shades correspond to 95% simulation envelopes.

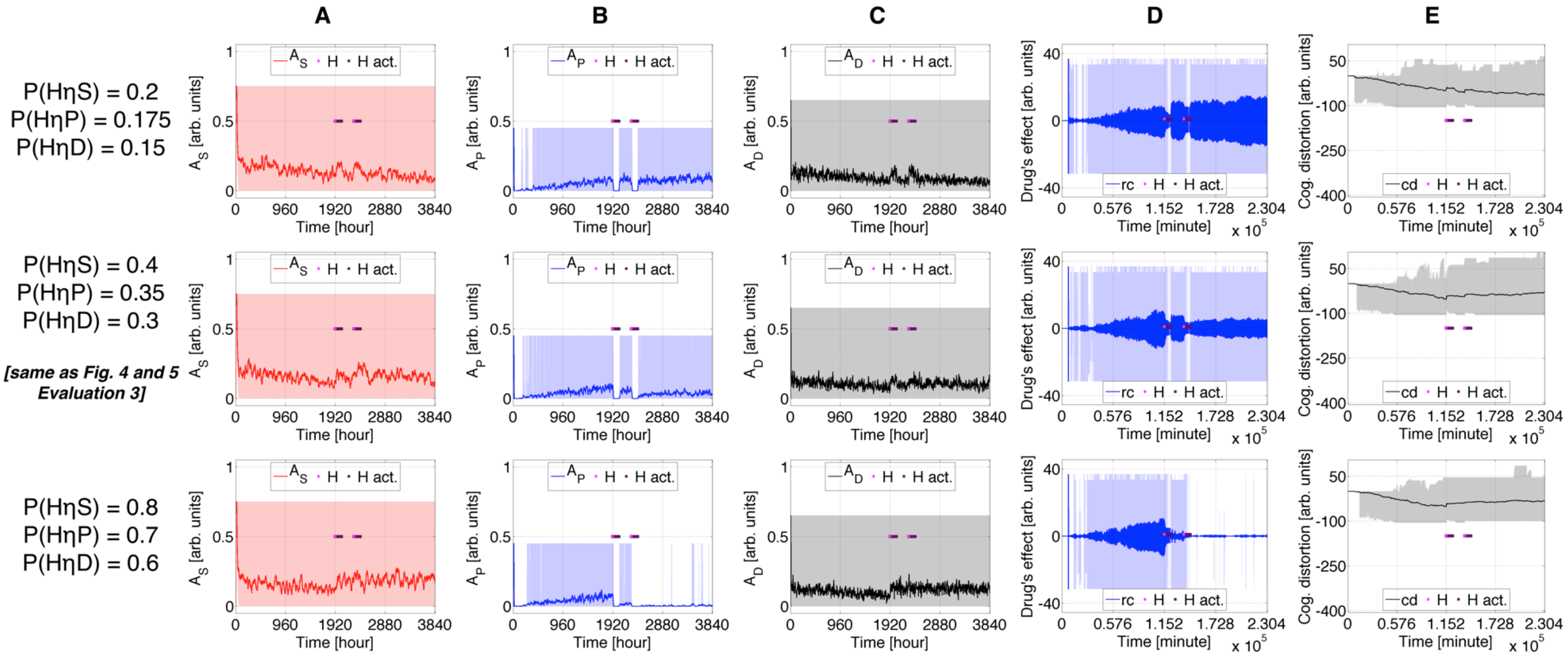

Figure S20: Details of simulations presented in Figures S17 and S18. Simulations of virtual behavior for cigarette consumption over a period of 160 days. Cigarettes are available on the fifth day. Results are the average of 100 runs. The recovery process H is activated at $t = 1920$ and $t = 2280$ [hours] (in light pink) and stays active for 120 hours (dark pink). These simulations use the same experimental setup employed in Figures S5 and S6 but have constant T_S. At the end of each therapeutic session, the positive influence that H exerts on ω_S, ω_P, and ω_D become permanent or disappears, depending on arbitrary probabilities. Three sets of probabilities $P(H\eta i)$, with $i \in \{S, P, D\}$, are tested: [0.2, 0.175, 0.15], [0.4, 0.35, 0.3], and [0.8, 0.7, 0.6]. Columns A-C report the external triggers of acute shock AS (red), acute trauma AP (blue), and acute drug priming AD (black); column D the rush/comedown effect rc of drug intakes; and column E the cognitive distortion cd. The shades correspond to 95% simulation envelopes. The abscissas correspond to the simulation's time-steps: hours in columns A-C, minutes in columns D and E.

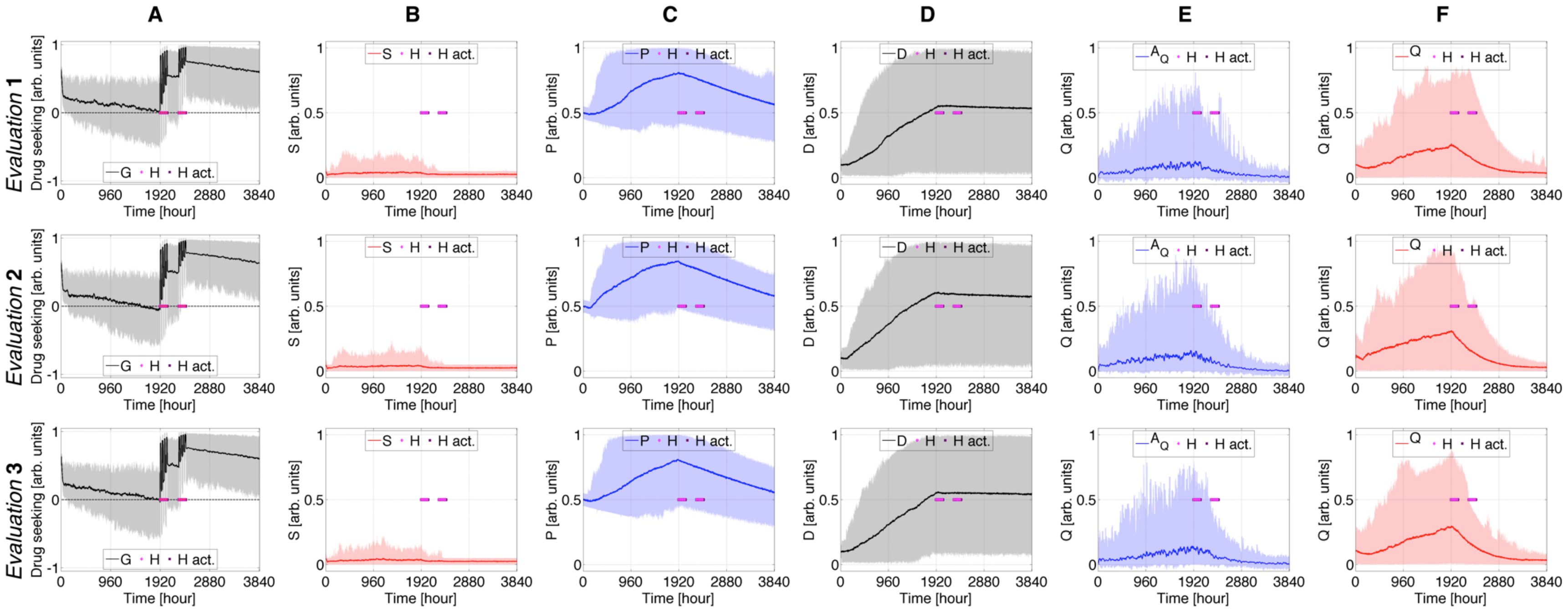

Figure S21: Details of Case Study 3 presented in Figures 6 and 7. Simulations of virtual behavior for cigarette consumption over a period of 160 days. Cigarettes are available on the fifth day. Results are the average of 100 runs. Evaluation 1 simulates both T_S and T_0 as time-dependent processes. T_S is constant and T_0 time-dependent in Evaluation 2, T_S is time-dependent and T_0 constant in Evaluation 3. In all Evaluations, the recovery process H is activated at $t \in \{1920, 1960, 2000, 2040, 2280, 2320, 2360, 2400\}$ [hours] (in light pink) and stays active for 15 hours (dark pink). Column A reports the evolution of drug-seeking behavior G; columns B-D the progression of internal processes stress S (red), pain P (blue), and drug craving D (black); columns E-F the external trigger acute drug cue AQ (blue) and the internal process of saliency to drug cues Q (red). The shades correspond to 95% simulation envelopes.

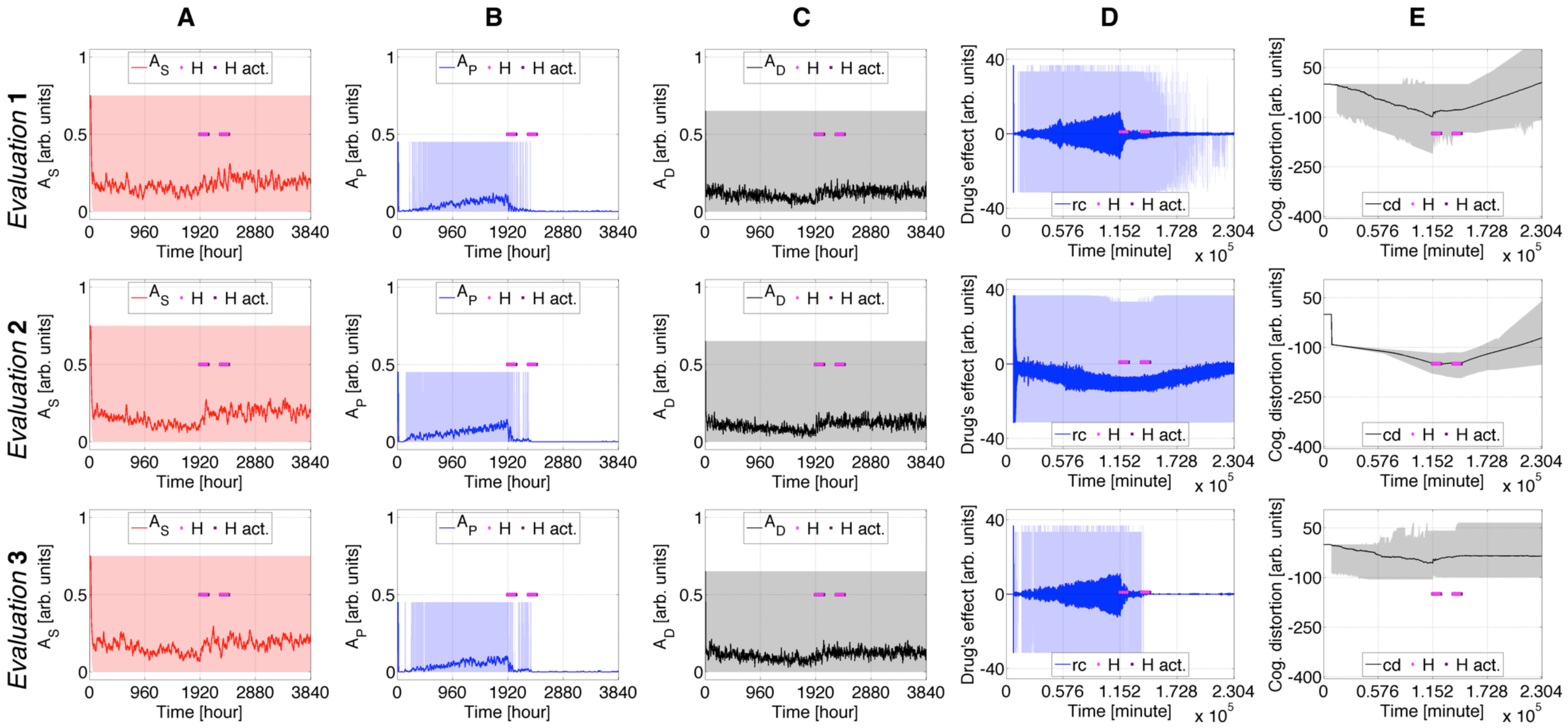

Figure S22: Details of Case Study 3 presented in Figures 6 and 7. Simulations of virtual behavior for cigarette consumption over a period of 160 days. Cigarettes are available on the fifth day. Results are the average of 100 runs. Evaluation 1 simulates both T_S and T_0 as time-dependent processes. T_S is constant and T_0 time-dependent in Evaluation 2, T_S is time-dependent and T_0 constant in Evaluation 3. In all Evaluations, the recovery process H is activated at $t \in \{1920, 1960, 2000, 2040, 2280, 2320, 2360, 2400\}$ [hours] (in light pink) and stays active for 15 hours (dark pink). Columns A-C report the external triggers of acute shock AS (red), acute trauma AP (blue), and acute drug priming AD (black); column D the rush/comedown effect rc of drug intakes; and column E the cognitive distortion cd. The shades correspond to 95% simulation envelopes. The abscissas correspond to the simulation's time-steps: hours in columns A-C, minutes in columns D and E.

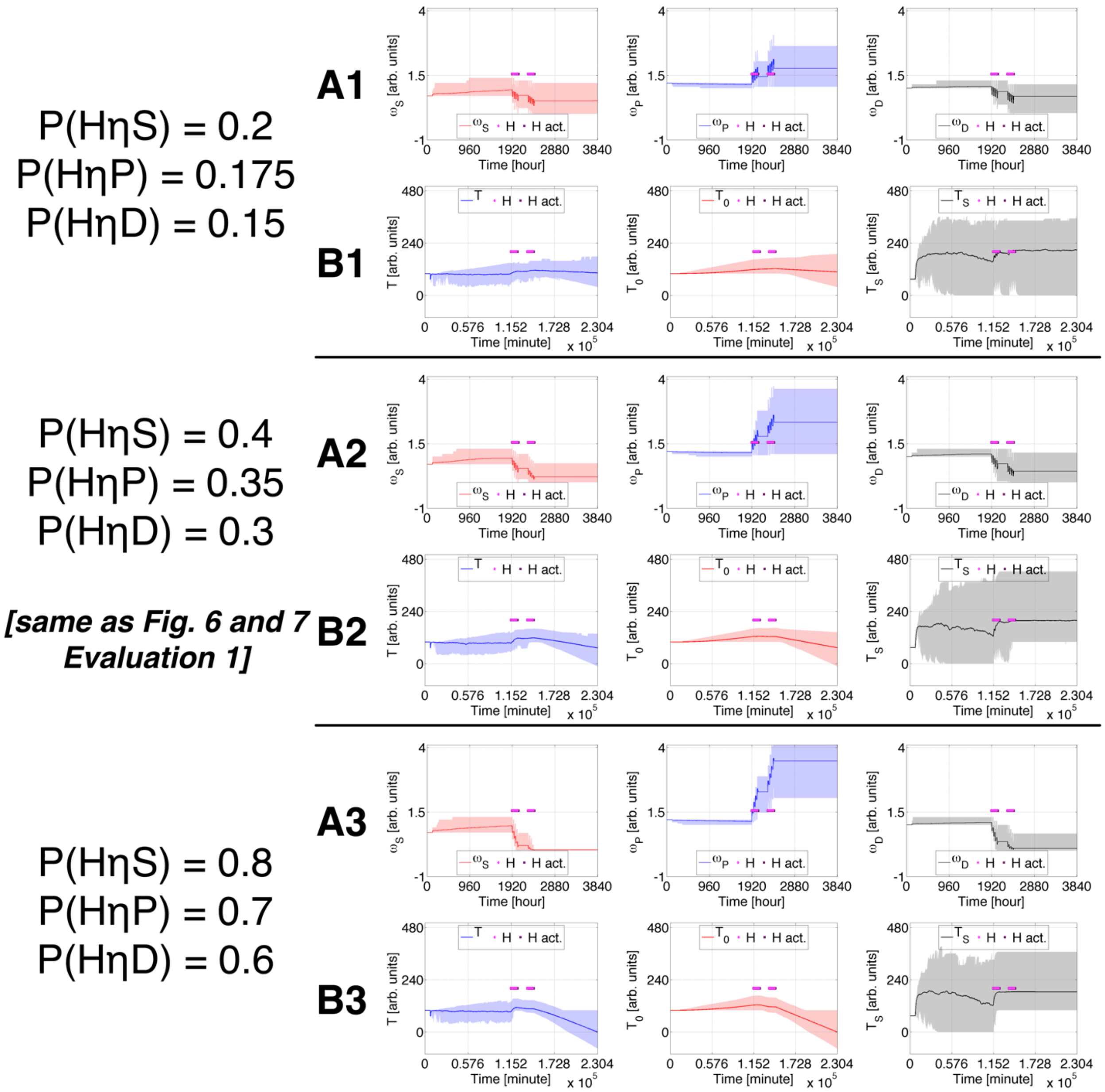

Figure S23: Comparison of different probabilities defining the durability of H for alternative treatments, with both T_S and T_0 time-dependent. Simulations of virtual behavior for cigarette consumption over a period of 160 days. Cigarettes are available on the fifth day. Results are the average of 100 runs. The recovery process H is activated at $t \in \{1920, 1960, 2000, 2040, 2280, 2320, 2360, 2400\}$ [hours] (in light pink) and stays active for 15 hours (dark pink). At the end of each therapeutic session, the positive influence that H exerts on ω_S, ω_P, and ω_D become permanent or disappears, depending on arbitrary probabilities. Three sets of probabilities $P(H\eta i)$, with $i \in \{S, P, D\}$, are tested: [0.2, 0.175, 0.15], [0.4, 0.35, 0.3], and [0.8, 0.7, 0.6]. Rows A1-A3 report the evolution of cognitive weights ω_S (red), ω_P (blue), and ω_D (black); rows B1-B3 the progression of T (blue), T_0 (red), and T_S (black); column C the virtual subject's mood; and column D the average number of drug intakes (red), and the abstinence index (blue) over time. The shades correspond to 95% simulation envelopes. The simulation time-scales are multiple: hours for rows A1-A3, and minutes for rows B1-B3.

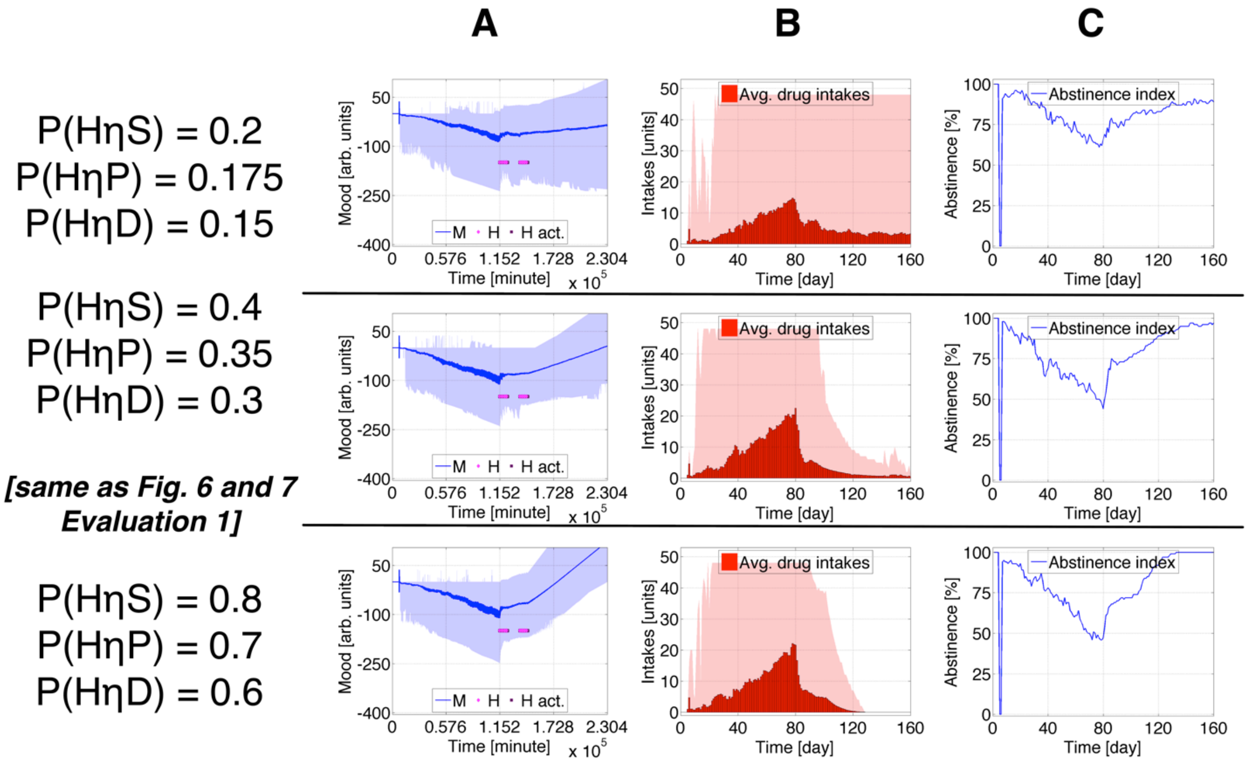

Figure S24: Comparison of different probabilities defining the durability of *H* for alternative treatments, with both T_S and T_0 time-dependent. Simulations of virtual behavior for cigarette consumption over a period of 160 days. Cigarettes are available on the fifth day. Results are the average of 100 runs. The recovery process *H* is activated at $t \in \{1920, 1960, 2000, 2040, 2280, 2320, 2360, 2400\}$ [hours] (in light pink) and stays active for 15 hours (dark pink). At the end of each therapeutic session, the positive influence that *H* exerts on ω_S, ω_P, and ω_D become permanent or disappears, depending on arbitrary probabilities. Three sets of probabilities $P(H\eta i)$, with $i \in \{S, P, D\}$, are tested: [0.2, 0.175, 0.15], [0.4, 0.35, 0.3], and [0.8, 0.7, 0.6]. Column A reports the virtual subject's mood; column B the average number of drug intakes, and column C the abstinence index over time. The shades correspond to 95% simulation envelopes. The simulation time-scales are multiple: minutes for column A, days columns B and C.

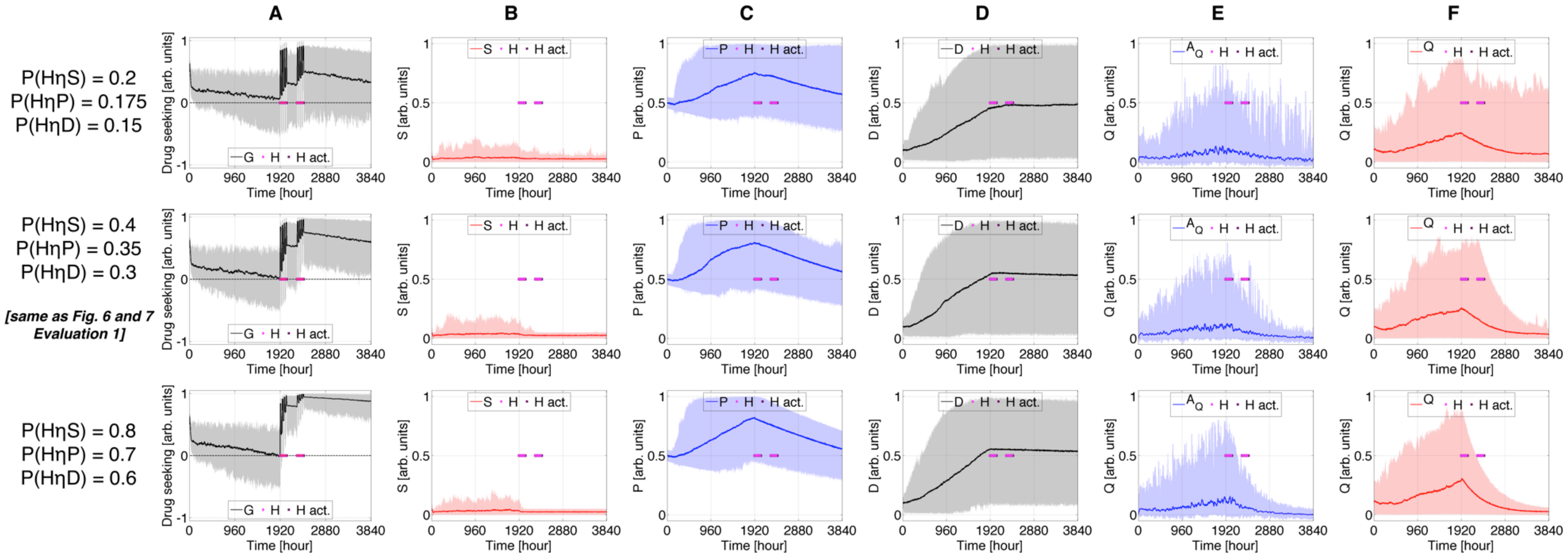

Figure S25: Details of simulations presented in Figures S23 and S24. Simulations of virtual behavior for cigarette consumption over a period of 160 days. Cigarettes are available on the fifth day. Results are the average of 100 runs. The recovery process H is activated at $t \in \{1920, 1960, 2000, 2040, 2280, 2320, 2360, 2400\}$ [hours] (in light pink) and stays active for 15 hours (dark pink). At the end of each therapeutic session, the positive influence that H exerts on ω_S, ω_P, and ω_D become permanent or disappears, depending on arbitrary probabilities. Three sets of probabilities $P(H\eta i)$, with $i \in \{S, P, D\}$, are tested: [0.2, 0.175, 0.15], [0.4, 0.35, 0.3], and [0.8, 0.7, 0.6]. Column A reports the evolution of drug-seeking behavior G; columns B-D the progression of internal processes stress S (red), pain P (blue), and drug craving D (black); columns E-F the external trigger acute drug cue AQ (blue) and the internal process of saliency to drug cues Q (red). The shades correspond to 95% simulation envelopes.

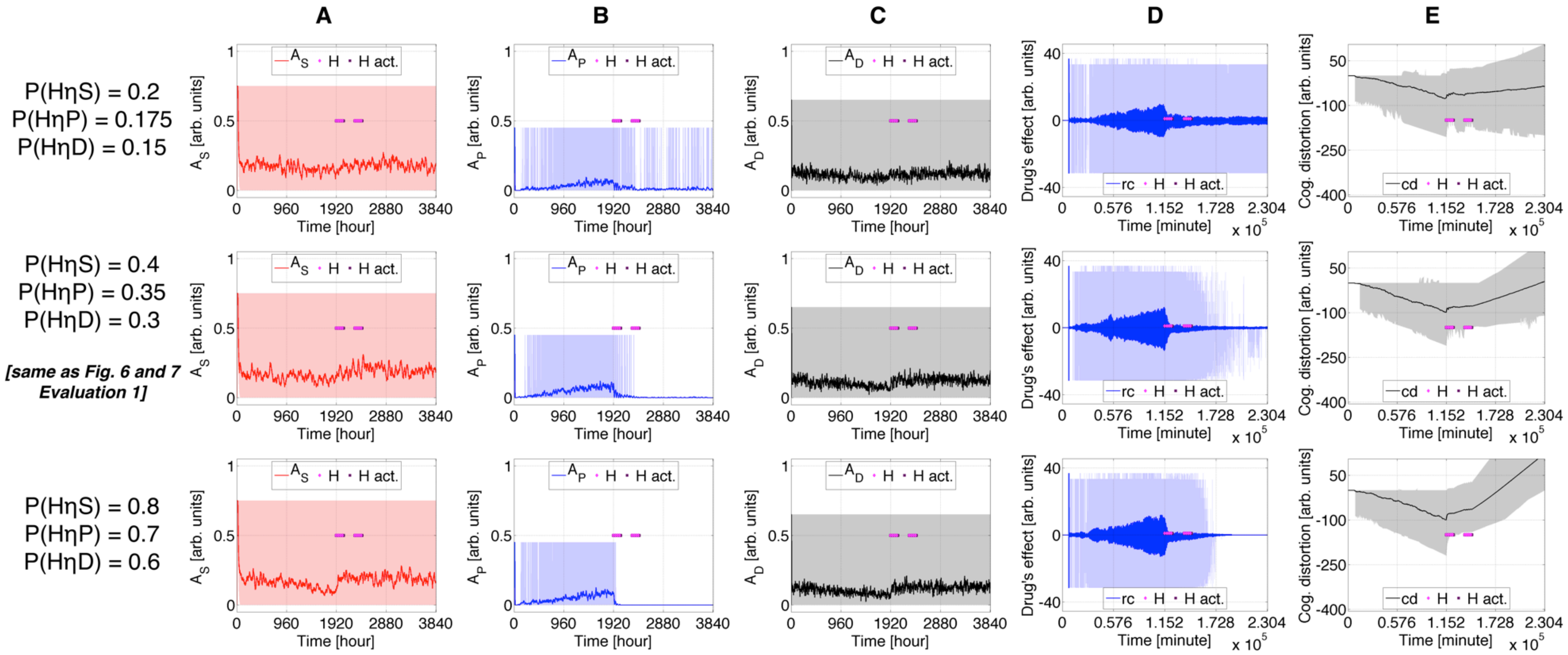

Figure S26: Details of simulations presented in Figures S23 and S24. Simulations of virtual behavior for cigarette consumption over a period of 160 days. Cigarettes are available on the fifth day. Results are the average of 100 runs. The recovery process H is activated at $t \in \{1920, 1960, 2000, 2040, 2280, 2320, 2360, 2400\}$ [hours] (in light pink) and stays active for 15 hours (dark pink). At the end of each therapeutic session, the positive influence that H exerts on ω_S, ω_P, and ω_D become permanent or disappears, depending on arbitrary probabilities. Three sets of probabilities $P(H\eta i)$, with $i \in \{S, P, D\}$, are tested: [0.2, 0.175, 0.15], [0.4, 0.35, 0.3], and [0.8, 0.7, 0.6]. Columns A-C report the external triggers of acute shock AS (red), acute trauma AP (blue), and acute drug priming AD (black); column D the rush/comedown effect rc of drug intakes; and column E the cognitive distortion cd. The shades correspond to 95% simulation envelopes. The abscissas correspond to the simulation's time-steps: hours in columns A-C, minutes in columns D and E.

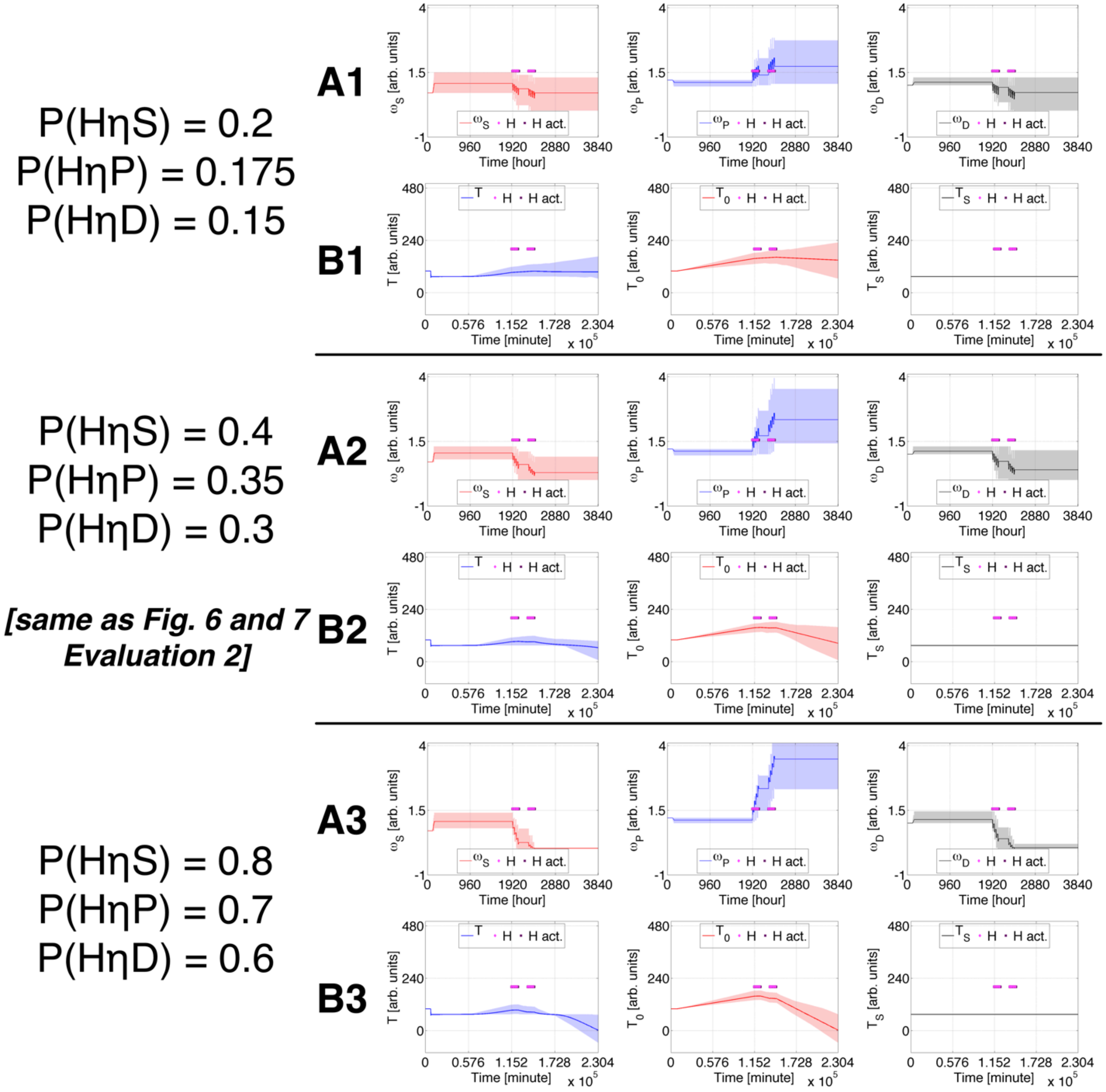

Figure S27: Comparison of different probabilities defining the durability of *H* for alternative treatments, with T_S constant. Simulations of virtual behavior for cigarette consumption over a period of 160 days. Cigarettes are available on the fifth day. Results are the average of 100 runs. The recovery process *H* is activated at $t \in \{1920, 1960, 2000, 2040, 2280, 2320, 2360, 2400\}$ [hours] (in light pink) and stays active for 15 hours (dark pink). These simulations use the same experimental setup employed in Figures S12 and S13 but have constant T_S. At the end of each therapeutic session, the positive influence that *H* exerts on ω_S, ω_P, and ω_D become permanent or disappears, depending on arbitrary probabilities. Three sets of probabilities $P(H\eta i)$, with $i \in \{S, P, D\}$, are tested: [0.2, 0.175, 0.15], [0.4, 0.35, 0.3], and [0.8, 0.7, 0.6]. Rows A1-A3 report the evolution of cognitive weights ω_S (red), ω_P (blue), and ω_D (black); rows B1-B3 the progression of T (blue), T_0 (red), and T_S (black); column C the virtual subject's mood; and column D the average number of drug intakes (red), and the abstinence index (blue) over time. The shades correspond to 95% simulation envelopes. The simulation time-scales are multiple: hours for rows A1-A3, and minutes for rows B1-B3.

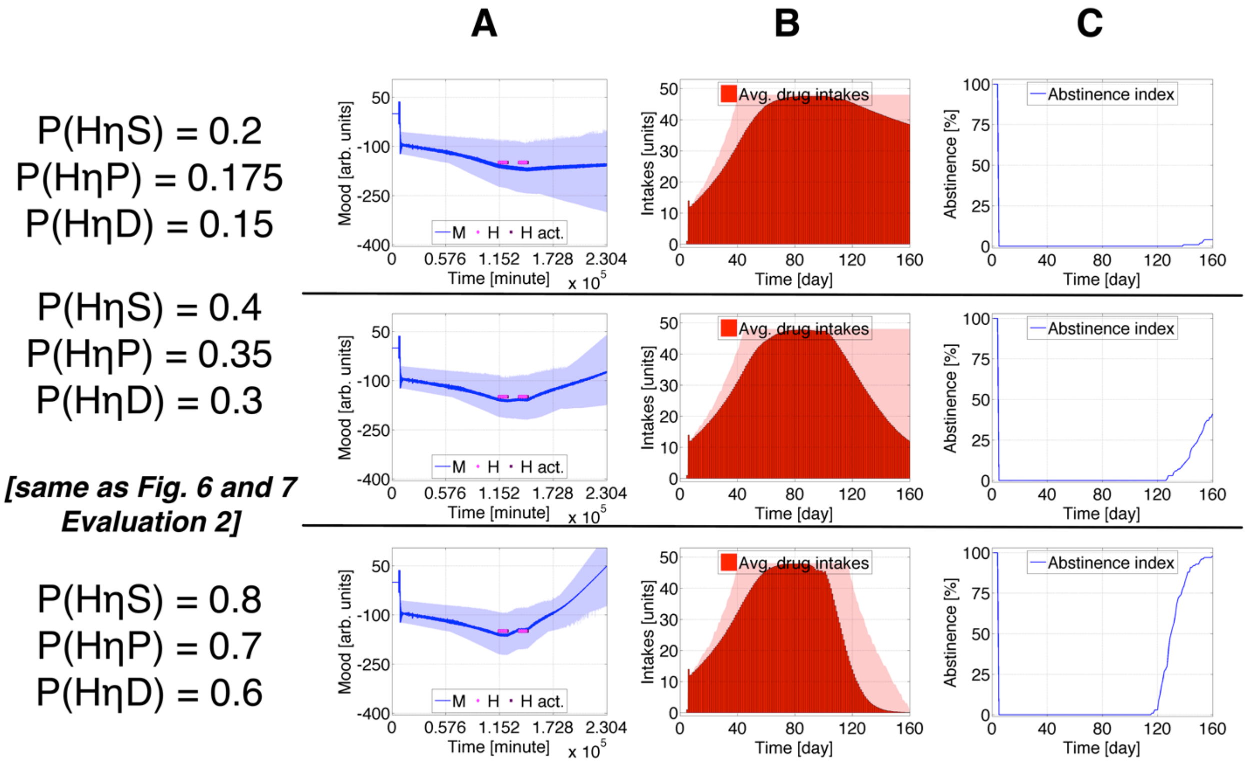

Figure S28: Comparison of different probabilities defining the durability of *H* for alternative treatments, with T_S constant. Simulations of virtual behavior for cigarette consumption over a period of 160 days. Cigarettes are available on the fifth day. Results are the average of 100 runs. The recovery process *H* is activated at $t \in \{1920, 1960, 2000, 2040, 2280, 2320, 2360, 2400\}$ [hours] (in light pink) and stays active for 15 hours (dark pink). These simulations use the same experimental setup employed in Figures S12 and S13 but have constant T_S. At the end of each therapeutic session, the positive influence that *H* exerts on ω_S, ω_P, and ω_D become permanent or disappears, depending on arbitrary probabilities. Three sets of probabilities $P(H\eta i)$, with $i \in \{S, P, D\}$, are tested: [0.2, 0.175, 0.15], [0.4, 0.35, 0.3], and [0.8, 0.7, 0.6]. Column A reports the virtual subject's mood; column B the average number of drug intakes, and column C the abstinence index over time. The shades correspond to 95% simulation envelopes. The simulation time-scales are multiple: minutes for column A, days columns B and C.

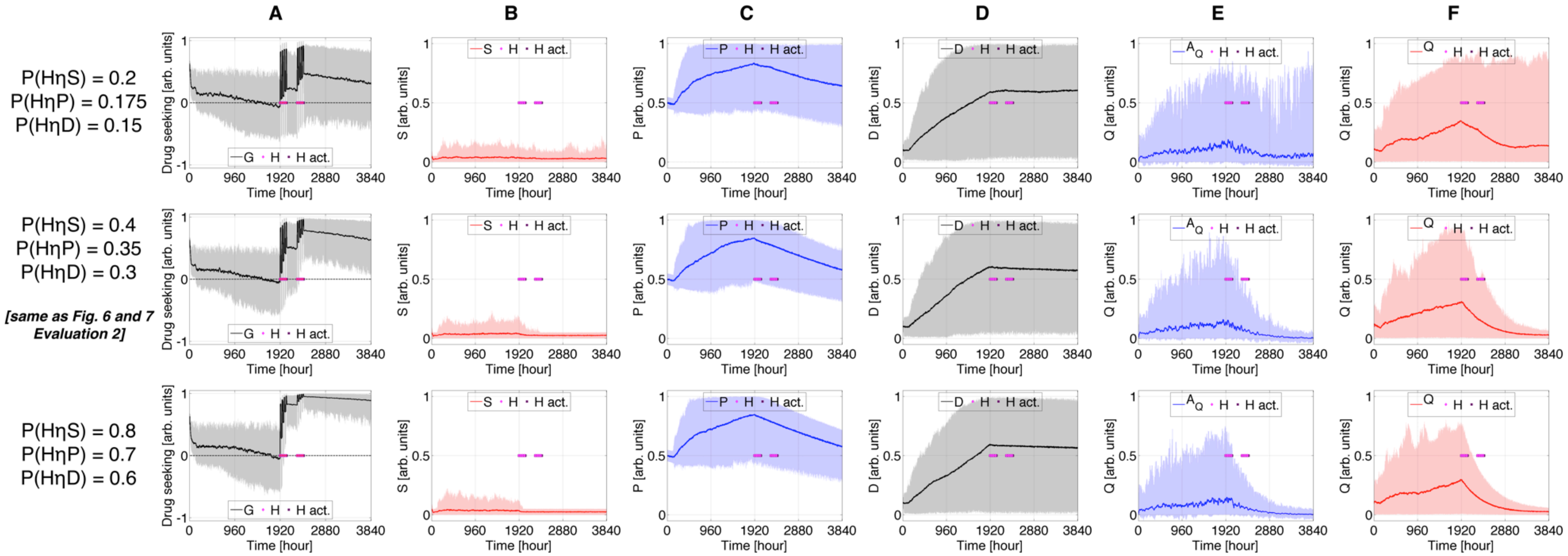

Figure S29: Details of simulations presented in Figures S27 and S28. Simulations of virtual behavior for cigarette consumption over a period of 160 days. Cigarettes are available on the fifth day. Results are the average of 100 runs. The recovery process H is activated at $t \in \{1920, 1960, 2000, 2040, 2280, 2320, 2360, 2400\}$ [hours] (in light pink) and stays active for 15 hours (dark pink). These simulations use the same experimental setup employed in Figures S12 and S13 but have constant T_S. At the end of each therapeutic session, the positive influence that H exerts on ω_S, ω_P, and ω_D become permanent or disappears, depending on arbitrary probabilities. Three sets of probabilities $P(H\eta i)$, with $i \in \{S, P, D\}$, are tested: [0.2, 0.175, 0.15], [0.4, 0.35, 0.3], and [0.8, 0.7, 0.6]. Column A reports the evolution of drug-seeking behavior G; columns B-D the progression of internal processes stress S (red), pain P (blue), and drug craving D (black); columns E-F the external trigger acute drug cue AQ (blue) and the internal process of saliency to drug cues Q (red). The shades correspond to 95% simulation envelopes.

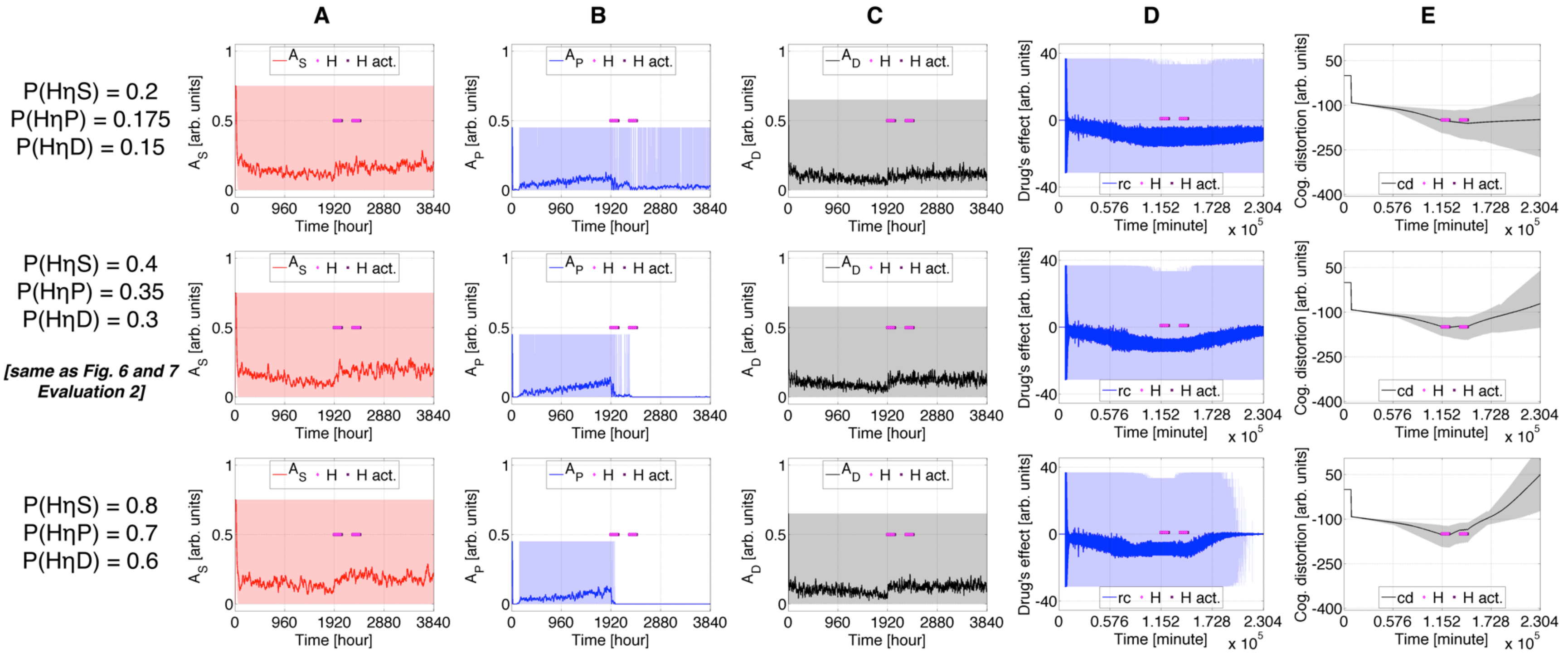

Figure S30: Details of simulations presented in Figures S27 and S28. Simulations of virtual behavior for cigarette consumption over a period of 160 days. Cigarettes are available on the fifth day. Results are the average of 100 runs. The recovery process H is activated at $t \in \{1920, 1960, 2000, 2040, 2280, 2320, 2360, 2400\}$ [hours] (in light pink) and stays active for 15 hours (dark pink). These simulations use the same experimental setup employed in Figures S12 and S13 but have constant T_S. At the end of each therapeutic session, the positive influence that H exerts on ω_S, ω_P, and ω_D become permanent or disappears, depending on arbitrary probabilities. Three sets of probabilities $P(H\eta i)$, with $i \in \{S, P, D\}$, are tested: [0.2, 0.175, 0.15], [0.4, 0.35, 0.3], and [0.8, 0.7, 0.6]. Columns A-C report the external triggers of acute shock AS (red), acute trauma AP (blue), and acute drug priming AD (black); column D the rush/comedown effect rc of drug intakes; and column E the cognitive distortion cd. The shades correspond to 95% simulation envelopes. The abscissas correspond to the simulation's time-steps: hours in columns A-C, minutes in columns D and E.

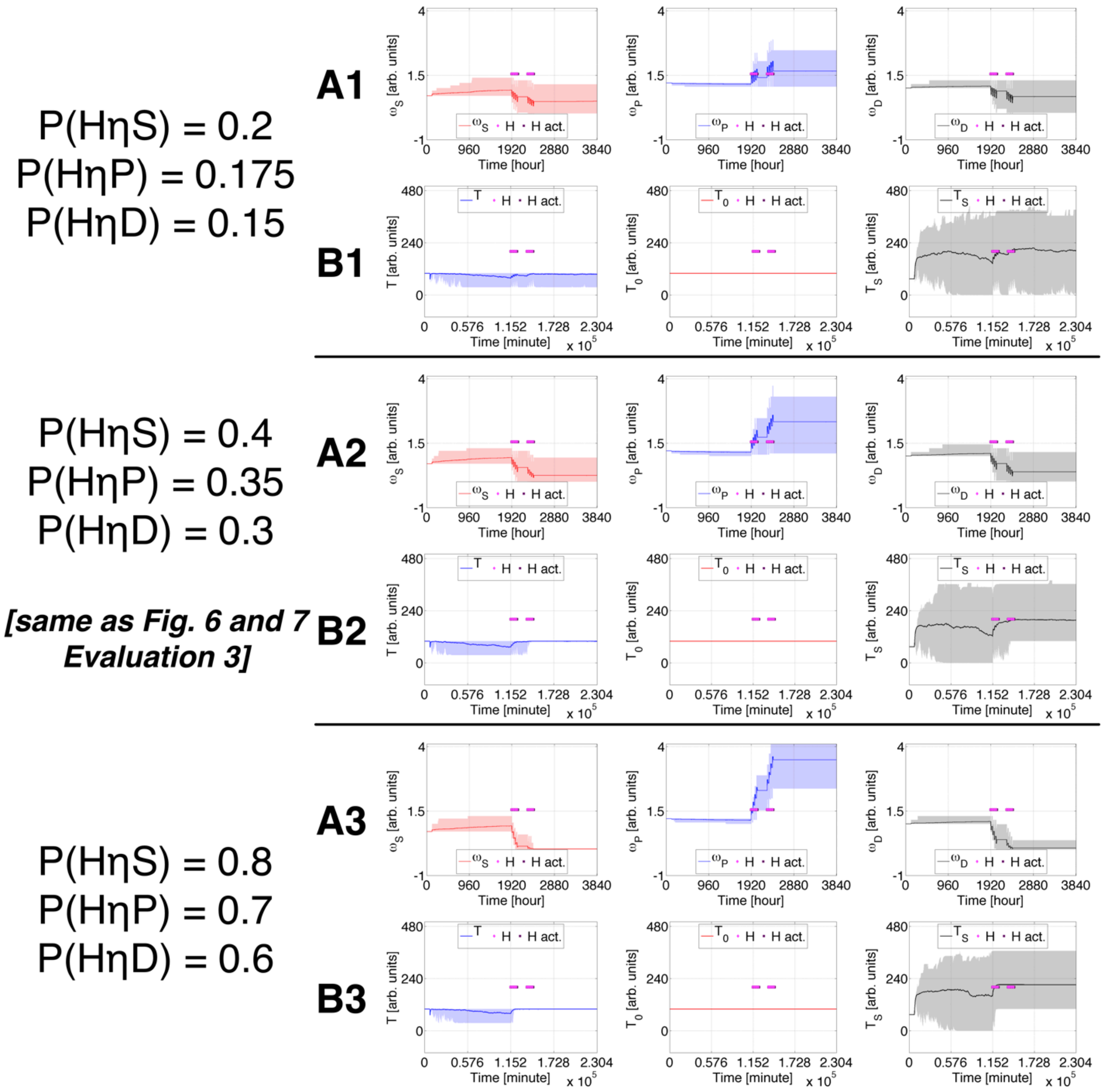

Figure S31: Comparison of different probabilities defining the durability of *H* for alternative treatments, with T_0 constant. Simulations of virtual behavior for cigarette consumption over a period of 160 days. Cigarettes are available on the fifth day. Results are the average of 100 runs. The recovery process *H* is activated at $t \in \{1920, 1960, 2000, 2040, 2280, 2320, 2360, 2400\}$ [hours] (in light pink) and stays active for 15 hours (dark pink). These simulations use the same experimental setup employed in Figures S12 and S13 but have constant T_0. At the end of each therapeutic session, the positive influence that *H* exerts on ω_S, ω_P, and ω_D become permanent or disappears, depending on arbitrary probabilities. Three sets of probabilities $P(H\eta i)$, with $i \in \{S, P, D\}$, are tested: [0.2, 0.175, 0.15], [0.4, 0.35, 0.3], and [0.8, 0.7, 0.6]. Rows A1-A3 report the evolution of cognitive weights ω_S (red), ω_P (blue), and ω_D (black); rows B1-B3 the progression of T (blue), T_0 (red), and T_S (black); column C the virtual subject's mood; and column D the average number of drug intakes (red), and the abstinence index (blue) over time. The shades correspond to 95% simulation envelopes. The simulation time-scales are multiple: hours for rows A1-A3, and minutes for rows B1-B3.

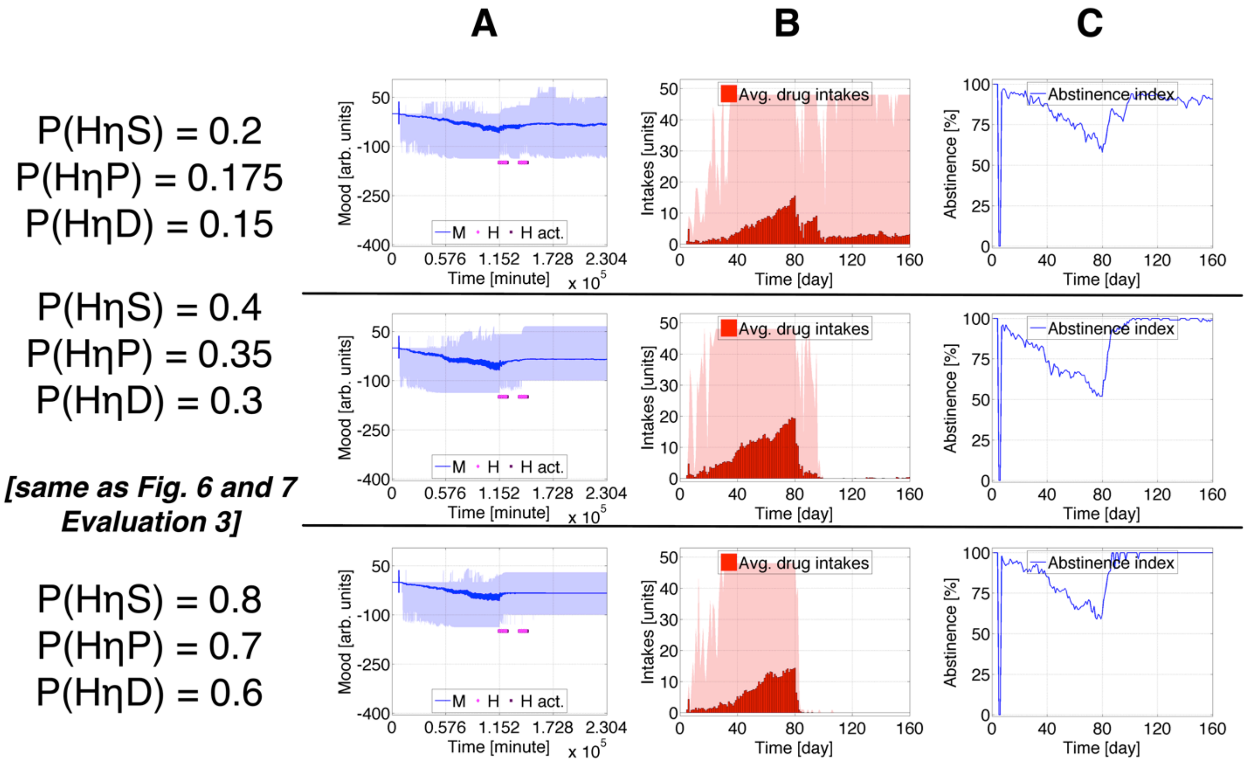

Figure S32: Comparison of different probabilities defining the durability of *H* for alternative treatments, with T_0 constant. Simulations of virtual behavior for cigarette consumption over a period of 160 days. Cigarettes are available on the fifth day. Results are the average of 100 runs. The recovery process *H* is activated at $t \in$ {1920, 1960, 2000, 2040, 2280, 2320, 2360, 2400} [hours] (in light pink) and stays active for 15 hours (dark pink). These simulations use the same experimental setup employed in Figures S12 and S13 but have constant T_0. At the end of each therapeutic session, the positive influence that *H* exerts on ω_S, ω_P, and ω_D become permanent or disappears, depending on arbitrary probabilities. Three sets of probabilities $P(H\eta i)$, with $i \in$ {S, P, D}, are tested: [0.2, 0.175, 0.15], [0.4, 0.35, 0.3], and [0.8, 0.7, 0.6]. Column A reports the virtual subject's mood; column B the average number of drug intakes, and column C the abstinence index over time. The shades correspond to 95% simulation envelopes. The simulation time-scales are multiple: minutes for column A, days columns B and C.

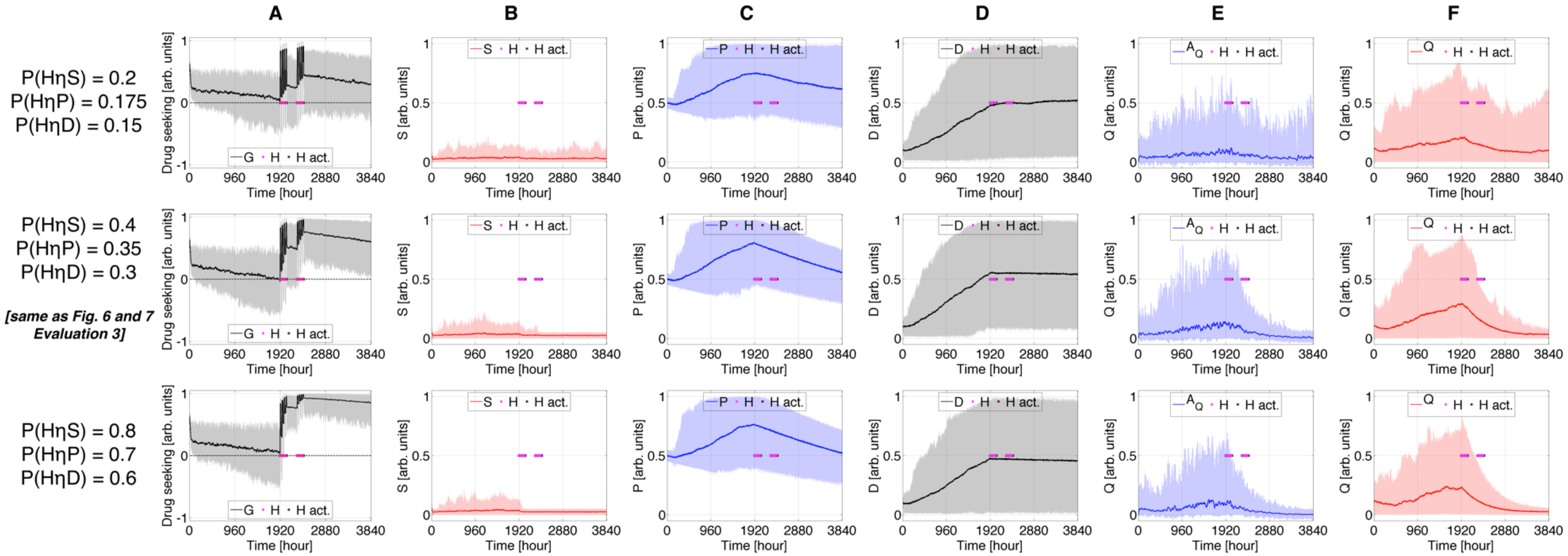

Figure S33: Details of simulations presented in Figures S31 and S32. Simulations of virtual behavior for cigarette consumption over a period of 160 days. Cigarettes are available on the fifth day. Results are the average of 100 runs. The recovery process H is activated at $t \in \{1920, 1960, 2000, 2040, 2280, 2320, 2360, 2400\}$ [hours] (in light pink) and stays active for 15 hours (dark pink). These simulations use the same experimental setup employed in Figures S12 and S13 but have constant T_0. At the end of each therapeutic session, the positive influence that H exerts on ω_S, ω_P, and ω_D become permanent or disappears, depending on arbitrary probabilities. Three sets of probabilities $P(H\eta i)$, with $i \in \{S, P, D\}$, are tested: [0.2, 0.175, 0.15], [0.4, 0.35, 0.3], and [0.8, 0.7, 0.6]. Column A reports the evolution of drug-seeking behavior G; columns B-D the progression of internal processes stress S (red), pain P (blue), and drug craving D (black); columns E-F the external trigger acute drug cue AQ (blue) and the internal process of saliency to drug cues Q (red). The shades correspond to 95% simulation envelopes.

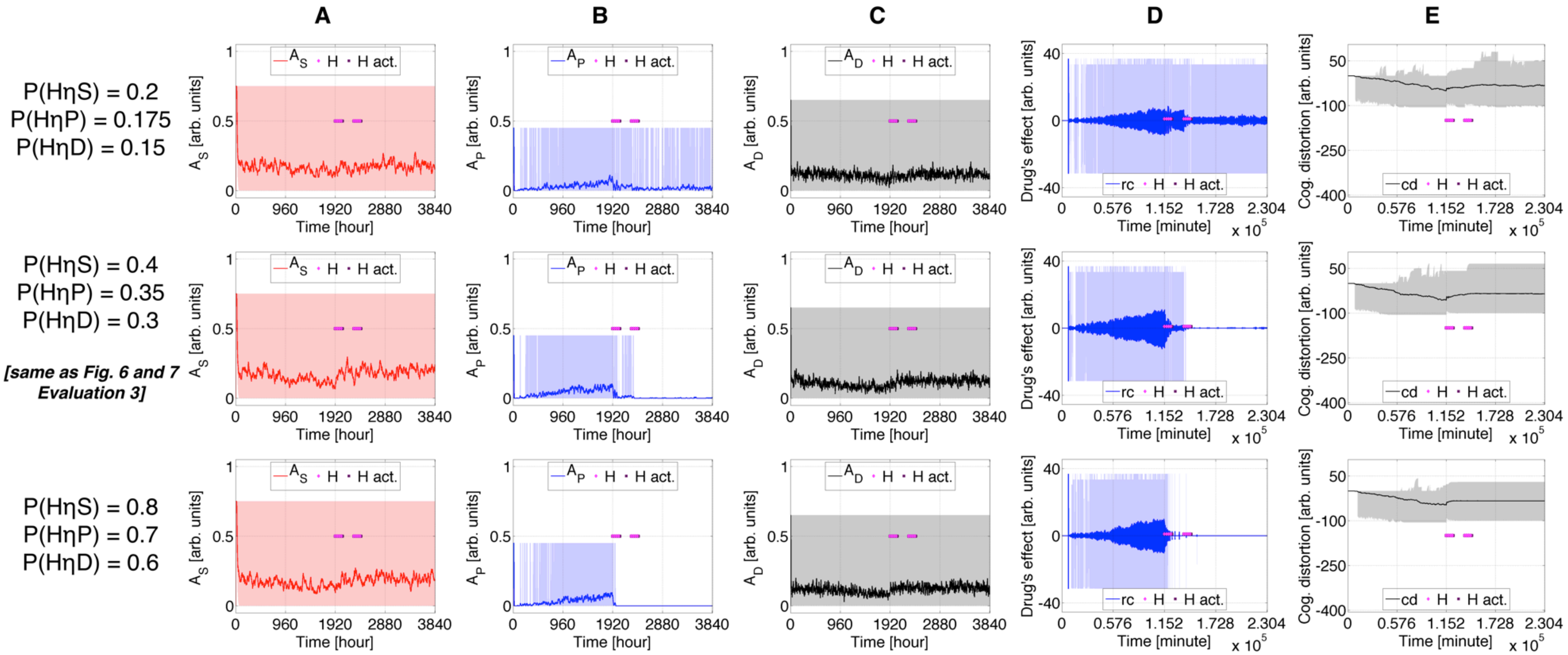

Figure S34: Details of simulations presented in Figures S31 and S32. Simulations of virtual behavior for cigarette consumption over a period of 160 days. Cigarettes are available on the fifth day. Results are the average of 100 runs. The recovery process *H* is activated at $t \in \{1920, 1960, 2000, 2040, 2280, 2320, 2360, 2400\}$ [hours] (in light pink) and stays active for 15 hours (dark pink). These simulations use the same experimental setup employed in Figures S12 and S13 but have constant T_0. At the end of each therapeutic session, the positive influence that *H* exerts on ω_S, ω_P, and ω_D become permanent or disappears, depending on arbitrary probabilities. Three sets of probabilities $P(H\eta i)$, with $i \in \{S, P, D\}$, are tested: [0.2, 0.175, 0.15], [0.4, 0.35, 0.3], and [0.8, 0.7, 0.6]. Columns A-C report the external triggers of acute shock *AS* (red), acute trauma *AP* (blue), and acute drug priming *AD* (black); column D the rush/comedown effect *rc* of drug intakes; and column E the cognitive distortion *cd*. The shades correspond to 95% simulation envelopes. The abscissas correspond to the simulation's time-steps: hours in columns A-C, minutes in columns D and E.
